# Tks5 interactome reveals endoplasmic‐reticulum‐associated translation machinery in invadosomes

**DOI:** 10.1111/febs.70196

**Published:** 2025-07-24

**Authors:** Léa Normand, Benjamin Bonnard, Margaux Sala, Sylvaine Di Tommaso, Cyril Dourthe, Anne‐Aurélie Raymond, Jean‐William Dupuy, Luc Mercier, Jacky G. Goetz, Violaine Moreau, Elodie Henriet, Frédéric Saltel

**Affiliations:** ^1^ Inserm, UMR1312, BRIC, BoRdeaux Institute of onCology, University of Bordeaux France; ^2^ CNRS, INSERM, TBM‐Core, US5, UAR 3427, OncoProt, University of Bordeaux France; ^3^ Bordeaux Proteome University of Bordeaux France; ^4^ Inserm U1109, MN3T Strasbourg France; ^5^ Université de Strasbourg France; ^6^ LabEx Medalis Université de Strasbourg France; ^7^ Fédération de Médecine Translationnelle de Strasbourg (FMTS) France

**Keywords:** cancer, ER, invadosomes, Tks5, translation

## Abstract

The ability to progress and invade through the extracellular matrix is a characteristic shared by both normal and cancer cells through the formation of structures called invadosomes, which include invadopodia and podosomes. These invadosomes are plastic and dynamic structures that can adopt different organizations—such as rosettes, dots, or linear invadosomes—depending on the cell types and the environment. In this study, we used the specific invadosome marker SH3 and PX domain‐containing protein 2A (SH3PXD2A; also known as Tks5) to identify common features in these different organizations. Tks5 immunoprecipitation coupled with mass spectrometry analysis allowed us to identify common proteins in these different models. We identified elements of the translation machinery, in particular the eukaryotic translation initiation factor 4B (EIF4B) protein, but also endoplasmic reticulum (ER) proteins as part of the invadosome structure. Providing new data on invadosome molecular composition through the Tks5 interactome, we identified that ER‐associated translation machinery is recruited to invadosomes and involved in their formation, persistence, and function in all types of invadosomes.

## Graphical abstract

Invadosomes—dynamic structures enabling matrix invasion—include rosettes, dots, and linear forms, depending on cell type and context. Using the invadosome marker Tks5 (SH3PXD2A), we identified shared molecular features across cell types. Mass spectrometry revealed components of the translation machinery, notably EIF4B, and ER‐associated proteins within invadosomes. Our findings highlight ER‐associated translation as a core feature of invadosome composition, contributing to their formation, stability, and function across different cellular contexts.

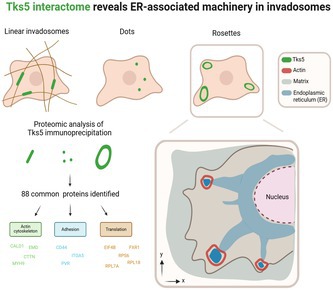

**Abbreviations**
ADAM15: adam metallopeptidase domain 15
CHX: cycloheximide
CLEM: correlative light‐electron microscopy
CTTN: cortactin
DDR1: discoidin domain receptor 1
ECM: extracellular matrix
EIF4B: eukaryotic translation initiation factor 4B
ER: endoplasmic reticulum
GFP: green fluorescent protein
GSEA: gene set enrichment
IGF2BP2: insulin‐like growth factor 2 mRNA‐binding protein 2
LAMP1: lysosomal associated membrane protein 1
LC–MS/MS: liquid chromatography coupled to tandem mass spectrometry
MAP4: microtubule‐associated protein 4
MMP14: matrix metalloproteinase 14
N‐WASP: neuronal Wiskott‐Aldrich syndrome protein
RPL12: ribosomal protein 12
RPS6: ribosomal subunit S6
RTN4: reticulon 4
SH3PXD2A: SH3 and PX domain‐containing protein 2A
TSPAN6: tetraspanin 6

## Introduction

The ability to progress and invade through the surrounding environment is a characteristic shared by both normal and tumor cells. Extracellular matrix (ECM) breaching such as basement membrane or fibrillar collagen‐rich matrices is needed in physiological conditions such as embryogenesis or wound healing but is also required at the early stages of the metastatic cascade. During metastasis, cells will invade their surrounding matrix thanks to invadosomes. Invadosomes are dynamic actin‐based structures that allow cells to interact and remodel the ECM through the recruitment of metalloproteinases [1, 2]. Invadosome formation has been shown to depend on different factors such as soluble factors such as growth factors [3, 4, 5] or the mechanical properties [6, 7] and composition of the ECM [8]. These structures, classified as podosomes and invadopodia, respectively in normal cells and tumor cells, can adopt different organizations such as rosettes, aggregates, or dots [6]. These plastic and dynamic structures are able to sense and adapt to their environment composition. Laminin can promote the formation of dots [9, 10], while fibrillar type I collagen matrix leads to a reorganization along the fibers forming linear invadosomes [9, 11] demonstrating a microenvironment‐induced remodeling. This morphological plasticity is associated with a variation of the molecular composition of the structures: Podosomes present a ring of adhesive proteins which is absent in invadopodia or linear invadosomes [11]. While podosomes and invadopodia are integrin‐dependent structures, linear invadosome formation specifically depends on the discoidin domain receptor 1 (DDR1) [9, 12]. On the other hand, actin‐binding proteins such as cortactin, the Arp2/3 complex, the neuronal Wiskott–Aldrich syndrome protein (N‐WASP) and the GTPase Cdc42 are shared by all invadosome structures, but are also present in other actin structures [13, 14, 15]. However, the Tks5 scaffolding protein appears as a specific and common marker to all types of invadosomes [15]. Indeed, this protein is enriched in invadosomes but not in focal adhesion, filopodia, membrane ruffle, or lamellipodia [16]. Tks5, a Src substrate protein, is required for invadosome formation [16]. Tks5 was discovered by Lock *et al*. in 1998 [17] and is part of the p47 family. It contains two major domains, the PX one that allows anchoring to the membrane and the SH3 domain that allows protein–protein interaction. In addition to its major role in invadosome formation, Tks5 is also involved in cell division in bladder cancer and in cell proliferation via its action during the G1 phase of the cell cycle [18, 19]. Tks5 belongs to a family containing also the Tsk4 protein involved in ECM degradation and in podosomes formation, while Tks5 is involved in all invadosome formation [20].

Even if several studies over the last few years have aimed at better defining the molecular composition involved in invadosome formation [21, 22], the common or specific markers for each structure in regard to their plasticity need to be investigated.

The aim of this article was to identify new and common molecular components present in all types of invadosome forms, namely rosettes, dots, and linear invadosomes. To do so, we performed Tks5 interactome in different invadosome models. We used two cellular models, a murine fibroblast cell line expressing a constitutively activated form of the oncogene Src, NIH3T3‐Src, and a human epidermoid carcinoma cell line, A431, this in two ECM contexts to generate rosettes, dots, and linear invadosomes. Depending on the cell type, the TKS5‐GFP overexpression does not increase the number of invadosomes but increases the matrix degradation activity [15]; or could impact the number of invadosomes as in the B16 cell line [23].

We identified 88 proteins commonly enriched in the Tks5 interactome from four different types of invadosomes. While several identified proteins were already known to be involved in the formation or function of invadosomes (CD44, cortactin, ADAM15 or MMP14), we highlighted 54 new proteins not described to be involved in invadosome formation and/or function. Among these proteins, a large proportion was related to the translation machinery, confirming data obtained in a previous study [21]. We identified the eukaryotic translation initiation factor 4B (EIF4B) in invadosomes. We confirmed that translation and EIF4B are involved in the matrix degradation activity. Furthermore, we showed that the presence of endoplasmic reticulum (ER) proteins is required for invadosome persistence. These findings provide a better understanding of the invadosome regulation through the ER‐associated translation machinery.

## Results

### Invadosomes variability and plasticity

**Fig. 1:**
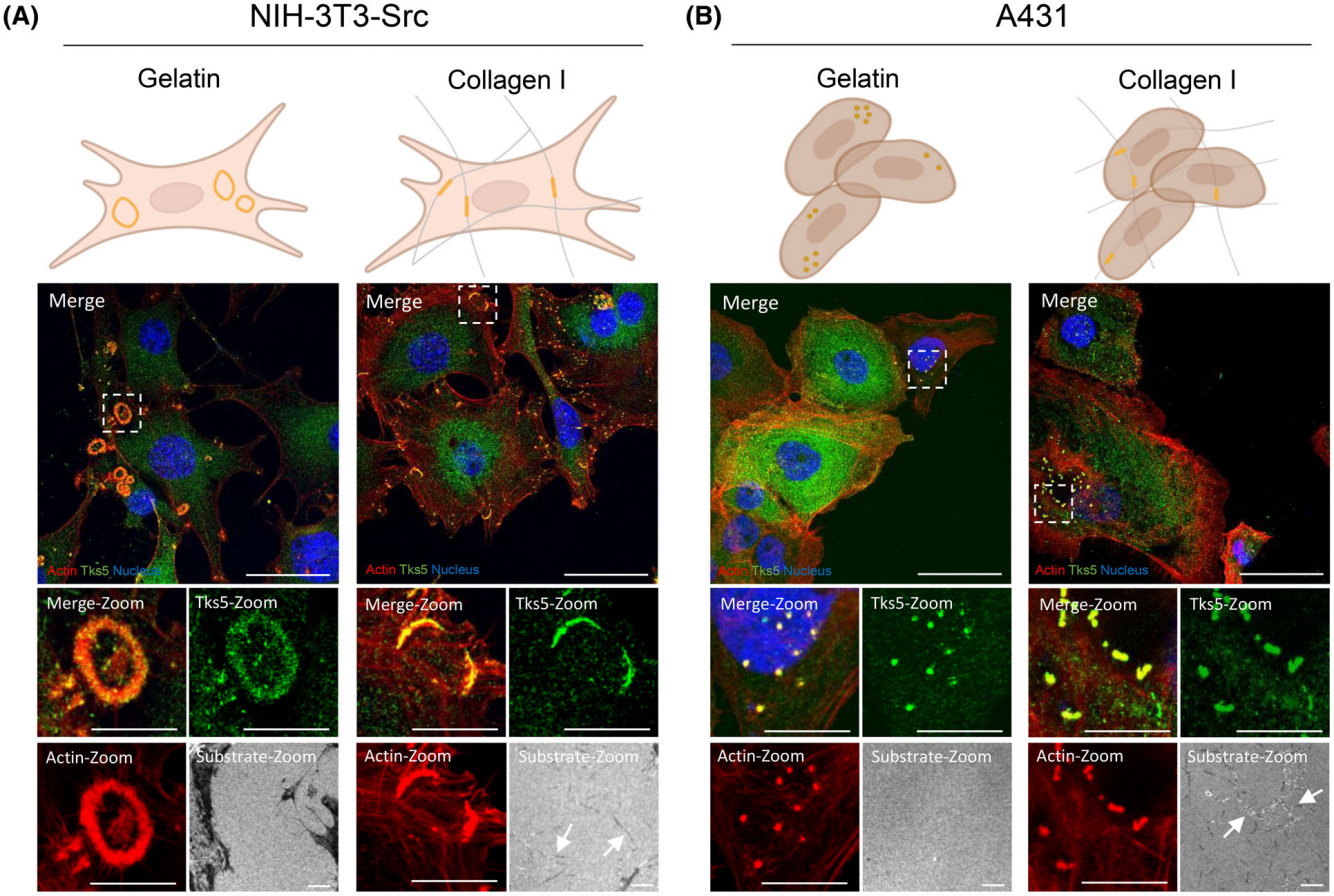
Invadosome plasticity. (A) Confocal microscopy images of invadosome formation in NIH3T3‐Src cells. The cells were seeded on gelatin or type I collagen to form rosettes and linear invadosomes respectively. Tks5 is stained in green, actin in red, nuclei in blue and collagen fibers were imaged by internal reflection microscopy (IRM). The arrows show the collagen fibers. Scale bar: 40 μm, zoom: 5 μm. *n* = 2 independent experiments. (B) Confocal microscopy images of invadosome formation in A431 cells. The cells were seeded on gelatin or type I collagen to form dots and linear invadosomes respectively. Tks5 is stained in green, actin in red, nuclei in blue and collagen was imaged by internal reflection microscopy (IRM). The arrows show the collagen fibers. Scale bar: 40 μm, zoom: 5 μm. *n* = 2 independent experiments.

In order to compare the different invadosome structures, we chose two complementary cellular models. The Src‐transformed mouse fibroblast NIH‐3T3‐Src cell line that presents invadosomes as rosettes when cultured on a gelatin substrate and the A431 human epidermoid carcinoma cell line which forms invadopodia as dots [24] (Fig. 1A,B). As expected, the fibrillar collagen matrix led to their reorganization as linear invadosomes with a colocalization of F‐actin and Tks5 along the collagen fibers in both cell lines [24] (Fig. 1A,B). These elements demonstrate the variety of shapes and plasticity of invadosomes. Thus, Tks5 is a universal marker of rosettes, dots, and linear invadosomes, and is the best candidate to investigate common invadosome features.

### Proteomics analysis of Tks5 interactome in invadosomes

**Fig. 2:**
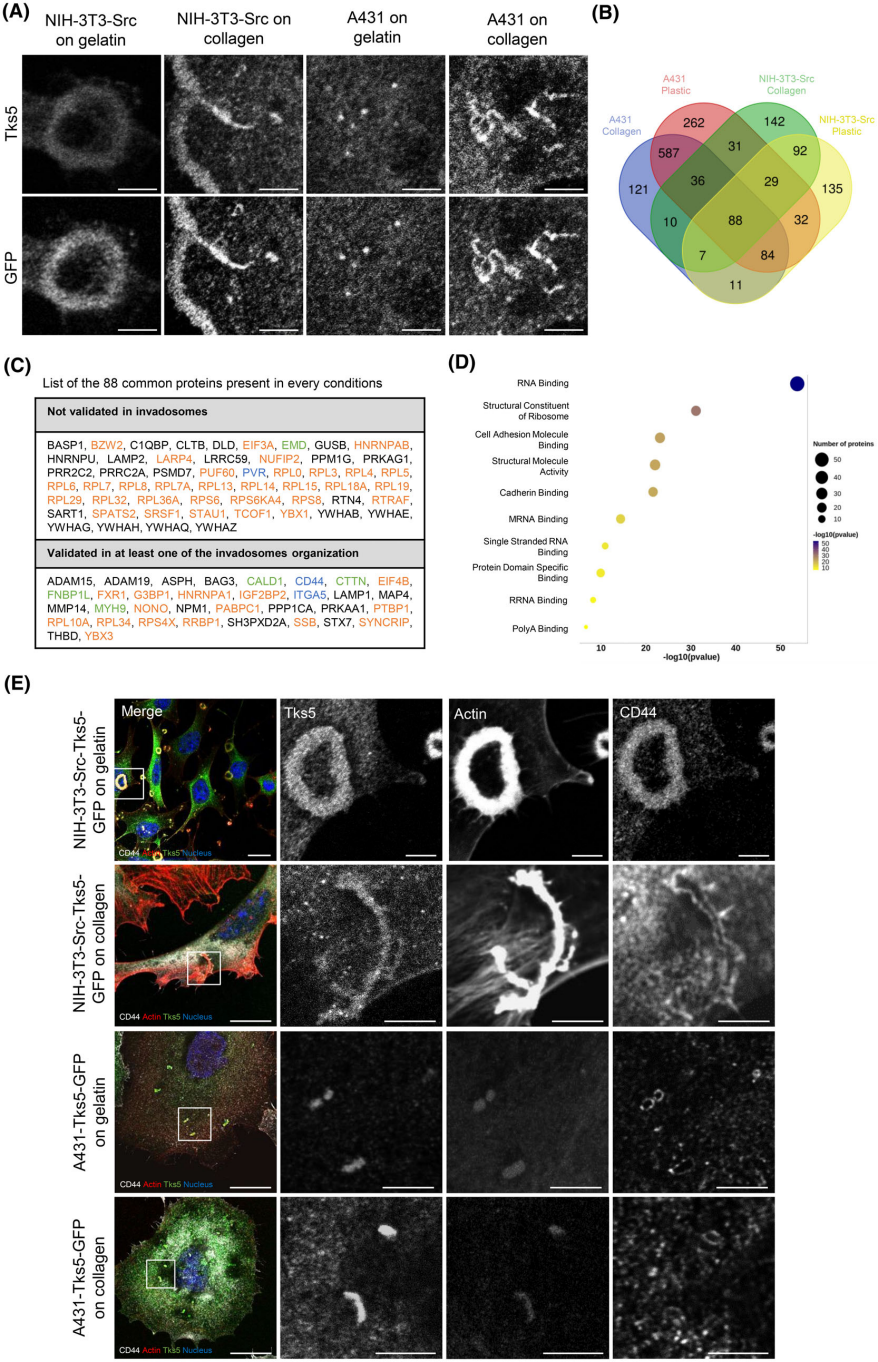
Proteomic analysis of Tks5 interactome in invadosomes. (A) Confocal microscopy images of NIH3T3‐Src‐Tks5‐GFP and A431‐Tks5‐GFP cells. The cells were seeded on gelatin and type I collagen to form invadosomes and stained for Tks5 and GFP. Scale bar: 5 μm. *n* = 2 independent experiments. (B) Proteins interacting with Tks5‐GFP by co‐immunoprecipitation in NIH3T3‐Src‐Tks5‐GFP and A431‐Tks5‐GFP cells on gelatin and type I collagen. Eighty‐eight common proteins were identified in every condition. *n* = 3 independent experiments. (C) List of the 88 common proteins identified in every condition. The color codes are in accordance with the table Fig. S2C: orange: translation, green: actin cytoskeleton and blue: adhesion. (D) Bubble plot of the proteins related pathway. (E) Confocal microscopy images of NIH‐3T3‐Src and A431‐Tks5‐GFP cells. The cells were seeded on gelatin or type I collagen and stained for Tks5 in green, actin in red, nuclei in blue, and CD44 in gray. Scale bar: 20 μm, zoom: 5 μm. *n* = 2 independent experiments.

To determine Tks5 interactome in these different invadosome types, we transfected NIH‐3T3‐Src and A431 cells with GFP‐tagged Tks5 encoding plasmid. As expected, GFP‐Tks5 colocalizes with endogenous Tks5 in all types of invadosomes (Fig. 2A). We realized immunoprecipitation against GFP in both cell lines on plastic and type I collagen conditions (Fig. S1A). Indeed, on plastic, the cells behave as on a gelatin coating and thus form the same types of invadosomes, that is, dots for A431 cells and rosettes for NIH‐3T3‐Src cells. Tks5 interactome was then assessed by LC–MS/MS mass spectrometry and the results are shown in Table S1.

We first determined the specific molecular signature associated with each invadosome organization (Tables S1–S5). We commonly identified an enrichment in mitochondrial, endoplasmic reticulum (ER), and Golgi proteins (Fig. S1B–G). The gene set enrichment analysis (GSEA) performed on the Tks5 interactome from linear invadosomes (A431 and NIH‐3T3‐Src cells) showed an enrichment for cell adhesion proteins such as CD34 and TSPAN6 absent in other invadosome organizations (Fig. S1F). The specific organization of linear invadosomes comprises both proteins shared by A431 and NIH cell lines, as well as lineage‐specific proteins unique to each. These data show that depending on each organization, invadosomes exhibit specific proteins probably involved in their structure or function. Similarly, we also showed specific enrichment of translation proteins although each is organization‐specific: EIF5A in rosettes, RPS15 in dots, or EIF2B4 in linear invadosomes (Fig. S1C,E,G).

We performed a comparative analysis with published data regarding proteins associated with Tks5 in 293T cells [22], and also identified cortactin (CTTN), the molecular chaperone Hsp90B1, the elongation factor EEF2, or the beta‐actin protein (ACTB) (Fig. S2A, left panel). Similarly, another recent study that used proximity‐labelling proteomic assay to identify Tks5 interactome in invadopodia in MDA‐MB‐231 cells also identified CTTN, the microtubule‐associated protein 4 (MAP4), the reticulon 4 protein (RTN4), the metalloproteinase ADAM15, and the eukaryotic translation initiation factor 4A3 (EIF4A3) [25] (Fig. S2A, midel panel). To finish, Zagryazhskaya‐Masson *et al*. [26] also identified proteins enriched in linear invadosomes associated with Tks5 in MDA‐MB‐231 cells by mass spectrometry. They also identified ribosomal proteins such as RPS6 or RPL12 (Fig. S2A, right panel). Since cortactin is well‐known to be present in all types of invadosomes, it can be used as a positive control to our approach [27, 28].

We then focused on proteins that were common to all invadosomes and identified 88 proteins present in dots, rosettes, and linear invadosomes in the two cell types (Fig. 2B–D). Among the 88 common proteins, 34 proteins such as CTTN, LAMP1, ADAM15, or MMP14 [28, 29, 30] were previously identified in the literature as components of invadosomes, validating our mass spectrometry experiment (Table S6). In order to validate identified proteins known to interact with Tks5 [25, 31, 32], we stained by immunofluorescence the receptor CD44 in all types of invadosomes and confirmed the colocalization of CD44 with Tks5 in all organizations of invadosomes (Fig. 2E). Interestingly, the pattern of CD44 is different in rosettes where it colocalizes with the actin, in comparison with dots and linear invadosomes where CD44 surrounds the actin. Similarly, MAP4 co‐localized with Tks5 in dots and linear invadosomes in A431 cells (Fig. S2B). These results were obtained from cells overexpressing Tks5‐GFP but were also confirmed in WT cells not overexpressing Tks5‐GFP (data not shown). These experiments confirm the correct colocalization between Tks5 and the proteins CD44 and MAP4, identified in the Tks5 interactome by mass spectrometry analysis.

However, a large proportion of identified protein has never been described to be present in invadosomes such as RPL19, RTN4, or EIF3A (Fig. 2C). The GSEA performed on the 88 common proteins showed an enrichment in translation signaling (52% of total proteins) with an enrichment of RNA binding and structural constituent of ribosome proteins (Fig. 2D), including determinant elements of translation machinery such as RPL19, EIF4B, or RPS6 (Fig. S2C). RPS6 is a component of the 40S subunit of the ribosome that allows the reading of mRNA [33]. EIF4B, on the other hand, is a translation factor that is part of the pre‐initiation complex [34]. This complex, in association with the 40S subunit, allows starting mRNA translation. This result confirms the presence of the translation machinery at invadosomes, already identified by our team in NIH‐3T3‐Src cells using proteomics on laser captured rosette‐like invadosomes [21].

All together, these results validate our experimental approach leading to the identification of new Tks5 partners into all types of invadosomes and suggest a link between Tks5 and the translation machinery.

### The translation machinery is present in all types of invadosomes

**Fig. 3:**
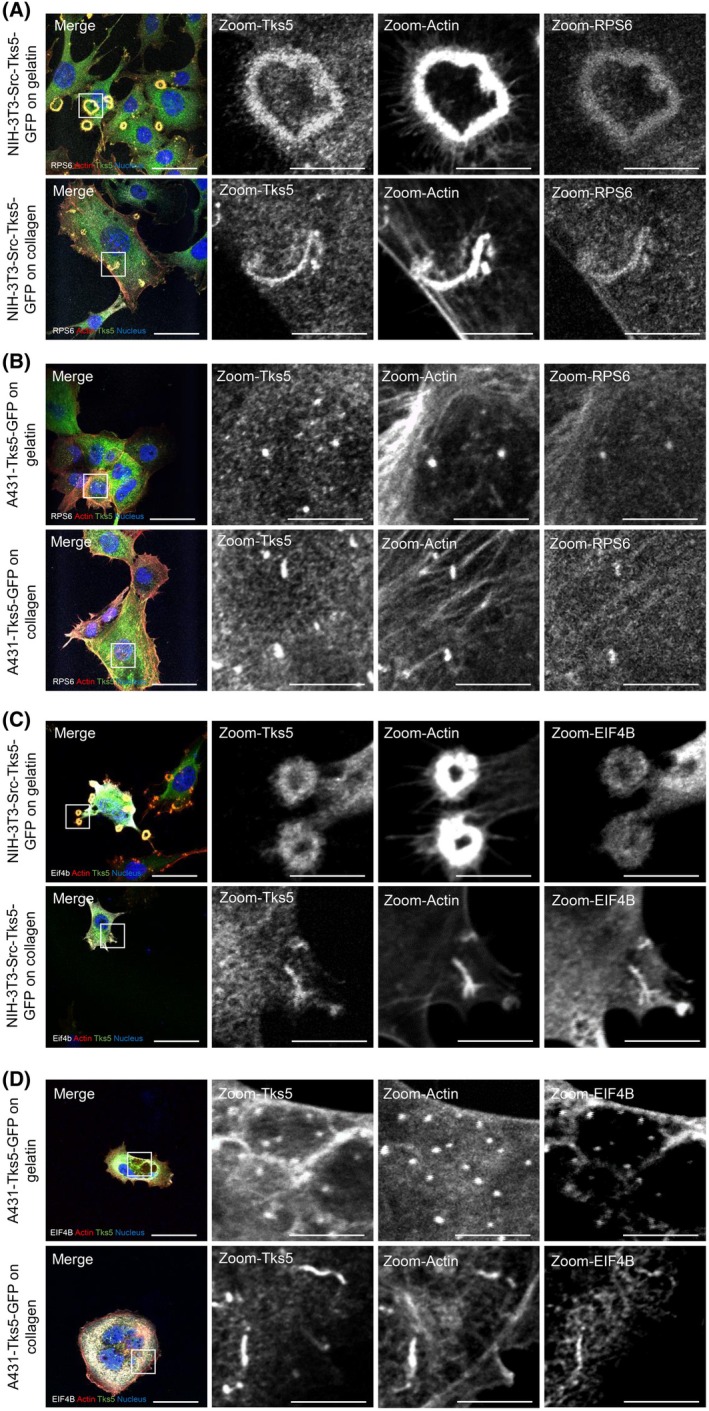
The translation machinery is present in all types of invadosomes. (A) Confocal microscopy images of NIH3T3‐Src‐Tks5‐GFP cells. The cells were seeded on gelatin or type I collagen and stained for Tks5 in green, actin in red, nuclei in blue, and RPS6 in gray. Scale bar: 40 μm, zoom: 10 μm. *n* = 2 independent experiments. (B) Confocal microscopy images of A431‐Tks5‐GFP cells. The cells were seeded on gelatin or type I collagen and stained for Tks5 in green, actin in red, nuclei in blue, and RPS6 in gray. Scale bar: 40 μm, zoom: 10 μm. *n* = 2 independent experiments. (C) Confocal microscopy images of NIH3T3‐Src‐Tks5‐GFP cells. The cells were seeded on gelatin or type I collagen and stained for Tks5 in green, actin in red, nuclei in blue, and EIF4B in gray. Scale bar: 40 μm, zoom: 10 μm. *n* = 2 independent experiments. (D) Confocal microscopy image of A431‐Tks5‐GFP cells. The cells were seeded on gelatin or type I collagen and stained for Tks5 in green, actin in red, nuclei in blue, and EIF4B in gray. Scale bar: 40 μm, zoom: 10 μm. *n* = 2 independent experiments.

As we identified the translation machinery as a common feature shared by all the invadosome structures, we decided to focus our analysis on these translation proteins. Interestingly, other molecular studies of invadosomes, whether global or linked to Tks5, have identified proteins linked to translation [21, 22, 25, 26, 35] (Fig. S2D). Briefly, Mallawaaratchy *et al*. identified proteins enriched in invadopodia of glioblastoma cells by mass spectrometry. Ezzoukhry *et al*. identified translation proteins enriched in invadosomes by laser microdissection combined with mass spectrometry. Stylli *et al*. identified proteins linked to Tks5 by co‐immunoprecipitation and mass spectrometry in dots of 293T cells. Thuault *et al*. identified proteins linked to Tks5 by biotinylation technique in dots of MDA‐MB‐231, and Zagryazhskaya‐Masson *et al*. identified proteins linked to Tks5 in linear invadosomes by mass spectrometry in MDA‐MB‐231 cells. The RPS6 staining and the co‐localization with Tks5 validated the presence of ribosomal proteins in all invadosomes (Fig. 3A,B). We also showed the colocalization of the translational initiation factor, EIF4B, which was previously found enriched in rosettes [21], with Tks5 in all organizations of invadosomes in both cell lines (Fig. 3C,D). The presence of ribosomal proteins and translation factors in all invadosomes supports the hypothesis of active translation in all these structures. Moreover, comparative analysis, using the dataset obtained from the Tks5 interactome from NIH‐3T3‐Src cells harboring rosettes and the published rosettes proteome [21], showed an enrichment in ribosomal and translation proteins (Fig. S2E,F). We commonly identified 79 proteins, including 39 related to translation proteins (Fig. S2F). Among these 39 proteins, 20 are ribosomal proteins (Fig. S2F), which confirms the presence of ribosomes in invadosome rosettes as observed in Ezzoukhry *et al*.

### Involvement of the translation machinery in invadosome function

**Fig. 4:**
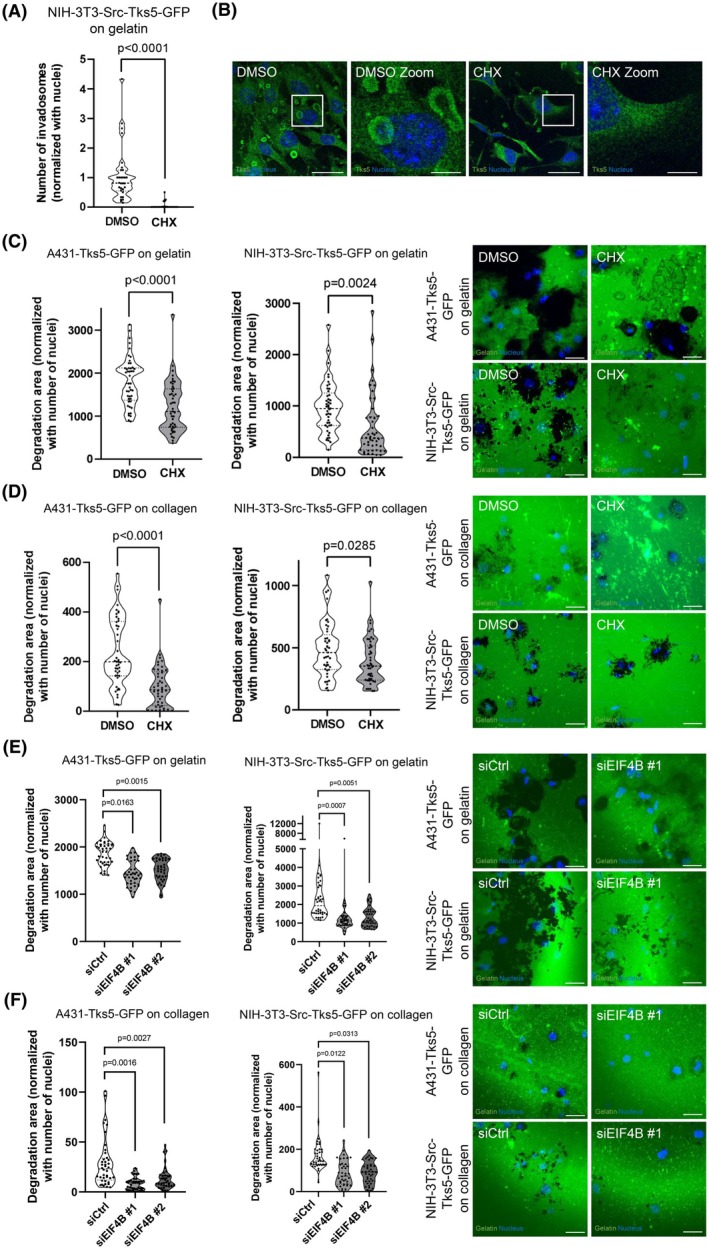
Involvement of the translation machinery in invadosome activity. (A) Quantification of the numbers of invadosomes per cell treated (CHX) or not (DMSO) with cycloheximide in NIH3T3‐Src‐Tks5‐GFP cells seeded on gelatin. Values represent the mean ± SEM of *n* = 3 independent experiments (10 images per condition and per replicate) and were analyzed using Student's *t*‐test. (B) Representative images of the quantification of invadosome formation in NIH3T3‐Src‐Tks5‐GFP cells seeded on gelatin. Tks5 is stained in green and nuclei in blue. Scale bar: 40 μm, zoom: 10 μm. (C) Left: Quantification of the ECM degradation properties of A431 and NIH3T3‐Src‐Tks5‐GFP cells on gelatin treated (CHX) or not (DMSO) with cycloheximide. Values represent the mean ± SEM of *n* = 3 independent experiments (15 images per condition and per replicate) and were analyzed using Student's *t*‐test. Right: Representative images of the quantification of ECM degradation of A431 and NIH3T3‐Src‐Tks5‐GFP cells on gelatin. Gelatin is stained in green and nuclei in blue. Scale bar: 40 μm. (D) Left: Quantification of the ECM degradation properties of A431 and NIH3T3‐Src‐Tks5‐GFP cells on collagen treated (CHX) or not (DMSO) with cycloheximide. Values represent the mean ± SEM of *n* = 3 independent experiments (15 images per condition and per replicate) and were analyzed using Student's *t*‐test. Right: Representative images of the quantification of ECM degradation of A431 and NIH3T3‐Src‐Tks5‐GFP cells on collagen. Gelatin is stained in green and nuclei in blue. Scale bar: 40 μm. (E) Left: Quantification of the ECM degradation properties of A431 and NIH3T3‐Src‐Tks5‐GFP cells on gelatin treated (siEIF4B#1 and #2) or not (siCtrl) with siEIF4B. Values represent the mean ± SEM of *n* = 3 independent experiments (15 images per condition and per replicate) and were analyzed using Student's *t*‐test. Right: Representative images of the quantification of ECM degradation of A431 and NIH3T3‐Src‐Tks5‐GFP cells on gelatin. Gelatin is stained in green and nuclei in blue. Scale bar: 40 μm. (F) Left: Quantification of the ECM degradation properties of A431 and NIH3T3‐Src‐Tks5‐GFP cells on collagen treated (siEIF4B#1 and #2) or not (siCtrl) with siEIF4B. Values represent the mean ± SEM of *n* = 3 independent experiments (15 images per condition and per replicate) and were analyzed using Student's *t*‐test. Right: Representative images of the quantification of ECM degradation of A431 and NIH3T3‐Src‐Tks5‐GFP cells on collagen. Gelatin is stained in green and nuclei in blue. Scale bar: 40 μm. ECM, extracellular matrix.

In order to investigate and confirm the role of translation in all invadosome formation and matrix degradation activity from both cell lines, we treated A431 and NIH‐3T3‐Src cells seeded on gelatin or type 1 collagen with the translation inhibitor cycloheximide (CHX) (Fig. S3). Our previous study demonstrated that CHX blocked the formation of rosettes in a short time, after 1 h of treatment [21]. By using western blot and Surface Sensing of translation (SUnSET) assay, we first showed that CHX treatment reduced global translation in all conditions (Fig. S3A,B). We next confirmed that CHX treatment in the NIH‐3T3‐Src model prevented invadosome rosette formation (Fig. 4A,B) as previously described [21]. No significant differences in dots and linear invadosome formation were measured by immunofluorescence quantification between the CHX and control groups (DMSO) (Fig. S3C,D). However, CHX treatment limited invadosome degradation activity by A431 and NIH‐3T3‐Src cells on gelatin and collagen matrices (Fig. 4C,D). These results showed that global translation is involved in the degradation capacity of all invadosomes and formation only in rosette organization.

As we identified EIF4B in all invadosomes (Fig. 2C; Fig. S2C), we assessed the role of EIF4B in invadosome degradation activity. By using a small interfering RNA (siRNA) strategy, we silenced EIF4B expression in both cell lines (Fig. S3E) and demonstrated that EIF4B depletion prevented invadosome rosette formation in NIH‐3T3‐Src model and linear invadosome formation in the A431 model (Fig. S3F). However, EIF4B depletion reduced matrix degradation activity in all organizations of invadosomes from both cell lines (Fig. 4E,F). These results showed that EIF4B is involved in the degradation capacity of all invadosomes.

These data confirm the involvement of the translation and more specifically of the EIF4B protein in the matrix degradation activity of all invadosomes.

### Recruitment of ER into invadosome rosettes

In addition to translation proteins, the mass spectrometry analysis highlighted the presence of ER‐related proteins such as RTN4, LRRC59, or RRBP1 in all invadosomes linked with Tks5 (Fig. 2C). Ribosomes and ER proteins have previously been identified in the proteome of invadosome rosettes [21].

**Fig. 5:**
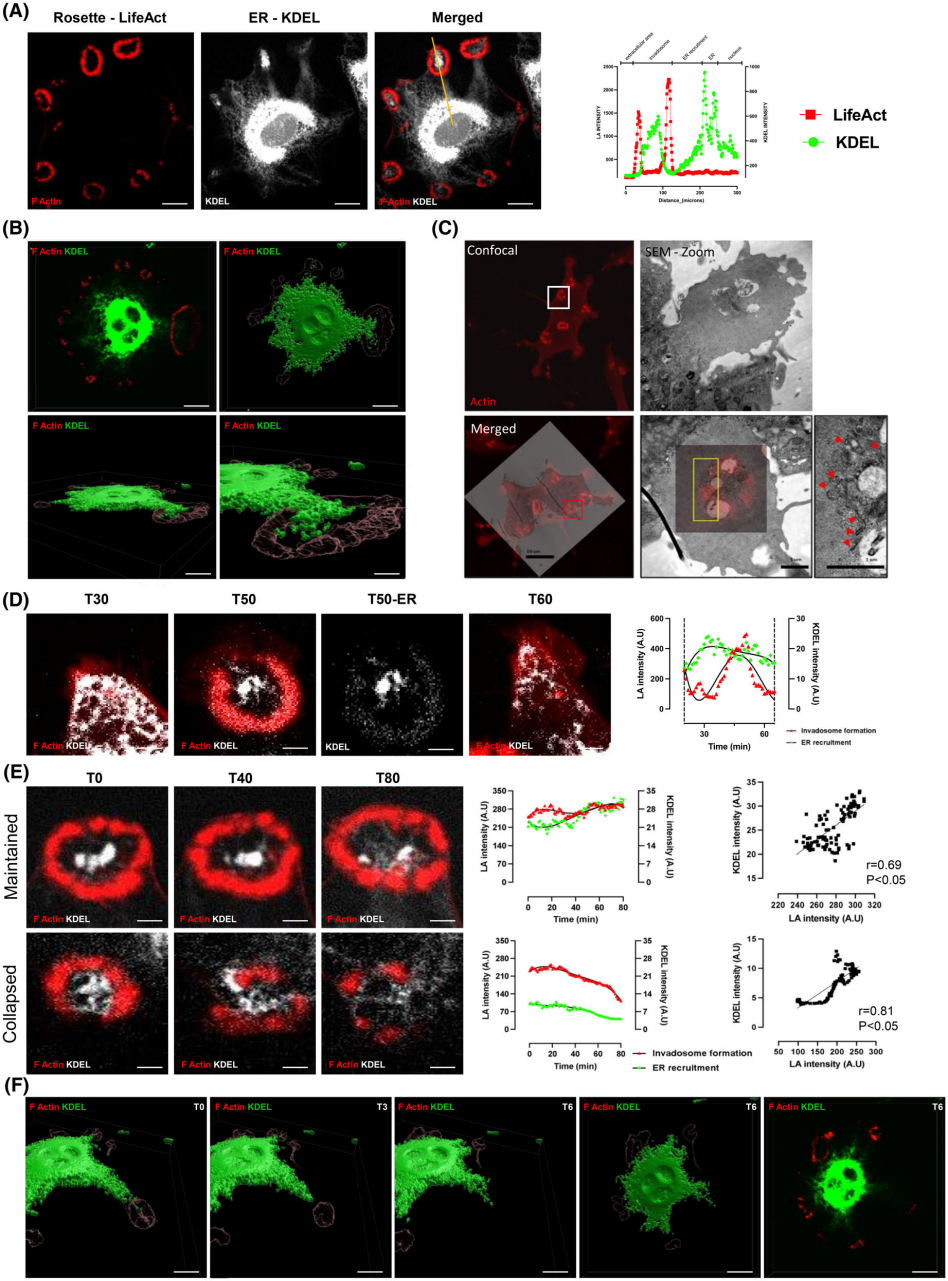
Recruitment of ER into invadosome rosettes. (A) Representative images from time‐lapse video microscopy of lifeact‐mRuby (red)‐expressing NIH‐3T3‐Src cells transfected with KDEL‐GFP (white). The lifeact‐mRuby and KDEL‐GFP were measured along the yellow axis. Scale bar: 20 μm. Images represent *n* = 3 independent experiments (10 images per condition and per replicate). (B) Projection of a z‐stacks and Imaris surface 3D renderings are shown, visualizing that KDEL‐GFP (green) localizes inside invadosome during maturation. Scale bar: 20 μm, bottom right panel: 10 μm. Images represent *n* = 3 independent experiments (10 images per condition and per replicate). (C) CLEM images of invadosomes of NIH‐3 T3‐Src cells on gelatin matrix. Actin is stained in red. Red arrows show the endoplasmic reticulum. Scale bar: 10 and 1 μm. Images represent *n* = 3 independent experiments (10 images per condition and per replicate). (D) Representative images from time‐lapse video microscopy of lifeact‐mRuby (red)‐expressing NIH‐3T3‐Src cells transfected with KDEL‐GFP (white). The intensity level of lifeact‐mRuby and KDEL‐GFP was measured in the initiation step in rosette. Scale bar: 5 μm. Images represent *n* = 3 independent experiments (10 images per condition and per replicate). (E) Representative images from time‐lapse video microscopy of lifeact‐mRuby (red)‐expressing NIH‐3T3‐Src cells transfected with KDEL‐GFP (white). The intensity level of lifeact‐mRuby and KDEL‐GFP was measured in maintained and collapsed rosettes. Scale bar: 5 μm. Images represent *n* = 3 independent experiments (10 images per condition and per replicate). (F) Projection of a z‐stacks and Imaris surface 3D renderings are shown, visualizing that KDEL‐GFP (green) escapes from invadosome during its collapse. Scale bar: 20 μm. Images represent *n* = 3 independent experiments (10 images per condition and per replicate). ER, endoplasmic reticulum.

In order to assess the involvement of the ER in invadosome formation and function, we used the rosette model, which is the largest invadosome structure, facilitating analysis and imaging. We used lifeact‐mRuby‐expressing NIH‐3T3‐Src cells transfected with KDEL‐GFP to detect ER presence. By video microscopy, we showed that the ER was recruited at the level of the rosette in formation and that there was an enrichment of the ER inside the actin core, as previously described in fixed cells [21] (Fig. 5A, Video S1). Furthermore, time‐lapse imaging clearly shows that the ER is not present in all the cytoplasm and revealed that the ER forms “arms” leading to a “ER flow” heading toward the forming rosette then accumulating within the actin core (Fig. 5A, Video S1). The transversal sections confirm the enrichment as we can clearly see that the ER arm is inserted within the rosette actin core (Fig. 5A). 3D reconstruction allowed us to visualize the accumulation of the ER within the rosette core (Fig. 5B). In order to confirm the presence of the ER at the level of rosettes, we performed correlative light and electron microscopy (CLEM). Correlation between actin fluorescent images and electron microscopy allowed the identification of ER tubules associated with the rosettes (Fig. 5C).

In order to determine the dynamic of ER recruitment during invadosome formation, time‐lapse imaging was performed. The analysis revealed that ER recruitment occurs before actin polymerization and the formation of rosette‐like invadosomes (Fig. 5D, Video S2). In addition, we showed that the maintenance of the structures requires the presence of ER (Fig. 5E upper panel, Video S3a) while a drop in ER recruitment is associated with a collapse of the structure (Fig. 5E lower panel, Video S3b). 3D reconstruction confirmed that the collapse of the rosette structure is associated with a withdrawal of the ER within the structure, with the “ER flow” heading toward the interior of the cell (Fig. 5F). These results, therefore, confirm the presence but also a potential involvement of the ER in the rosette formation and maintenance over time.

### Involvement of ER‐associated machinery translation in invadosome formation

**Fig. 6:**
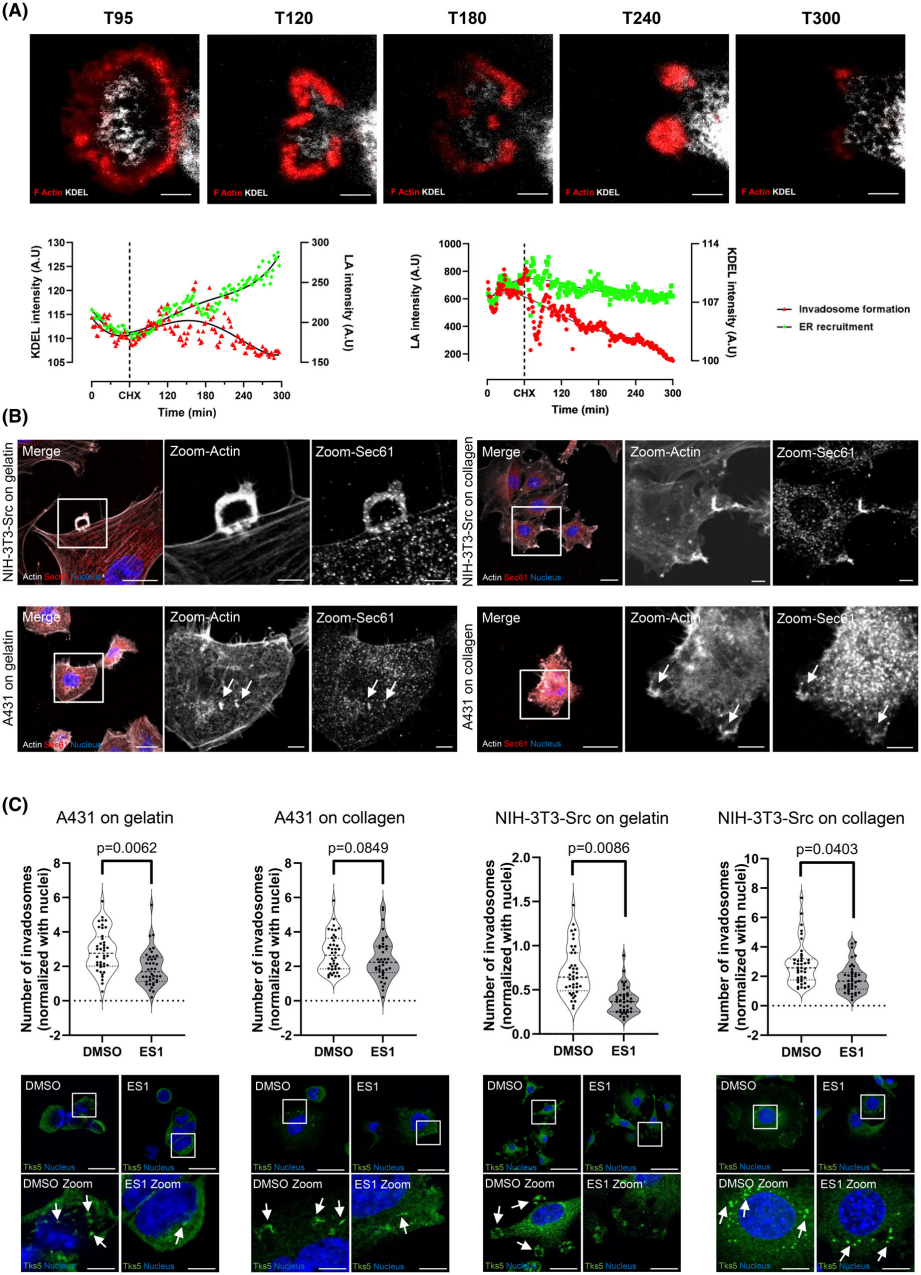
Involvement of ER‐associated machinery translation in invadosome formation. (A) Lifeact‐mRuby and KDEL‐GFP signals were recorded in NIH‐3T3‐Src cells treated with cycloheximide (CHX; 35 μm) at T60 min. Scale bar: 5 μm (T95: time 95; LA intensity: laser activation intensity). Images represent *n* = 2 independent experiments (10 images per condition and per replicate). (B) Confocal microscopy images of NIH‐3T3‐Src and A431 cells. The cells were seeded on gelatin or type I collagen and stained for Sec61 in red, nuclei in blue, and Actin in gray. The arrows show the colocalization between Sec61 and the linear invadosomes (actin). Scale bar: 20 μm, zoom: 5 μm. Images represent *n* = 2 independent experiments. (C) Upper part: Quantification of invadosome formation in A431 and NIH‐3T3‐Src cells seeded on gelatin or type 1 collagen treated or not (DMSO) with Sec61 translocon inhibitor (ES1) during 1 h. Values represent the mean ± SEM of *n* = 4 independent experiments (10 images per condition and per replicate) and were analyzed using Student's *t*‐test. Lower part: Representative images of the quantification of invadosome formation in A431 and NIH3T3‐Src‐Tks5‐GFP cells seeded on gelatin or type 1 collagen treated or not (DMSO) with ES1. The arrows show the invadosomes. Tks5 is stained in green and nuclei in blue. Scale bar: 40 μm. Images represent *n* = 4 independent experiments (10 images per condition and per replicate). ER, endoplasmic reticulum.

As we identified the presence and the determinant role of translation and ER in invadosome formation, we investigated the role of ER‐associated translation in invadosome formation and matrix degradation activity. Previously published results showed the involvement of the ER in the formation and function of invadosomes via the involvement of the protrudin protein or by the involvement of the calnexin/erp57 protein complex respectively [36, 37]. To determine the role of ER‐associated translation in the dynamic of invadosome rosettes formation, we performed a time‐lapse experiment with cells treated with CHX to inhibit translation (Fig. 6A, Video S4). Translation inhibition led to the collapse of the rosette structure while the ER was still present at the “previous invadosome site” without reformation of invadosomes (even if some actin polymerization events were recorded) (Fig. 6A, Video S4). This result suggests that ER‐associated translation is mandatory for invadosome persistence.

To further characterize the involvement ER‐associated translation in invadosome formation, we treated cells with Sec 61 translocon inhibitor (ES1), which blocks the translocation of proteins to the ER. Indeed, Sec61 is a well‐described ER marker that allows protein insertion into the ER but also is a key player for ribosomes docking to the ER [38]. Furthermore, Sec61 was identified by our mass spectrometry analysis for A431 cells on plastic and collagen (Table S1) and localized with invadosome structures (Fig. 6B). ES1 treatment on A431 and NIH‐3T3‐Src cells seeded on gelatin or type 1 collagen matrix led to a decreased number of all organizations of invadosomes (Fig. 6C). These results therefore suggest that the translation machinery must be adapted somewhat depending on the matrix context. This would explain why the translation machinery is only involved in the degradation function for dots and linear invadosomes while it is involved in the formation and degradation function for rosettes, which are larger structures.

All together, these results demonstrate that the ER‐associated translation machinery identified in the Tks5 interactome is an essential element to maintain the formation of all types of invadosomes.

## Discussion

We provided here a molecular characterization of all types of invadosomes by identifying the interactome of the common and universal invadosome marker Tks5. We used co‐immunoprecipitation coupled with mass spectrometry to identify new proteins shared by different invadosome types such as dots, rosettes, and linear invadosomes (Fig. 1). This strategy allowed us to identify proteins already known in the literature, but also to identify new ones. We showed that Tks5 is linked to translation proteins and confirm that translation is involved in the degradation function in all types of invadosomes, including linear invadosomes.

Indeed, we identified 46 translation proteins, with some already known in the literature to be involved in invadosome activity, such as IGF2BP2 or MMP14 [30, 39, 40, 41]. Regarding the other proteins identified and already described in invadosomes, microtubule‐associated proteins, and especially MAP4 and LAMP1, were described in invadopodia [25, 29, 31] and ITGA5 was described in invadopodia [35]. PTBP1 was also identified in podosomes as participating in their formation [42]. We further demonstrated here that the matrix receptor CD44, which was previously described in podosomes and invadopodia but not in linear invadosomes [32, 43] is also a common marker of invadosomes (Fig. 2). These known proteins allowed us to serve as quality control for mass spectrometry experiments.

Thanks to mass spectrometry experiments, we were also able to validate the presence of translation proteins in linear invadosomes. We identified the presence of EIF4B and RPS6 proteins in all invadosome organizations (Fig. 3). Interestingly, these two proteins are known in the literature to be targeted by the mTOR/S6K1 pathway. Indeed, S6K1 is able to directly phosphorylate EIF4B to allow the recruitment of EIF4A and EIF3 as well as the RPS6 protein to initiate translation [44]. While we demonstrated that global translation is mandatory for degradation activity induced by invadosomes, the inhibition of global translation did not prevent invadosome formation (excepted for rosettes) (Fig. 4). Nevertheless, we also showed that ER‐associated translation regulates all types of invadosome formation, suggesting that ER has a major role in invadosome formation and the associated degradation activity. We hypothesize that ribosome docking to ER, through Sec61, enhances protein synthesis of determinant elements for invadosome formation. A recent study demonstrated that an early increase of Sec61 enhanced the expression of pro‐invasive proteins, followed by an expansion of the ER and the Golgi, leading to the delivery of these pro‐invasive proteins to the cell membrane [45].

In addition to the translation proteins identified, proteins present in the ER have also been highlighted by our proteomic analysis such as RTN4 or LRRC59. Since the ER is also involved in protein translation, this suggested the presence of a complex translation mechanism within all invadosomes. We showed that the ER is recruited at the rosette, so enrichment of the ER could be linked to the rosette functioning properly, notably for its degradation capacity. In addition, our data show that removal of the ER leads to a collapse of the rosette structure, suggesting a link between the ER and invadosome formation and degradation activity. Furthermore, we showed that the ER was necessary for initiating and maintaining the invadosome rosettes (Figs 5 and 6). Indeed, the spatiotemporal aspect of invadosome formation in the initiation, stabilization, or collapse phases is a major question [46, 47]. Here, we show that the ER plays a main role in these different stages.

In literature, Pedersen *et al*. [36] also showed the involvement of ER in invadosome formation: protrudin‐mediated ER‐endosome contact site promotes cell invasion through the translocation of MT1‐MMP endosome to the plasma membrane leading to invadopodia outgrowth and degradation. Moreover, one of our recent studies showed that two ER proteins, calnexin and ERp57, can be trafficked to invadosomes and are essential for ECM degradation [37]. These results therefore suggest the presence of a complex allowing invadosomes to have their own translation machinery to facilitate cell invasion with local translation of specific mRNA [21, 48]. Tks5 could therefore act as a molecular crossroads allowing translational proteins to cluster in the same place, leading to rapid localization and greater efficiency for the cell in the formation and function of invadosomes.

In order to better understand the link between the translation and the ER in all types of invadosomes, we first targeted the EIF4B protein, identified by mass spectrometry. Using siRNA strategy, we showed that EIF4B is required for degradation function. Recent studies already showed the involvement of EIF4B in invasion. Indeed, Cao et al. [49] showed that the PIM1/EIF4B/c‐MET pathway was involved in tumor migration and invasion in lung adenocarcinoma. In the same way, the GMAN/p‐EIF4B pathway leads to cell proliferation and invasion in hepatocellular carcinoma (HCC) [50].

We also identified many proteins associated with EIF3 and EIF4 complexes such as EIF3C, EIF4A3, EIF4E, or EIF3H in at least three out of four types of invadosomes. Similarly, these complexes are already known in the literature to participate in the process of cell invasion. The YTHDF1/EIF3C axis is involved in the invasion process in ovarian cancer [51]; EIF3H participates in the invasion of hepatocellular carcinoma [52] and the EIF4A3/FLOT1 pathway promotes invasion and tumor proliferation in lung adenocarcinoma [53]. These results suggest the involvement of these complexes in the degradation mechanism that could be common to all types of invadosomes.

Altogether, these results provide new knowledge specifically into invasive structures and on the invasion process more broadly. Indeed, we reported new molecular components common to all types of invadosomes through the identification of the Tks5 interactome. This thus provides a better understanding of the mode of operation of invadosomes, in particular via the involvement of the ER‐associated translation machinery, which is involved in invadosome formation.

## Materials and methods

### Antibodies and reagent

Anti‐GFP (catalog number ab6673) antibody was purchased from Abcam (Cambridge, UK). Anti‐Tks5 (G‐7, catalog number sc‐376211) and anti‐CD44 (DF1485, catalog number sc‐7297) were purchased from Santa Cruz Biotechnology, Inc (Dallas, TX, USA). Anti‐MAP4 (AG1741, catalog number 11229‐1‐AP) was purchased from Proteintech (Rosemon, IL, USA). Anti‐RPS6 (S369, catalog number AP22299a) was purchased from Abgent (San Diego, CA, USA). Anti‐DDK (OTI2F7, catalog number TA500288) was purchased from OriGene Technologies, Inc (Rockville, MD, USA). Anti‐EIF4B (catalog number 3592) was purchased from Cell Signaling Technology (Danvers, MA, USA). Secondary antibodies for immunofluorescence IRDye^®^ 680RD Conjugated Goat anti‐mouse (926‐68070; LI‐COR Biotech, Homburg, Germany), IRDye^®^ 680RD Conjugated Goat anti‐rabbit (926‐68071; LI‐COR Biotech), IRDye^®^ 800CW Conjugated Goat anti‐mouse (926‐32210; LI‐COR Biotech), IRDye^®^ 800CW Conjugated Goat anti‐rabbit (926‐32211; LI‐COR Biotech). The Atto 647N conjugated Goat anti‐mouse‐IgG was purchased from Sigma‐Aldrich (Saint‐Louis, MO, USA) (catalog number 50185). DAPI (catalog number H21486) and phalloidin (catalog number FP‐BZ‐9630) were purchased from Interchim (Montluςon Cedex, France). Type I collagen from rat tail (catalog number 354236) was purchased from Corning (Corning, NY, USA). Sec61 translocon was inhibited by a pharmacological approach using Eeyarestatin 1 (ES1, catalog number E1286; Sigma‐Aldrich) at 5 μm during 1 h. Species‐specific fluorescent far‐red coupled secondary antibody for western blot IRDye 680CW goat (polyclonal) anti‐rabbit IgG (H + L) (catalog number 926‐68070) or IRDye 800CW goat (polyclonal) anti‐mouse IgG (H + L) (catalog number 926‐32210) were purchased from LI‐COR Biotech. GM6001 MMP Inhibitor (catalog number CC10), Cycloheximide (CHX) (catalog number 01810) and Manganese (II) (catalog number M7899) were purchased from Sigma‐Aldrich.

### Cell culture

The A431 (RRID: CVCL_0037) cell line was generously donated by J. Dechanet‐Merville (CNRS 5164, Bordeaux). NIH‐3T3‐Src cells (RRID: CVCL_L992) were generously donated by S. Courtneidge (Université of Portland, USA). These cell lines were authenticated by American Type Culture Collection (Manassas, VA, USA). A431 and NIH‐3T3‐Src cells were maintained in DMEM high glucose (Gibco, ThermoFisher Scientific, Waltham, MA, USA) supplemented with 10% fetal bovine serum (FBS, 139; Sigma‐Aldrich) at 37 °C in a 5% CO_2_ incubator. In this study, we used A431 and NIH‐3T3‐Src cells transduced with a lentiviral plasmid expressing Tks5 fused to GFP, and the cell lines were named A431‐Tks5‐GFP and NIH3T3‐Src‐Tks5‐GFP. All experiments were performed with mycoplasma‐free cells.

### Lentiviral transduction

A431 and NIH‐3T3‐Src cells were seeded in a six‐well plate, then transduced after 24 h at a multiplicity of infection of 10 μg, either with a lentiviral plasmid containing Tks5 coupled to GFP, or with the GFP alone plasmid encoding as a control. The transduced cells were selected using puromycin treatment at 1 μg·mL^−1^. Co‐transduction of lifeact‐mRuby and KDEL‐GFP NIH‐3T3‐Src cells has been performed as described above, but double‐positive cells were selected and sorted using a BD FACSAria Cell Sorter (BD Bioscience, Milpitas, CA, USA).

### Transfection

EIF4B‐DDK plasmid was purchased from OriGene Technologies, Inc (Rockville, MD, USA). This plasmid was transfected (2.4 μg) using JetPrime (PolyPlus Transfection, Illkirch‐Graffenstaden, France) following the manufacturer's instructions. siRNA oligonucleotides (20 nm) targeting eEIF4B (Human: s4573 & s4574; Mouse: s904 & s93805; ThermoFisher) were transfected using Lipofectamine RNAiMax (Invitrogen) according to the manufacturer's instructions.

### Coating of coverslips with extracellular matrix

Cells were seeded on gelatin coverslips with or without a coating of collagen. Type I collagen (rat tail, catalog number 354236; Corning) was diluted in DPBS containing calcium and magnesium (Dulbecco's Buffered‐Phosphate Saline; Gibco, ThermoFisher Scientific) at a final concentration of 0.5 mg·mL^−1^ and polymerized for 4 h at 37 °C. Gelatin‐coated coverslips were made using gelatin from Sigma‐Aldrich (catalog number G1393). Coverslips were incubated with gelatin for 20 min before being fixed with 0.5% glutaraldehyde (Electron Microscopy Science, Hatfield, PA, USA) for 40 min at room temperature. Coverslips were washed three times with PBS before cell seeding.

The methods described here have also been detailed in previous publications. The following studies present the original methodological descriptions: Normand *et al*. [54].

### Immunofluorescence and imaging

Cells were fixed using 4% paraformaldehyde (PFA) (Electron Microscopy Sciences) for 10 min at room temperature and then rinsed three times with PBS. Cells were permeabilized using 0.2% Triton X‐100 (catalog number T9284; Sigma‐Aldrich) for 10 min at room temperature before being rinsed twice with PBS. Cells were then incubated with primary antibodies (1 : 100 diluted in PBS‐4%BSA) for 40 min at room temperature, rinsed three times with PBS. The cells were then incubated with secondary antibodies (1 : 200 diluted in PBS‐4%BSA) for 30 min at room temperature. Nuclei were stained with DAPI (1 : 1000 dilution) and actin was stained with phalloidin (1 : 200 dilution). Coverslips were mounted on microscope slides using Fluoromount‐G mounting media (catalog number 0100‐01; SouthernBiotech, Birmingham, AL, USA) and were imaged using the SP5 confocal microscope (Leica Microsystems GmbH, Wetzlar, Germany). Collagen is observed by internal reflection microscopy (IRM). Images were analyzed using imagej (Bethesda, MD, USA) or fiji (Dresden, Germany) software.

The methods described here have also been detailed in previous publications. The following studies present the original methodological descriptions: Normand *et al*. [54].

### Live‐cell imaging

Cells were seeded on glass bottom dishes; then image acquisitions were performed every 2 min for 4 h using a LiveSR spinning‐disk microscope (Leica Microsystems GmbH) with environmental control at 37 °C and a CO_2_ provider. Z‐stacks were performed; then deconvolution imaging was used for 3D reconstruction (imaris software, Zurich, Switzerland).

### Correlative light and electron microscopy

For performing CLEM of invadosome rosettes, we did micropattern on ACLAR^®^ films (Catalog number 77850‐12; EMS, Hatfield, PA, USA) by laser microdissection [55]. A solution of gelatin (0.5 mg·mL^−1^) was precoated on these patterned ACLAR^®^ substrates in a 24‐well plate. The lifeact‐mRuby expressed NIH‐3T3‐Src cells were then seeded at 20 000 cells·mL^−1^ on ACLAR^®^ films and incubated overnight. The film was transferred to a 35 mm glass bottom dish (Catalog number P35G‐1.0‐20‐C; MatTek, Ashland, MA, USA) for confocal imaging (Leica SP2) using a ×63 objective (NA: 1.32; Leica Microsystems). The position of the region of interest (ROI) according to the landmarks visible in transmitted light. High‐magnification images of the cells and rosettes of interest were acquired as well as low‐magnification images. After imaging, cells were fixed in primary fixation buffer containing 2.5% PFA (Catalog number 15713; EMS) and 2.5% GA (Catalog number 16220; EMS) in 0.1 m cacodylate buffer (Catalog 11652; EMS) for 1 h at room temperature. Postfixation was performed in 1% osmium tetroxide (Catalog number 19150; EMS) in 0.1 m cacodylate buffer for 1 h on ice. Cells were stained with 2% uranyl acetate and then dehydrated in sequential gradient alcohol baths and infiltrated with Epon resin. We then inverted the film on top of the plastic embedding capsule (Catalog number 69910‐05; EMS) following overnight polymerization at 60 °C, thus embedding the intact cell monolayer of adhesion cells. The following day, the capsules were filled with fresh Epon and polymerized again overnight at 60 °C. Accurate positioning of the ROI can be relocated by a stereomicroscope according to the micropattern. Finally, the precisely trimmed resin block was done via serial sectioning (thickness: 70 nm) and all the sections were collected on slot grids for TEM observation. Micrographs were obtained at the transmitted electron microscope (CM12; FEI Company, Hillsboro, OR, USA) with a CCD camera (ORIUS; Gatan, Pleasanton, CA, USA). For the image processing, overlay the fluorescent and EM images in fiji and manually rescale or rotate the data until the cell boundaries align.

### Western blot

Cells were washed once with cold PBS on ice, and then were scraped with RIPA lysis buffer containing phosphatase and protease inhibitor cocktails (catalog number 04906845001; Merck, Burlington, MA, USA) and lysed for 30 min on ice. Cell lysates were clarified by centrifugation at 13 000 ***g*** for 15 min at 4 °C. Protein concentrations were determined using BCA reagent (DC™ Protein Assay; BioRad, Hercules, CA, USA), according to the manufacturer's protocol. Lysates were heated at 95 °C for 10 min and then separated by SDS/PAGE electrophoresis on 10% acrylamide gels (FastCast™ Acrylamide kit; BioRad) at 110 V for 60 min. Samples were transferred onto nitrocellulose membranes using a Transblot transfer system (BioRad) and membranes were blocked using 5%BSA with0.2%Tween (catalog number P7949; Sigma‐Aldrich) in TBS buffer (catalog number ET220; Euromedex, Soufffelweyersheim, France) for 1 h at room temperature before being incubated with primary antibodies (1 : 1000 dilutiond in blocking buffer as described above) overnight at 4 °C. The next day, membranes were washed three times with TBST and incubated with secondary antibodies (1 : 5000 dilution with 5% BSA in TBST) for 30 min at room temperature. Membranes were washed three times with TBST before exposure using a Chemidoc system (BioRad).

The methods described here have also been detailed in previous publications. The following studies present the original methodological descriptions: Normand *et al*. [54].

### Sunset assay

Cells were treated with puromycin (10 mg·mL^−1^) during 10 min at 37 °C, then washed twice in ice‐cold PBS for protein extraction as described above in the western blot section. For negative control, we pretreated cells with the translation inhibitor cycloheximide (35 mm) during 10 min at 37 °C.

### Immunoprecipitation

For immunoprecipitation experiments, 4.5 million cells were necessary and technical triplicates were performed. NIH‐3T3‐Src‐Tks5‐GFP and A431‐Tks5‐GFP cells were seeded on plastic or in dishes with a fibrillar collagen I matrix. Lysates were centrifuged for 15 min at 13 000 ***g*** at 4 °C, and supernatants were immunoprecipitated with the GFP‐Trap^®^_MA kit (Goldstandard) according to the supplier's recommendations. The immunoprecipitates were washed three times in lysis buffer and then assayed using the Lowry method (DC™ Protein Assay kit; BioRad) using a spectrophotometer at 750 nm.

### Mass spectrometry

The steps of sample preparation and protein digestion by the trypsin were performed as previously described [56]. NanoLC‐MS/MS analysis was performed using an Ultimate 3000 RSLC Nano‐UPHLC system (Thermo Scientific) coupled to a nanospray Q Exactive Hybrid Quadruple‐Orbitrap mass spectrometer (Thermo Scientific). Each peptide extract was loaded on a 300 μm ID × 5 mm PepMap C_18_ precolumn (Thermo Scientific) at a flow rate of 20 μL·min^−1^. After a 5 min desalting step, peptides were separated on a 75 μm ID × 25 cm C_18_ Acclaim PepMap® RSLC column (Thermo Scientific) with a 4–40% linear gradient of solvent B (0.1% formic acid in 80% ACN) in 108 min. The separation flow rate was set at 300 nL·min^−1^. The mass spectrometer operated in positive ion mode at a 1.8‐kV needle voltage. Data were acquired using xcalibur 3.1 software in a data‐dependent mode (Waltham, MA, USA). MS scans (*m/z* 350–1600) were recorded at a resolution of *R* = 70 000 (@ *m/z* 200) and an AGC target of 3 × 10^6^ ions collected within 100 ms. Dynamic exclusion of selected precursors was set to 30 s and top 12 ions were selected from fragmentation in HCD mode. MS/MS scans with a target value of 1 × 10^5^ ions were collected with a maximum fill time of 100 ms and a resolution of *R* = 17 500. Additionally, only +2 and +3 charged ions were selected for fragmentation. Other settings were as follows: no sheath and no auxiliary gas flow, heated capillary temperature, 200 °C; normalized HCD collision energy of 27% and an isolation width of 2 *m/z*. Protein identification and Label‐Free Quantification (LFQ) were done in proteome discoverer 3.0. chimerys node using prediction model inferys_2.1 fragmentation was used for protein identification in batch mode by searching against a Uniprot *Homo sapiens* database (82 439 entries, release March 2023) or a UniProt *Mus musculus* database (55 029 entries, release March 2023). Two missed enzyme cleavages were allowed for the trypsin. Peptide lengths of 7–30 amino acids, a maximum of three modifications, charges between 2 and 4, and 20 p.p.m. for fragment mass tolerance were set. Oxidation (M) and carbamidomethyl (C) were respectively searched as dynamic and static modifications by the chimerys software. Peptide validation was performed using the Percolator algorithm [57] and only “high confidence” peptides were retained corresponding to a 1% false discovery rate at the peptide level. Minora feature detector node (LFQ) was used along with the feature mapper and precursor ions quantifier. The normalization parameters were selected as follows: (a) Unique peptides, (b) Precursor abundance based on intensity, (c) No normalization was applied, (d) Protein abundance calculation: summed abundances, (e) Protein ratio calculation: pairwise ratio based, and (f) Missing values are replaced with random values sampled from the lower 5% of detected values. Quantitative data were considered for master proteins, quantified by a minimum of two unique peptides and a fold change ≥ 2.

The methods described here have also been detailed in previous publications. The following studies present the original methodological descriptions: Desigaux *et al*. [58].

### Inhibition of translation machinery present in invadosomes

A431‐Tks5‐GFP and NIH‐3T3‐Src‐Tks5‐GFP cells were seeded on coverslips coated with gelatin (dots/rosettes) or gelatin and type I collagen (linear invadosomes). The cells were treated with 35 μm of the translation inhibitor cycloheximide (CHX) (01810; Sigma‐Aldrich) or DMSO (control) (D8418; Sigma‐Aldrich) 2 h after seeding. For cells seeded on collagen, the cells were fixed 4 h after seeding. For cells seeded on gelatin, the cells were fixed 24 h after seeding by using 4% PFA. The immunofluorescence was performed as previously described. A total of 10 images were obtained for each condition using a confocal microscope (SP5; Leica) and experiments were done in three biological replicates. The number of invadosomes was quantified using imagej and normalized to the number of nuclei. For western blot analysis, cells were seeded on plates and treated with 35 μm of cycloheximide (CHX) or DMSO (control) 2 h after seeding. Before extraction, the cells were treated with Puromycin at a concentration of 10 μg·mL^−1^. Western blot was performed as previously described.

### Image analysis of fluorescence intensities and area of gelatin degradation

Sterile coverslips were coated with Oregon Green™ 488 conjugated gelatin 488 (catalog number G13186; Thermofisher) for 20 min and fixed with 0.5% glutaraldehyde (catalog number 15960; EMS) for 40 min. Then, the same experiment as described before was performed. Coverslips were imaged under the epifluorescence microscope (Zeiss, Oberkochen, Baden‐Wurttemberg, Germany) using the ×63 oil immersion objective. A total of 15 images were acquired for each condition, and experiments were done in three biological replicates. The area of degradation was quantified using imagej and normalized to the number of nuclei in each image.

### Statistical analysis

Data are reported as the mean ± SEM of at least three experiments. Statistical significance (*P* < 0.05 or less) was determined using analysis of variance (ANOVA) (one way, two way followed by a Bonferroni's correction), unpaired or paired Student's *t*‐test as appropriate, and performed with the graphpad prism (Boston, MA, USA) software. Significance levels are shown as *P*‐values.

## Conflict of interest

The authors declare no conflict of interest.

## Author contributions

LN, BB, and EH designed, performed experiments, and interpreted the data. MS performed experiments. VM interpreted the data. SDT, J‐WD, CD, and A‐AR performed and interpreted mass spectrometry analysis. LM and JGG contributed to CLEM analysis. LN, BB, VM, EH, and FS wrote and edited the manuscript. FS conceptualized the project.

## Supporting information


**Fig. S1.** Specific molecular proteins of each invadosomes organization.
**Fig. S2.** Characterization and validation of proteins identified by mass spectrometry as partner of Tks5.
**Fig. S3.** Translation is not involved on invadosome formation.
**Table S1.** Summary table of proteins present in invadosomes identified by mass spectrometry analysis of Tks 5 interactome.
**Table S2.** Summary table of proteins present in A 431 Tks 5 GFP cells seeded on plastic identified by mass spectrometry analysis of Tks 5 interactome.
**Table S3.** Summary table of proteins present in A 431 Tks 5 GFP cells seeded on collagen identified by mass spectrometry analysis of Tks 5 interactome.
**Table S4.** Summary table of proteins present in NIH 3 T 3 Src Tks 5 GFP cells seeded on plastic identified by mass spectrometry analysis of Tks 5 interactome.
**Table S5.** Summary table of proteins present in NIH 3 T 3 Src Tks 5 GFP cells seeded on collagen identified by mass spectrometry analysis of Tks 5 interactome.
**Table S6.** Summary table of the references used to classify the list of 88 common proteins as validated or not in invadosomes.

**Video S1.** KDEL‐GFP dynamics in lifeact‐mRuby (red)‐expressing NIH‐3T3‐Src.

**Video S2.** ER recruitment to invadosome rosette.

**Video S3a.** ER presence maintains invadosome rosette.

**Video S3b.** ER recruitment drop leads to invadosome collapse.

**Video S4.** CHX treatment promotes invadosome instability.

## Data Availability

All data supporting the findings of this study are available from the corresponding author upon reasonable request. The mass spectrometry proteomics data have been deposited to the ProteomeXchange Consortium via the PRIDE [59] partner repository. The mass spectrometry data are available Xchange with the identifier PXD046512 (NIH cells) and PXD046515 (A431 cells). Source data are provided with this paper.

## References

[1] Ferrari R , Martin G , Tagit O , Guichard A , Cambi A , Voituriez R , Vassilopoulos S & Chavrier P (2019) MT1‐MMP directs force‐producing proteolytic contacts that drive tumor cell invasion. Nat Commun 10, 4886. 31653854 10.1038/s41467-019-12930-y PMC6814785

[2] Watanabe A , Hosino D , Koshikawa N , Seiki M , Suzuki T & Ichikawa K (2013) Critical role of transient activity of MT1‐MMP for ECM degradation in invadopodia. PLoS Comput Biol 9, e1003086. 23737743 10.1371/journal.pcbi.1003086 PMC3667784

[3] Fekete A , Bőgel G , Pesti S , Péterfi Z , Geiszt M & Buday L (2013) EGF regulates tyrosine phosphorylation and membrane‐translocation of the scaffold protein Tks5. J Mol Signal 8, 8. 23924390 10.1186/1750-2187-8-8 PMC3765130

[4] Daubon T , Spuul P , Alonso F , Fremaux I & Génot E (2016) VEGF‐A stimulates podosome‐mediated collagen‐IV proteolysis in microvascular endothelial cells. J Cell Sci 129, 2586–2598. 27231093 10.1242/jcs.186585

[5] Varon C , Tatin F , Moreau V , Van Obberghen‐Schilling E , Fernandez‐Sauze S , Reuzeau E , Kramer I & Génot E (2006) Transforming growth factor β induces rosettes of podosomes in primary aortic endothelial cells. Mol Cell Biol 26, 3582–3594. 16611998 10.1128/MCB.26.9.3582-3594.2006 PMC1447430

[6] Jerrell RJ & Parekh A (2016) Matrix rigidity differentially regulates invadopodia activity through ROCK1 and ROCK2. Biomaterials 84, 119–129. 26826790 10.1016/j.biomaterials.2016.01.028 PMC4755854

[7] Parekh A , Ruppender NS , Branch KM , Sewell‐Loftin MK , Lin J , Boyer PD , Candiello JE , Merryman WD , Guelcher SA & Weaver AM (2011) Sensing and modulation of invadopodia across a wide range of rigidities. Biophys J 100, 573–582. 21281571 10.1016/j.bpj.2010.12.3733 PMC3030182

[8] Cannone S , Greco MR , Carvalho TMA , Guizouarn H , Soriani O , Di Molfetta D , Tomasini R , Zeeberg K , Reshkin SJ & Cardone RA (2022) Cancer associated fibroblast (CAF) regulation of PDAC parenchymal (CPC) and CSC phenotypes is modulated by ECM composition. Cancers (Basel) 14, 3737. 35954400 10.3390/cancers14153737 PMC9367491

[9] Juin A , Di Martino J , Leitinger B , Henriet E , Gary A‐S , Paysan L , Bomo J , Baffet G , Gauthier‐Rouvière C , Rosenbaum J *et al*. (2014) Discoidin domain receptor 1 controls linear invadosome formation via a Cdc42–tuba pathway. J Cell Biol 207, 517–533. 25422375 10.1083/jcb.201404079 PMC4242841

[10] Iizuka S , Leon RP , Gribbin KP , Zhang Y , Navarro J , Smith R , Devlin K , Wang LG , Gibbs SL , Korkola J *et al*. (2020) Crosstalk between invadopodia and the extracellular matrix. Eur J Cell Biol 99, 151122. 33070041 10.1016/j.ejcb.2020.151122

[11] Di Martino J , Henriet E , Ezzoukhry Z , Goetz JG , Moreau V & Saltel F (2016) The microenvironment controls invadosome plasticity. J Cell Sci 129, 1759–1768. 27029343 10.1242/jcs.182329

[12] Mueller SC , Ghersi G , Akiyama SK , Sang Q‐XA , Howard L , Pineiro‐Sanchez M , Nakahara H , Yeh Y & Chen W‐T (1999) A novel protease‐docking function of integrin at invadopodia. J Biol Chem 274, 24947–24952. 10455171 10.1074/jbc.274.35.24947

[13] Beaty BT & Condeelis J (2014) Digging a little deeper: the stages of invadopodium formation and maturation. Eur J Cell Biol 93, 438–444. 25113547 10.1016/j.ejcb.2014.07.003 PMC4262566

[14] Helgeson LA & Nolen BJ (2013) Mechanism of synergistic activation of Arp2/3 complex by cortactin and N‐WASP. Elife 2, e00884. 24015358 10.7554/eLife.00884 PMC3762189

[15] Di Martino J , Paysan L , Gest C , Lagrée V , Juin A , Saltel F & Moreau V (2014) Cdc42 and Tks5. Cell Adh Migr 8, 280–292. 24840388 10.4161/cam.28833 PMC4198353

[16] Seals DF , Azucena EF , Pass I , Tesfay L , Gordon R , Woodrow M , Resau JH & Courtneidge SA (2005) The adaptor protein Tks5/fish is required for podosome formation and function, and for the protease‐driven invasion of cancer cells. Cancer Cell 7, 155–165. 15710328 10.1016/j.ccr.2005.01.006

[17] Lock P , Abram CL , Gibson T & Courtneidge SA (1998) A new method for isolating tyrosine kinase substrates used to identify fish, an SH3 and PX domain‐containing protein, and Src substrate. EMBO J 17, 4346–4357. 9687503 10.1093/emboj/17.15.4346 PMC1170768

[18] Wang W , Zheng X , Azoitei A , John A , Zengerling F , Wezel F , Bolenz C & Günes C (2022) The role of TKS5 in chromosome stability and bladder cancer progression. Int J Mol Sci 23, 14283. 36430759 10.3390/ijms232214283 PMC9698602

[19] Moodley S , Hui Bai X , Kapus A , Yang B & Liu M (2015) XB130/Tks5 scaffold protein interaction regulates Src‐mediated cell proliferation and survival. Mol Biol Cell 26, 4492–4502. 26446840 10.1091/mbc.E15-07-0483 PMC4666142

[20] Buschman MD , Bromann PA , Cejudo‐Martin P , Wen F , Pass I & Courtneidge SA (2009) The novel adaptor protein Tks4 (SH3PXD2B) is required for functional podosome formation. Mol Biol Cell 20, 1302–1311. 19144821 10.1091/mbc.E08-09-0949 PMC2649273

[21] Ezzoukhry Z , Henriet E , Cordelières FP , Dupuy J‐W , Maître M , Gay N , Di‐Tommaso S , Mercier L , Goetz JG , Peter M *et al*. (2018) Combining laser capture microdissection and proteomics reveals an active translation machinery controlling invadosome formation. Nat Commun 9, 2031. 29795195 10.1038/s41467-018-04461-9 PMC5966458

[22] Stylli SS , Stacey TTI , Verhagen AM , Xu SS , Pass I , Courtneidge SA & Lock P (2009) Nck adaptor proteins link Tks5 to invadopodia actin regulation and ECM degradation. J Cell Sci 122, 2727–2740. 19596797 10.1242/jcs.046680 PMC2909319

[23] Iizuka S , Abdullah C , Buschman MD , Diaz B & Courtneidge SA (2016) The role of Tks adaptor proteins in invadopodia formation, growth and metastasis of melanoma. Oncotarget 7, 78473–78486. 27802184 10.18632/oncotarget.12954 PMC5346654

[24] Juin A , Billottet C , Moreau V , Destaing O , Albiges‐Rizo C , Rosenbaum J , Génot E & Saltel F (2012) Physiological type I collagen organization induces the formation of a novel class of linear invadosomes. Mol Biol Cell 23, 297–309. 22114353 10.1091/mbc.E11-07-0594 PMC3258174

[25] Thuault S , Mamelonet C , Salameh J , Ostacolo K , Chanez B , Salaün D , Baudelet E , Audebert S , Camoin L & Badache A (2020) A proximity‐labeling proteomic approach to investigate invadopodia molecular landscape in breast cancer cells. Sci Rep 10, 6787. 32321993 10.1038/s41598-020-63926-4 PMC7176661

[26] Zagryazhskaya‐Masson A , Monteiro P , Macé A‐S , Castagnino A , Ferrari R , Infante E , Duperray‐Susini A , Dingli F , Lanyi A , Loew D *et al*. (2020) Intersection of TKS5 and FGD1/CDC42 signaling cascades directs the formation of invadopodia. J Cell Biol 219, e201910132. 32673397 10.1083/jcb.201910132 PMC7480108

[27] Mader CC , Oser M , Magalhaes MAO , Bravo‐Cordero JJ , Condeelis J , Koleske AJ & Gil‐Henn H (2011) An EGFR‐Src‐Arg‐cortactin pathway mediates functional maturation of invadopodia and breast cancer cell invasion. Cancer Res 71, 1730–1741. 21257711 10.1158/0008-5472.CAN-10-1432 PMC3057139

[28] Wang Y , Qiu W , Chen J , Meng W , Zhao R , Lin W , Mei P , Diao M , Xiao H & Liao Y (2023) ERβ promoted invadopodia formation‐mediated non‐small cell lung cancer metastasis via the ICAM1/p‐Src/p‐Cortactin signaling pathway. Int J Cancer 153, 1287–1299. 37212571 10.1002/ijc.34563

[29] Tu C , Ortega‐Cava CF , Chen G , Fernandes ND , Cavallo‐Medved D , Sloane BF , Band V & Band H (2008) Lysosomal cathepsin B participates in the podosome‐mediated extracellular matrix degradation and invasion via secreted lysosomes in v‐Src fibroblasts. Cancer Res 68, 9147–9156. 19010886 10.1158/0008-5472.CAN-07-5127 PMC2764335

[30] Decotret LR , Wadsworth BJ , Li LV , Lim CJ , Bennewith KL & Pallen CJ (2021) Receptor‐type protein tyrosine phosphatase alpha (PTPα) mediates MMP14 localization and facilitates triple‐negative breast cancer cell invasion. Mol Biol Cell 32, 567–578. 33566639 10.1091/mbc.E20-01-0060 PMC8101463

[31] Inoue H , Kanda T , Hayashi G , Munenaga R , Yoshida M , Hasegawa K , Miyagawa T , Kurumada Y , Hasegawa J , Wada T *et al*. (2024) A MAP1B–cortactin–Tks5 axis regulates TNBC invasion and tumorigenesis. J Cell Biol 223, e202303102. 38353696 10.1083/jcb.202303102 PMC10866687

[32] Zhao P , Xu Y , Wei Y , Qiu Q , Chew T‐L , Kang Y & Cheng C (2016) The CD44s splice isoform is a central mediator for invadopodia activity. J Cell Sci 129, 1355–1365. 26869223 10.1242/jcs.171959 PMC6518308

[33] Challa S , Khulpateea BR , Nandu T , Camacho CV , Ryu KW , Chen H , Peng Y , Lea JS & Lee Kraus W (2021) Ribosome ADP‐Ribosylation inhibits translation and maintains proteostasis in cancers. Cell 184, 4531–4546.e26. 34314702 10.1016/j.cell.2021.07.005 PMC8380725

[34] Shahbazian D , Parsyan A , Petroulakis E , Topisirovic I , Martineau Y , Gibbs BF , Svitkin Y & Sonenberg N (2010) Control of cell survival and proliferation by mammalian eukaryotic initiation factor 4B. Mol Cell Biol 30, 1478–1485. 20086100 10.1128/MCB.01218-09 PMC2832492

[35] Mallawaaratchy DM , Buckland ME , McDonald KL , Li CCY , Ly L , Sykes EK , Christopherson RI & Kaufman KL (2015) Membrane proteome analysis of glioblastoma cell invasion. J Neuropathol Exp Neurol 74, 425–441. 25853691 10.1097/NEN.0000000000000187

[36] Pedersen NM , Wenzel EM , Wang L , Antoine S , Chavrier P , Stenmark H & Raiborg C (2020) Protrudin‐mediated ER–endosome contact sites promote MT1‐MMP exocytosis and cell invasion. J Cell Biol 219, e202003063. 32479595 10.1083/jcb.202003063 PMC7401796

[37] Ros M , Nguyen AT , Chia J , Le Tran S , Le Guezennec X , McDowall R , Vakhrushev S , Clausen H , Humphries MJ , Saltel F *et al*. (2020) ER‐resident oxidoreductases are glycosylated and trafficked to the cell surface to promote matrix degradation by tumour cells. Nat Cell Biol 22, 1371–1381. 33077910 10.1038/s41556-020-00590-w

[38] Fuentes LA , Marin Z , Tyson J , Baddeley D & Bewersdorf J (2023) The nanoscale organization of reticulon 4 shapes local endoplasmic reticulum structure in situ. J Cell Biol 222, e202301112. 37516910 10.1083/jcb.202301112 PMC10373298

[39] Hwang YS , Park K‐K , Cha IH , Kim J & Chung W‐Y (2012) Role of insulin‐like growth factor‐II mRNA‐binding protein‐3 in invadopodia formation and the growth of oral squamous cell carcinoma in athymic nude mice. Head Neck 34, 1329–1339. 22052854 10.1002/hed.21929

[40] Hwang YS , Xianglan Z , Park K‐K & Chung W‐Y (2012) Functional invadopodia formation through stabilization of the PDPN transcript by IMP‐3 and cancer‐stromal crosstalk for PDPN expression. Carcinogenesis 33, 2135–2146. 22859271 10.1093/carcin/bgs258

[41] Kim H‐Y , Ha Thi HT & Hong S (2018) IMP2 and IMP3 cooperate to promote the metastasis of triple‐negative breast cancer through destabilization of progesterone receptor. Cancer Lett 415, 30–39. 29217458 10.1016/j.canlet.2017.11.039

[42] Li D , Wang X , Miao H , Liu H , Pang M , Guo H , Ge M , Glass SE , Emmrich S , Ji S *et al*. (2023) The lncRNA MIR99AHG directs alternative splicing of SMARCA1 by PTBP1 to enable invadopodia formation in colorectal cancer cells. Sci Signal 16, eadh4210. 37725664 10.1126/scisignal.adh4210

[43] Chabadel A , Bañon‐Rodríguez I , Cluet D , Rudkin BB , Wehrle‐Haller B , Genot E , Jurdic P , Anton IM & Saltel F (2007) CD44 and β3 integrin organize two functionally distinct actin‐based domains in osteoclasts. Mol Biol Cell 18, 4899–4910. 17898081 10.1091/mbc.E07-04-0378 PMC2096584

[44] Magnuson B , Ekim B & Fingar DC (2011) Regulation and function of ribosomal protein S6 kinase (S6K) within mTOR signalling networks. Biochem J 441, 1–21. 10.1042/BJ20110892 22168436

[45] Costa DS , Kenny‐Ganzert IW , Chi Q , Park K , Kelley LC , Garde A , Matus DQ , Park J , Yogev S , Goldstein B *et al*. (2023) The *Caenorhabditis elegans* anchor cell transcriptome: ribosome biogenesis drives cell invasion through basement membrane. Development 150, dev201570. 37039075 10.1242/dev.201570 PMC10259517

[46] Kreider‐Letterman G , Castillo A , Mahlandt EK , Goedhart J , Rabino A , Goicoechea S & Garcia‐Mata R (2022) ARHGAP17 regulates the spatiotemporal activity of Cdc42 at invadopodia. J Cell Biol 222, e202207020. 36571786 10.1083/jcb.202207020 PMC9794838

[47] Badowski C , Pawlak G , Grichine A , Chabadel A , Oddou C , Jurdic P , Pfaff M , Albigès‐Rizo C & Block MR (2008) Paxillin phosphorylation controls invadopodia/podosomes spatiotemporal organization. Mol Biol Cell 19, 633–645. 18045996 10.1091/mbc.E06-01-0088 PMC2230606

[48] Zeng H , Huang J , Ren J , Wang CK , Tang Z , Zhou H , Zhou Y , Shi H , Aditham A , Sui X *et al*. (2023) Spatially resolved single‐cell translatomics at molecular resolution. Science 380, eadd3067. 37384709 10.1126/science.add3067 PMC11146668

[49] Cao L , Wang F , Li S , Wang X , Huang D & Jiang R (2019) PIM1 kinase promotes cell proliferation, metastasis and tumor growth of lung adenocarcinoma by potentiating the c‐MET signaling pathway. Cancer Lett 444, 116–126. 30583073 10.1016/j.canlet.2018.12.015

[50] Xu J , Lu Y , Liu Q , Xia A , Zhao J , Xu X , Sun Q , Qi F & Sun B (2020) Long noncoding RNA GMAN promotes hepatocellular carcinoma progression by interacting with eIF4B. Cancer Lett 473, 1–12. 31875526 10.1016/j.canlet.2019.12.032

[51] Liu T , Wei Q , Jin J , Luo Q , Liu Y , Yang Y , Cheng C , Li L , Pi J , Si Y *et al*. (2020) The m6A reader YTHDF1 promotes ovarian cancer progression via augmenting EIF3C translation. Nucleic Acids Res 48, 3816–3831. 31996915 10.1093/nar/gkaa048 PMC7144925

[52] Tang J , Long G , Li X , Zhou L , Zhou Y & Wu Z (2023) The deubiquitinase EIF3H promotes hepatocellular carcinoma progression by stabilizing OGT and inhibiting ferroptosis. Cell Commun Signal 21, 198. 37559097 10.1186/s12964-023-01220-2 PMC10413709

[53] Yu W , Liang J , Fang T , Jiang J , Zhao R , Li R , Han J & Tian H (2023) EIF4A3 acts on the PI3K–AKT–ERK1/2–P70S6K pathway through FLOT1 to influence the development of lung adenocarcinoma. Mol Cancer Res 21, 713–725. 37011005 10.1158/1541-7786.MCR-22-0984 PMC10320473

[54] Normand L , Rouyer L , Richard E , Allain N , Giraud J , Di‐Tommaso S , Dourthe C , Raymond A‐A , Dupuy J‐W , Moreau K *et al*. (2025) Cancer cells transfer invasive properties through microRNAs contained in collagen‐tracks. *bioRxiv*. doi: 10.1101/2025.01.25.634857 41379613

[55] Spiegelhalter C , Laporte JF & Schwab Y (2014) Correlative light and electron microscopy: from live cell dynamic to 3D ultrastructure. Methods Mol Biol 1117, 485–501. 24357376 10.1007/978-1-62703-776-1_21

[56] Henriet E , Abou Hammoud A , Dupuy J‐W , Dartigues B , Ezzoukry Z , Dugot‐Senant N , Leste‐Lasserre T , Pallares‐Lupon N , Nikolski M , Le Bail B *et al*. (2017) Argininosuccinate synthase 1 (ASS1): a marker of unclassified hepatocellular adenoma and high bleeding risk. Hepatology 66, 2016–2028. 28646562 10.1002/hep.29336

[57] Käll L , Canterbury JD , Weston J , Noble WS & MacCoss MJ (2007) Semi‐supervised learning for peptide identification from shotgun proteomics datasets. Nat Methods 4, 923–925. 17952086 10.1038/nmeth1113

[58] Desigaux T , Comperat L , Dusserre N , Stachowicz M‐L , Lea M , Dupuy J‐W , Vial A , Molinari M , Fricain J‐C , Paris F *et al*. (2024) 3D bioprinted breast cancer model reveals stroma‐mediated modulation of extracellular matrix and radiosensitivity. Bioact Mater 42, 316–327. 39290339 10.1016/j.bioactmat.2024.08.037 PMC11405629

[59] Deutsch EW , Bandeira N , Perez‐Riverol Y , Sharma V , Carver JJ , Mendoza L , Kundu DJ , Wang S , Bandla C , Kamatchinathan S *et al*. (2022) The ProteomeXchange consortium at 10 years: 2023 update. Nucleic Acids Res 51, D1539–D1548. 10.1093/nar/gkac1040 PMC9825490 36370099

