# Supplementary material for: Tks5 interactome reveals endoplasmic‐reticulum‐associated translation machinery in invadosomes
Source: FEBS J. 2025 Jul 24;292(23):6400–19. doi: 10.1111/febs.70196 (PMC12699180; doi:10.1111/febs.70196)
Supplement: Supplementary file 1 — Fig. S1. Specific molecular proteins of each invadosomes organization. Fig. S2. Characterization and validation of proteins identified by mass spectrometry as partner of Tks5. Fig. S3. Translation is not involved on invadosome formation. Table S1. Summary table of proteins present in invadosomes identified by mass spectrometry analysis of Tks 5 interactome. Table S2. Summary table of proteins present in A 431 Tks 5 GFP cells seeded on plastic identified by mass spectrometry analysis of Tks 5 interactome. Table S3. Summary table of proteins present in A 431 Tks 5 GFP cells seeded on collagen identified by mass spectrometry analysis of Tks 5 interactome. Table S4. Summary table of proteins present in NIH 3 T 3 Src Tks 5 GFP cells seeded on plastic identified by mass spectrometry analysis of Tks 5 interactome. Table S5. Summary table of proteins present in NIH 3 T 3 Src Tks 5 GFP cells seeded on collagen identified by mass spectrometry analysis of Tks 5 interactome. Table S6. Summary table of the references used to classify the list of 88 common proteins as validated or not in invadosomes. [file FEBS-292-6400-s006.zip › combined Fig sup tables.pdf]

Supp Figure 1: Specific molecular signature of each invadosomes organizations.

a

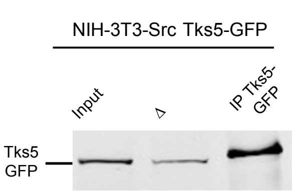

b

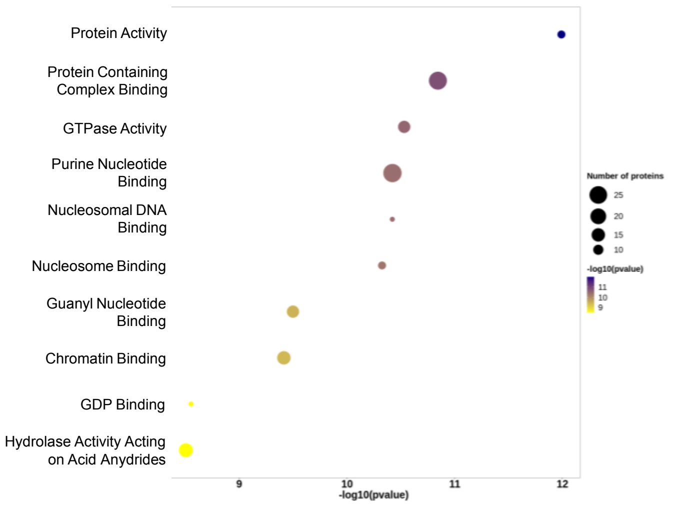

c List of proteins present in rosettes

**Translation proteins (3%)**	EIF1A, EIF3S3X, EIF5A, MRPL12, NGDN
**Mitochondrial proteins (8%)**	AFG3L1, ATAD1, ATP5PB, MRPL12, NDUFV1, NFU1, PGAM5, RDH13, SLC25A4, SLC25A10, SLC29A1, SLC44A2
**Endoplasmic reticulum proteins (6%)**	CISD2, CNPY2, RAB1A, RAB1B, SEC22B, TMED9, TMX1, UBXN4
**Proteins of the golgi apparatus (4%)**	RAB1A, RAB1B, RAB6A, SEC22B, TMED9, TMED10
**Microtubule proteins**	STMN1

d

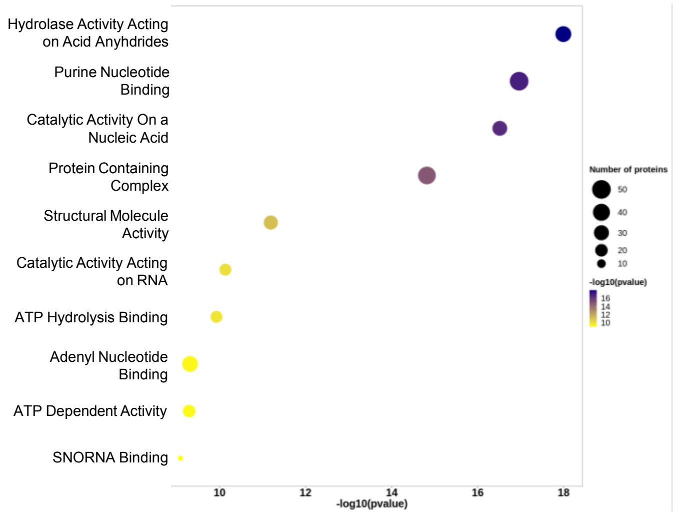

e List of proteins present in dots

**Translation proteins (9%)**	BOP1, BRIX1, DDX27, EEFSEC, EIF3CL, EIF3I, EIF6, GNL2, IMP4, LARP4B, MRM3, MRPL2, MRPL15, MRPL37, MRPL47, MRPS12, MRPS15, MRPS23, MRPS34, NOP56, NSUN4, POLDIP3, RPS15, YTHDF1
**Mitochondrial proteins (12%)**	CHCHD3, CYC1, DIABLO, DRG1, GTF3C4, HK2, HTRA2, IMMT, MCAT, MRM3, MRPL2, MRPL15, MRPL37, MRPL47, MRPS12, MRPS15, MRPS23, MRPS34, NDUFA10, NDUFS2, NIPSNAP1, NSUN4, PRORP, PTPMT1, RAB38, RHOT1, SFXN3, SLC25A3B16, TEFM, VDAC1, VDAC2
**Endoplasmic reticulum proteins (9%)**	DHCR7, DNAJB11, DNAJC10, DPM1, EMC3, ERLIN2, HTRA2, PIGT, PRKAR2A, PUM3, RCN2, SEC61B, SEC62, SET, SLC35B2, SRP72, STT3A, SURF4, TAP2, TECR, TMCC1, TMEM214, TMX3, TOR1AIP2, UFL1
**Proteins of the golgi apparatus (3%)**	MGAT1, MYO18A, NSF, PRKAR2A, RAB34, SLC35B2, SURF4, TMEM214
**Microtubule proteins (2%)**	MARK3, TBCA, TMEM214, TTLL3, TUBB, TUBB4B

f

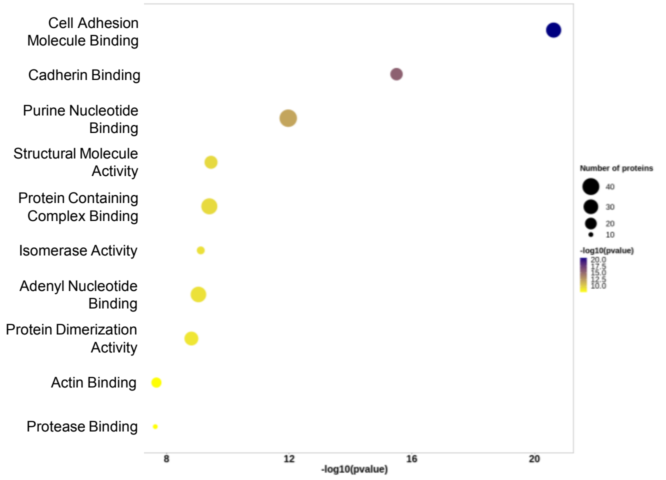

**Adhesion proteins (3%)**	CCN1, CD34, CD151, DAG1, ECM1, FN1, ITGB1, TSPAN6

g List of proteins present in linear invadosomes

**Translation proteins (5%)**	DHX37, EEF1D, EIF2B4, EIF3J2, EIF3L, EIF5, EIF5B, ERAL1, MRPL44, PTCD1, PUM1, PYM1, RPS21, RWDD1
**Mitochondrial proteins (9%)**	ABCB7, ACADL, AFG3L2, AK4, AK4P3, ATP5MF, CLPX, DHODH, ECHS1, ERAL1, ETFA, ETFB, ETHE1, GFM1, GLUD1, HADH, HMGCL, HSPD1, IDH2, MFN1, MIX23, MRPL44, NDUFA4, PTCD1, PYCR1, TOMM70, YARS2
**Endoplasmic reticulum proteins (6%)**	ABHD12, ATL2, ATXN2, COPB1, CRELD2, ERAP1, ERLEC1, FKBP9, GOLT1B, HM13, LCLAT1, PIGK, PIGS, PLP2, SACM1L, SEC23IP, SSR1, UFD1
**Proteins of the golgi apparatus (5%)**	ARFGAP1, ARFIP1, COPB1, CRELD2, FAM20B, GOLGA5, GOLIM4, NSFL1C, STX6, TGOLN2, TMEM87A, USO1, VTI1A, YIPF3
**Microtubule proteins (1%)**	CEP170, DYNC1H1, MAP1B, MAP6, TACC2

Supp Figure 2 : Characterization and validation of proteins identified by mass spectrometry as partner of Tks5.

a

Proteins	A431 Gelatin	A431 Collagen	NIH-3T3-Src Gelatin	NIH-3T3-Src Collagen
ACTB	X	X		
CRKL	X			
CTTN	X	X	X	X
DBN1			X	X
DDX3X	X	X		
EEF2	X	X		
G3BP1	X	X	X	X
GRB2	X		X	
HNRNPK	X	X		
HSP90B1		X		
MARS1	X	X		
MTHFD1	X	X		
NDRG1	X	X		
NCL	X	X		
PRKAA1	X	X	X	X
PRKAG1	X	X	X	X
RUVBL1	X			
RUVBL2	X			

Stylli et *al.*, 2009

Proteins	A431 Gelatin	A431 Collagen	NIH-3T3-Src Gelatin	NIH-3T3-Src Collagen
ADAM15	X	X	X	X
ARAG	X	X		
CD2AP				X
CTTN	X	X	X	X
DBNL			X	X
EIF2S3	X	X		
EIF4A3	X	X	X	
FGD1	X	X	X	
FNBP1	X	X	X	X
HSPA1B	X	X		
IGF2BP2	X	X	X	X
MAP4	X	X	X	X
NAP1L1	X	X		
NUDT5	X			
PDLIM1				X
PRDX4	X			
RPIA				X
RTN4	X	X	X	X
SH3PXD2A	X	X	X	X
TJP1	X	X		
USP15	X	X		
WASL	X	X		

Thuault et *al.*, 2020

Proteins	A431 Gelatin	A431 Collagen	NIH-3T3-Src Gelatin	NIH-3T3-Src Collagen
RPS7	X		X	
RPS8	X	X	X	X
RPS2	X	X		X
RPS4	X	X	X	X
RPS9	X	X		
RPL12	X	X		X
RPL7	X	X	X	X
RPL3	X	X	X	X
LARP4	X	X	X	X
PP13296				
RPL11	X	X		
RPS11	X	X		X
RPSA	X	X		
RPL24	X	X		
PABPC1	X	X	X	X
RPL6	X	X	X	X
RPS3A	X	X		X
FAU	X	X		X
RPL23A	X		X	
RPS7	X		X	
RPS8	X	X	X	X
RPS2	X	X		X

Zagryazhskaya-Masson et *al.*, 2020

b

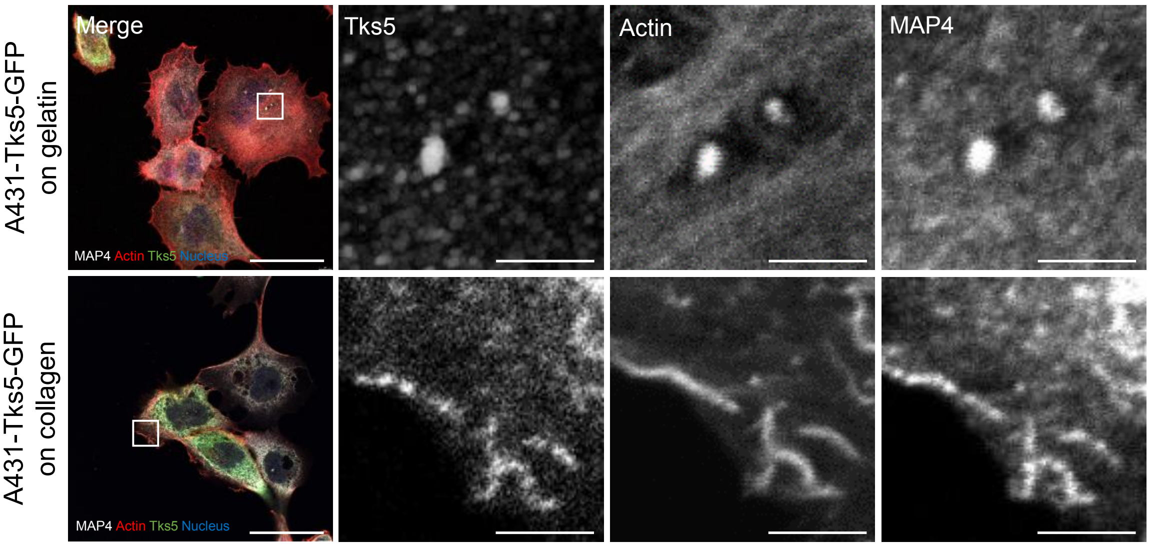

c

**Translation**	BZW2, EIF3A, EIF4B, FXR1, G3BP1, HNRNPA1, HNRNPAB, IGF2BP2, LARP4, NONO, NUFIP2, PABPC1, PTBP1, PUF60, RPL0, RPL3, RPL4, RPL5, RPL6, RPL7, RPL7A, RPL8, RPL10A, RPL13, RPL14, RPL15, RPL18A, RPL19, RPL29, RPL32, RPL34, RPL36, RPS4X, RPS6, RPS6KA4, RPS8, RRBP1, RTRAF, SPATS2, SRSF1, SSB, STAU1, SYNCRIP, TCOF1, YBX1, YBX3
**Actin Cytoskeleton**	CALD1, CTTN, EMD, FNBP1L, MYH9,
**Adhesion**	CD44, ITGA5, PVR
**Others**	ADAM15, ADAM19, ASPH, BAG3, BASP1, C1QBP, CLTB, DLD, GUSB, HNRNPU, LAMP1, LAMP2, LRRC59, MAP4, MMP14, NPM1, PPM1G, PPP1CA, PRKAA1, PRKAG1, PRR2C2, PRRC2A, PSMD7, RTN4, SART1, STX7, THBD, YWHAB, YWHAE, YWHAG, YWHAH, YWHAQ, YWHAZ

d

**Stylli et *al*, 2009**	DDX3X, EEF2, HNRNPK, MetRS, NCL
**Thuault et *al*, 2020**	EIF2S3, EIF4A3, ELP4, ELP6, EPL5, IGF2BP2, MREAA1
**Mallawaaratchy et *al*, 2015**	EEIF1E1, KARS1, YBX1
**Ezzoukhry et *al*, 2018**	ANP32A, BTF3L4, CARS, DDX3X, DDX17, EEF1A2, EEF1D, EEF1G, EEF2, EEFA1A, EFTUD2, EIF2A, EIF2S1, EIF3C, EIF3E, EIF3F, EIF3G, EIF3H, EIF3I, EIF3K, EIF3L, EIF3M, EIF4B, EIF5A, ELAVL1, EPRS, FARSA, FXR1, G3BP1, HNNRPF, HNRNPA1, HNRNPA2B, HNRNPC, HNRNPH1, HNRNPK, HNRNPL, HNRNPM, HSP90B1, HSPA5, IGF2BP1, IGF2BP2, IGF2BP3, KHSRP, MYBBPA1, NONO, PA2G4, PABPC1, PLR35A-PS3, PSMC1, PTBP1, RARS, RBM14, RPL10, RPL10A, RPL12, RPL22, RPL23, RPL23-PS3, RPL26, RPL28, RPL30, RPL31, RPL34, RPL35A, RPL36A, RPL37A, RPS3, RPS4X, RPS11, RPS13, RPS14, RPS15A, RPS16, RPS20, RPS26, RPSA, RRBP1, RSP3A, RSP12, RSP19, SERBP1, SF3A1, SF3B3, SNRPA, SRRT, SYNCRIP, TRIM28, YBX3
**Zagryazhskaya-Masson et *al*, 2020**	FAU LARP4 PABPC1 RPL3 RPL6 RPL7 RPL11 RPL12 RPL23A RPL24 RPS2 RPS3A RPS4 RPS7 RPS8 RPS9 RPS11 RPSA

e

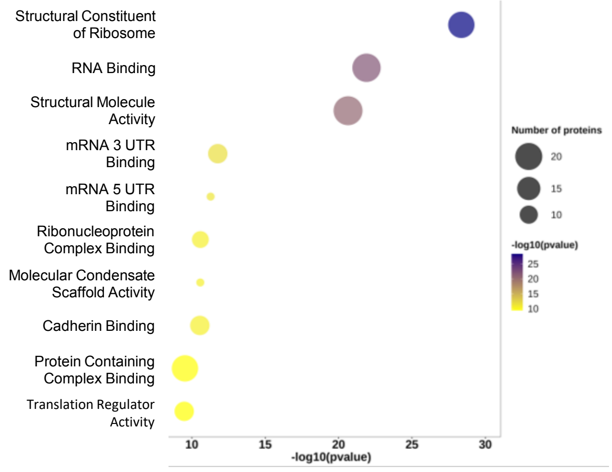

f

Proteins commonly identified in rosettes in Tks5 immunoprecipitation experiment and Ezzoukhry et al. paper
ANP32A, ARPC4, BAG3, CALD1, CAMK2D, CAPRIN1, CHMP4B, CKAP4, CLTA, CTTN, DDX17, DLST, EIF2S1, EIF3C, EIF3H, EIF4B, EIF5A, ELAVL1, FLNB, FUBP1, FXR1, G3BP1, GRPEL1, HNRNPA1, HNRNPH1, HNRNPM, HSPE1, IGF2BP1, IGF2BP2, IGF2BP3, KPNA2, LASP1, MAP4, MYBBP1A, MYEF2, MYH9, MYL6, MYOF, NACA, NONO, NPM1, PABPC1, PABPC4, PPP1CA, PRDX3, PSMA2, PTBP1, PURB, RAB5C, RAB14, RAB21, RPL10A, RPL12, RPL22, RPL23A, RPL28, RPL30, RPL34, RPL35A, RPL37A, RPS3A, RPS4X, RPS11, RPS12, RPS14, RPS15A, RPS19, RPS20, RPS26, RPS27, RRBP1, SFPQ, SNRPA, SRRT, STMN1, SYNCRIP, TARDBP, YBX3

Translation proteins highlighted in red in the table

Supp Figure 3: No effect of translation on invadosomes formation.

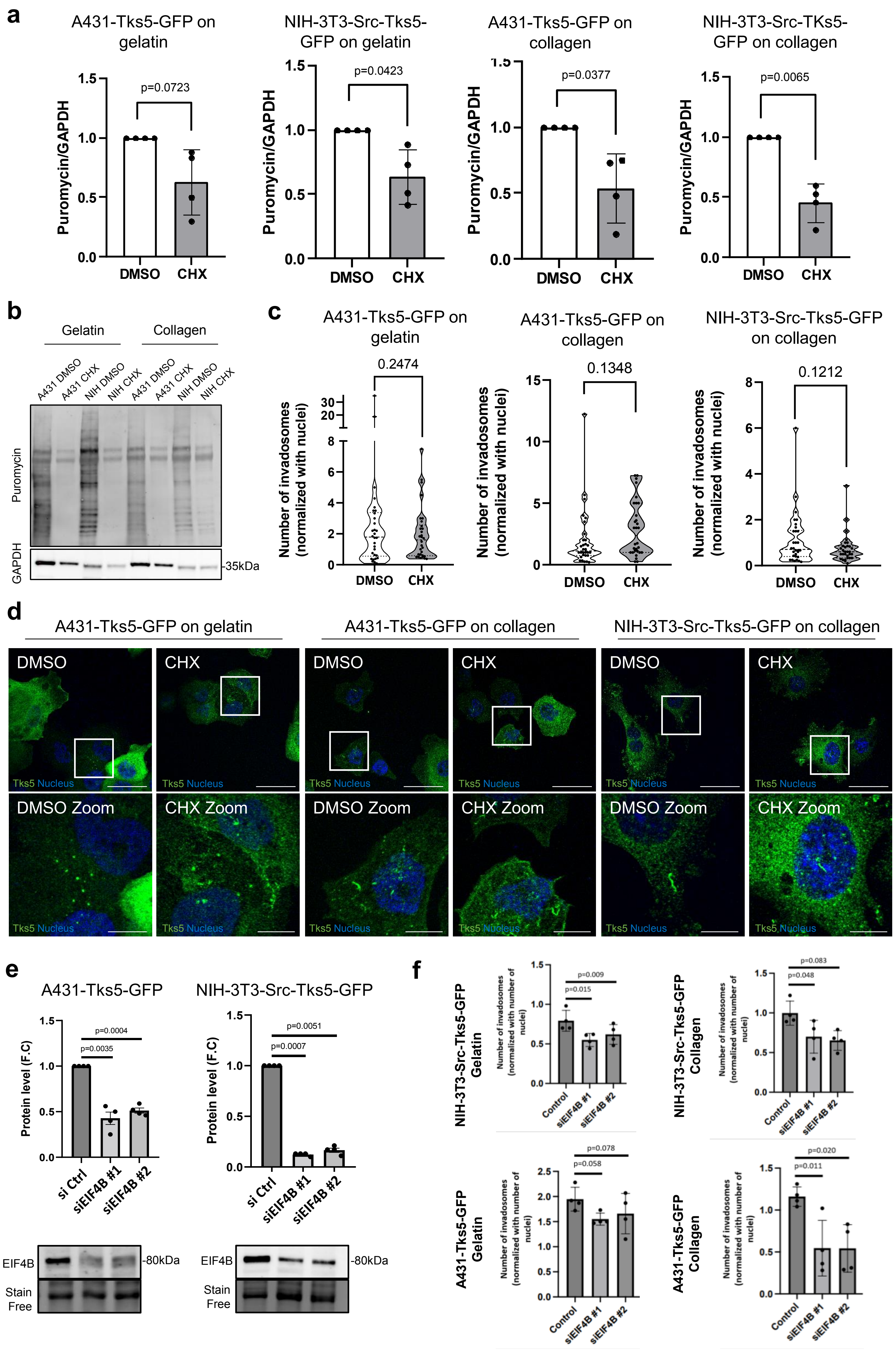

Supplemental table 1 : Summary table of proteins present in invadosomes identified by mass spectrometry analysis of Tks5 interactome

Names	Total	Elements
A431_C A431_P NIH3T3_C NIH3T3_P	88	ADAM15 ADAM19 ASPH BAG3 BASP1 BZW2 C1QBP CALD1 CD44 CLTB CTTN DLD EIF3A EIF4B EMD FNBP1L FXR1 G3BP1 GUSB HNRNPA1 HNRNPAB HNRNPU IGF2BP2 ITGA5 LAMP1 LAMP2 LARP4 LRRC59 MAP4 MMP14 MYH9 NONO NPM1 NUFIP2 PABPC1 PPM1G PPP1CA PRKAA1 PRKAG1 PRRC2A PRRC2C PSMD7 PTBP1 PUF60 PVR RPL3 RPL4 RPL5 RPL6 RPL7 RPL7A RPL8 RPL10A RPL13 RPL14 RPL15 RPL18A RPL19 RPL29 RPL32 RPL34 RPL36 RPLP0 RPS4X RPS6 RPS6KA4 RPS8 RRBP1 RTN4 RTRAF SART1 SH3PXD2A SPATS2 SRSF1 SSB STAU1 STX7 SYNCRIP TCOF1 THBD YBX1 YBX3 YWHAB YWHAE YWHAG YWHAH YWHAQ YWHAZ
A431_P NIH3T3_C NIH3T3_P	29	ALYREF BCAP31 CAV2 CCDC47 CD9 CHMP4B DLST DNAJC5 FUBP1 FUS GOLM1 HPRT1 ITGA3 LASP1 MANF NACA NHERF1 PCNP PPP1CB PPP1R12A PRDX3 PRKCSH RCN1 RCN3 RPS17 SNAP23 SOD2 SRSF5 ZRANB2
A431_C NIH3T3_C NIH3T3_P	6	CLTA EEA1 EIF3C P4HB SMNDC1 SRSF2
A431_C A431_P NIH3T3_P	84	ACSL4 AIFM1 AP2A1 AP2A2 AP2B1 AP2M1 ARHGEF40 CAPRIN1 CCDC86 CD47 CELF1 CKAP4 CSDE1 DARS1 DDX1 DDX5 DDX17 DHX15 EBNA1BP2 EIF2S1 EIF3H EIF4A3 EIF4E EXOSC9 FAU FBXO3 FGD1 FLNB FXR2 GTPBP4 H1-0 HNRNPA0 HNRNPH1 HNRNPM HNRNPUL1 IGF2BP3 ILF2 KHDRBS1 KRT6A LARP1 LARS1 MIB1 MOGS MRTO4 MYL6 MYO1E MYOF NCBP1 NIPSNAP2 NSDHL PABPC4 PAK1IP1 PCDH1 PRKAB1 PRKRA PURA PURB QARS1 RBM17 RCC2 RFC4 RPL12 RPL22 RPL28 RPL30 RPL35A RPL37A RPS3A RPS11 RPS12 RPS19 RPS26 RPS27 SEC63 SFPQ SLC25A13 SMC4 SNRPA1 SQOR TARDBP TMOD3 U2AF2 UPF1 ZC3HAV1
A431_C A431_P NIH3T3_C	36	ACOT9 ATXN2L BSG CALR CCT2 CDK1 CS CYB5R3 CYCS EIF4G1 ERO1A H1-5 HNRNPA2B1 HSD17B10 HSD17B12 HYOU1 KTN1 LGALS3 LUZP1 MAP7D1 MSN MTDH NPTN RAB11FIP1 RPL18 RPS2 SEPTIN2 SEPTIN9 SERPINH1 SF3B2 SH3KBP1 TFG TOR1AIP1 TPM1 TTC1 ZC3H15
NIH3T3_C NIH3T3_P	92	ACIN1 ACP2 ALCAM ANP32A APOBR ARGLU1 ARID1A ARPC4 BCLAF1 BIN1 BRI3BP BTF3 CAST CCDC9 CCDC124 CD63 CD81 CDH2 COLEC12 CSTF2 DBN1 DBNL DDX46 DEK DNAJC8 EEF1B EIF4H ELOB EMB EPB41L2 FKBP3 FKBP10 FXYD5 GM45713 GSR GTF2F1 H1-2 HDGF HDGFL2 HMBS HSPE1 IGF2BP1 IWS1 LGALS1 LRPAP1 MAGEA13 MAP1A MARCKS MXRA7 NES NOLC1 NPC2 NUCB1 NUCKS1 NUP50 PACSIN2 PAKAP PEX19 PGRMC1 PGRMC2 PHACTR4 PLIN3 PMF1 PPP1R18 PPP4R2 PTGES RAB11FIP5 REEP5 RHOC RPL10L RPL17 RTN3 S100A11 SARNP SGTA SIRPA SLC2A1 SNAP29 SOD1 SPARC SRSF7 THRAP3 TP53BP1 TPBG TPR TSPAN3 TXN UBAP2 UBAP2L UBQLN4 VTI1B WASHC2
A431_P NIH3T3_P	32	CDK11B CFL1 CSNK2B DDX56 DKC1 ELAVL1 G3BP2 GRB2 GRPEL1 H1-4 HP1BP3 LEMD2 NAT10 NOC4L NUDT21 OCIAD1 PRC1 PSMB2 RAB10 RAB21 RALB RPL23A RPLP2 RPS5 RPS7 RPS14 RPS20 RPS28 RRP1 RTCA SF1 UTP14A
A431_C NIH3T3_P	11	ESYT2 FMR1 GOT2 KRT16 MAN2A1 PDCD10 PSME2 RPS15A SNRPB2 SQSTM1 SRRT
A431_P NIH3T3_C	31	CALU CAPZB CDV3 CLINT1 CNPY3 DDRGK1 EFHD2 EWSR1 HNRNPA3 LNPEP MAPRE1 MYH10 NUCB2 NUDC PAWR PEX14 PFN1 PSAP PSME1 RAD23B RANGAP1 RPS24 SCAMP1 SERBP1 SERPINC1 SLC44A1 SNX1 STX4 STX12 TPM4 TWF1
A431_C NIH3T3_C	10	ARFGAP1 COL1A1 ECE1 FAM177A1 HADH KRT1 PCYOX1 PDLIM5 PLXNB2 SCP2
A431_C A431_P	587	AAAS ABCF1 ABHD10 ABI1 ABO ACAD9 ACADVL ACBD3 ACOT8 ACSL3 ACTBL2 ACTG1 ACTN1 ACTN4 ACTR2 ACTR3 ADAM10 ADAMTSL4 ADAR ADPGK AFAP1L2 AGK AHCYL1 AIFM2 AIMP1 AIMP2 AK2 AKAP12 ALDH1A3 ALDH3A2 AMY1A ANXA1 APMAP ARAF ARCN1 ARHGAP1 ARHGEF2 ARL6IP5 ARPC2 ASAH1 ASCC1 ASCC2 ASCC3 ATAD3A ATL3 ATP1A1 ATP2A2 ATP2B4 ATP5F1A ATP5F1C ATXN10 BAG4 BAIAP2L1 BCL2L13 BUB3 BYSL C1orf226 C6orf132 C7orf50 C8orf33 CAD CAND1 CANX CAPN1 CAPZA1 CAPZA2 CAVIN1 CAVIN3 CBLB CCNK CCT3 CCT4 CCT6A CCT7 CCT8 CD82 CDC5L CDC42EP1 CDCP1 CDH1 CDH3 CDK9 CDK13 CENPB CEP170B CGAS CHCHD2 CKAP5 CKMT1A CLP1 CLTC CMAS CNP COL17A1 COLGALT1 COPA COPB2 COPE COPG1 CORO1C CPNE1 CPOX CPSF6 CPSF7 CPT1A CRYBG1 CSNK1A1 CSNK2A1 CTNNA1 CTNNB1 CTNND1 CTPS1 CTSB CTSD CUL7 CYB5R1 CYFIP1 CYP2S1 DAP3 DAPK3 DCAF5 DDB1 DDOST DDX3X DDX6 DDX21 DDX24 DDX39B DDX47 DDX55 DDX60 DECR1 DHCR24 DHRS7 DHX9 DHX30 DHX36 DHX57 DIMT1 DNAJA2 DNAJA3 DNAJB12 DNAJC13 DTX3L ECH1 EDC4 EEF2 EGFR EHD2 EHD4 EIF2AK2 EIF2S2 EIF2S3 EIF3B EIF3E EIF3F EIF3G EIF3J EIF4A1 EIF4A2 EIF4G2 ELAVL2 EMC1 EMC2 EPHA2 EPRS1 ERBIN ERGIC1 ERGIC3 ERMP1 ERP29 ERP44 ESRP1 ESYT1 EXOSC2 EXOSC5 EXOSC10 F11R FAF2 FAM3C FAM83A FAM91A1 FAM98A FAM98B FAM120A FAR1 FBXO22 FH FHL2 FHL3 FIP1L1 FLII FLNA FLOT1 FNBP1 FNDC3B GAA GALNT2 GALNT3 GANAB GBA1 GEMIN4 GET3 GIPC1 GLG1 GLS GMPS GNL3 GNL3L GOLGA2 GOLGA4 GPD2 GRB7 GRSF1 GRWD1 GSN GTF3C2 H1-10 HACD3 HADHA HADHB HARS1 HDAC6 HECTD1 HELZ2 HLA-A HLA-B HMOX2 HNRNPC HNRNPD HNRNPF HNRNPK HNRNPR HNRNPUL2 HSD17B4 HSDL2 HSP90B1 HSPA1B HSPA2 HSPA5 HSPA8 HSPB1 HSPH1 IARS1 ICAM1 IDH3B IFI16 IKBIP ILF3 INF2 INPP5K IPO5 IQGAP1 ISG20L2 ITCH ITGA2 ITGAV ITGB6 IVL KARS1 KCTD12 KIAA0930 KIAA1522 KIF2A KPNB1 KRT6B KRT8 KRT17 KRT18 L1RE1 LACTB LAMA3 LAMB3 LAMC2 LANCL2 LCN2 LETM1 LGALS3BP LIMA1 LMAN1 LMAN2 LPCAT2 LRPPRC LRRC1 LRRC8A LSG1 LSR LUC7L2 MACF1 MAGEA4 MAGED2 MAN1B1 MAOA MAP7 MARK2 MARS1 MAVS MBOAT7 MCM5 MDH2 MFAP1 MICAL2 MIOS MLEC MORC2 MOV10 MRPL4 MRPS2 MRPS5 MRPS7 MRPS9 MRPS18B MRPS22 MRPS27 MRPS31 MRPS35 MSH2 MSH6 MTHFD1L MTHFD2 MTPAP MTREX MVP MX1 MYCBP2 MYO1B MYO1C MYO1D MYO5A MYO5B MYO6 NAGK NAP1L1 NCEH1 NCKAP1 NCL NCLN NCSTN NDRG1 NECTIN4 NIT2 NKRF NMNAT1 NOMO1 NOP2 NRDC NSUN2 OAS2 OAS3 OASL OAT OPA1 OSBPL8 OTUB1 P4HA2 PA2G4 PACSIN3 PARP1 PARP9 PARP12 PARS2 PBXIP1 PC PCBP2 PCYT1A PDIA3 PDIA4 PDIA5 PES1 PFKM PFKP PHLDA1 PIP5K1A PISD PKP3 PLBD2 PLEKHA5 PLOD1 PLOD2 PLOD3 PLRG1 PLSCR1 PML POLRMT PON2 POP1 POR PPFIA1 PPFIBP1 PPIB PPP2CA PPP2R1A PRKAB2 PRKAG2 PRKDC PRMT1 PRMT5 PRPF3 PRPF19 PSMA4 PSMC2 PSMC5 PSMD2 PSMD3 PSMD8 PSMD11 PTCD3 PTDSS1 PTGES2 PTGS1 PTPN1 PTPN6 PWP1 QSOX1 RAB3GAP1 RAB3GAP2 RACK1 RAE1 RARS1 RASAL2 RBM4 RBM14 RBM28 RBM39 RCL1 RDH11 RECQL RETSAT RFC2 RFC3 RFC5 RHOT2 RIOX2 RNH1 RPL9 RPL10 RPL11 RPL13A RPL17-C18orf32 RPL21 RPL23 RPL24 RPL26 RPL27 RPL27A RPL31 RPL35 RPN1 RPN2 RPS3 RPS9 RPS13 RPS16 RPS18 RPS23 RPS25 RPSA RTCB SART3 SCAMP2 SCARB2 SCFD1 SCRIB SDC4 SDHA SEC16A SEC23A SEC23B SEC24C SEC61A1 SEMA3C SERPINB1 SERPINB2 SERPINE1 SFN SGPL1 SH3BP4 SHMT2 SLC3A2 SLC7A5 SLC16A3 SLC25A22 SLC25A24 SLC27A4 SLFN5 SMAD3 SMC1A SND1 SNX9 SPATS2L SPINT1 SPOCD1 SPTAN1 SPTBN1 SPTLC1 SPTLC2 SRP54 SRP68 SRPK1 SRPRA SRPRB SRRM2 STAU2 STIM1 STOM STRAP SUMF2 SUPT16H SVIL TACSTD2 TAP1 TAPBP TARS2 TBL2 TCP1 TFAM TFB1M TFB2M TFRC TGFBI THBS1 THSD4 TIMM44 TIMM50 TJP1 TM9SF2 TM9SF4 TMCC3 TMEM43 TMPO TMTC3 TNFAIP2 TNKS1BP1 TOE1 TOP1 TOR4A TRABD TRAF7 TRAM1 TRAP1 TRIM25 TRIM28 TRIM32 TRIM56 TRIP4 TRIP10 TRMT1L TRMT2A TRMT6 TRMT10C TSPYL1 TUBB6 TUFM TUT7 U2SURP UBR4 UBTF UGGT1 UQCRC2 USP7 USP9X USP15 USP34 VAPA VAPB VAT1 VCP VDAC3 VPS26A WASL WDR1 WDR5 WDR12 WDR26 WDR82 WIPF2 XPO1 XRCC5 XRCC6 XRN1 XRN2 YTHDC2 YTHDF2 ZC3H18 ZFPL1 ZMPSTE24 ZNF385A
NIH3T3_P	135	36951 5031439G07RIK ABCB1B AFG3L1 AGO3 AK1 ANAPC1 API5 AQP1 ARF2 ARF4 ARL1 ATAD1 ATP5PB AURKB BAG2 BLVRB CAMK1 CAMK2D CBX3 CD2BP2 CDC40 CDK6 CHD4 CIAPIN1 CISD2 CNPY2 CPSF1 CSTF3 CTDSPL2 CYB5B DCAF13 DNMT1 EIF1A EIF2S3X EIF5A FAM32A GLYR1 GM20521 GNS GSTO1 HDAC1 HDAC2 HINT1 HIRIP3 HMGA2 HS2ST1 IAP IFI202 IFITM3 KPNA2 KPNA6 LAMC1 LMNB2 MACROH2A2 MRPL12 MSI2 MTA2 MYADM MYBBP1A MYEF2 NDUFV1 NFU1 NGDN NOTCH2 NRD1 NUP93 NUP107 NUP160 OTUD4 PAFAH1B3 PCM1 PDLIM7 PGAM5 PHC2 PNO1 POLR2E PRDX6 PRKAR2B PRPF4 PRPF6 PRPF31 PRPF40A PRPSAP1 PSMA2 PTBP2 PTPRE RAB1A RAB1B RAB5A RAB5C RAB6A RAB7A RAB11B RAB14 RAC1 RAD21 RALA RANBP1 RBM19 RBMS1 RDH13 RPA1 RRAS2 S100A4 SCML2 SEC22B SH3PXD2B SLC25A4 SLC25A10 SLC29A1 SLC44A2 SLTM SMARCC2 SMARCE1 SMCHD1 SNRNP40 SNRPA SRSF3 ST13 STAG1 STEAP3 STMN1 SYNE3 TMED9 TMED10 TMX1 UBE2L3 UBXN4 UTP3 UTP6 WDR33 YTHDF3 ZFAND6 ZFR
NIH3T3_C	142	2900026A02RIK 8030462N17RIK ACADL AFDN AHNAK2 AK4 ALAD ANLN ANP32E ANXA5 APOA1 ATF7IP ATXN2 BOD1L BSDC1 CAV1 CAVIN2 CCDC50 CD2AP CD34 CD151 CD200 CDC37 CEP170 CFDP1 CNPY4 COBLL1 COL1A2 CRELD2 CSF1 D17H6S53E DAD1 DAG1 DHODH DIDO1 EEF1D EFCAB14 EHD1 EIF3J2 EIF5B EPB41L3 FKBP7 FKBP9 FSTL1 FTL1 FTSJ3 GOLGA5 GOLIM4 GOLT1B GOT1 GRN HAL HEXIM1 HMGN5 HSPA13 IDI1 IRF2BP2 ITGB1 JPT2 KCNAB2 KCT2 KRT76 KRT78 LIG1 LRRFIP1 LRRFIP2 LSM14A LSS LTBP1 LTBP3 MAP1B MAP6 MARCKSL1 MCL1 MDC1 MIX23 NECTIN1 NECTIN3 NRADD NSFL1C PALM PARK7 PDAP1 PDLIM1 PDPN PGAM1 PGM1 PLAUR PLP2 PPP1R7 PPP1R11 PRRC2B PSIP1 PSMD4 PSME3 PTTG1IP PYM1 RABL6 RAD23A RAET1A RANBP3 RPIA RPS21 RSU1 RWDD1 SAFB2 SCPEP1 SEPTIN11 SERPINB6A SERPINF1 SF3B1 SFR1 SGSH SLC12A2 SNW1 SOX9 SPECC1 SPP1 SSR1 STX8 SUPT5H TACC2 TALDO1 TEX101 TGOLN2 TNFRSF10B TPD52L2 TPM3 TSPAN6 TTN TXNDC15 UBQLN1 UBQLN2 ULBP1 USO1 VAMP3 VKORC1L1 VPS26B VSIG10 VTI1A WIPF1 YIPF3
A431_P	262	ABCD3 AGTRAP AJUBA ALDH18A1 ANKLE2 ANXA8 APOBEC3B ARPC1A ARPC1B ARPC4-TTLL3 ATP2C1 B2M B3GAT3 BAIAP2 BCAS2 BOP1 BRIX1 CACYBP CALM2 CALML3 CAMK2G CCDC59 CCNT1 CDC42 CERS2 CHCHD3 CNBP COMT COQ9 CRKL CSE1L CSNK1D CYC1 DDX20 DDX27 DDX41 DHCR7 DIABLO DNAJA1 DNAJB2 DNAJB11 DNAJC3 DNAJC10 DNAJC14 DNAJC16 DOCK7 DPM1 DRG1 EEFSEC EIF3CL EIF3I EIF6 ELOA EMC3 EPB41L4B EPPK1 ERCC6 ERLIN2 EXOSC4 EXOSC6 EXOSC7 FARSA FARSB FBL FBXO31 FOSL1 FTH1 FUBP3 GEMIN5 GFPT1 GM2A GNL2 GPAA1 GPI GTF2H2 GTF3C3 GTF3C4 GTF3C5 H2A HBB HCCS hCG_1984214 HDAC10 HK2 HMGB1 HMGB2 HSP90AB4P HTATIP2 HTRA2 HYAL2 IFRD1 IMMT IMP4 IMPDH IPO7 ISG15 ITPRIP KHSRP KIF1C KRR1 KRT13 KRT15 KRT82 LAD1 LARP4B LLGL1 LRCH3 LYAR M6PR MAGT1 MARK3 MATR3 MBNL1 MCAT MFSD10 MGAT1 MPG MPHOSPH10 MPRIP MRM3 MRPL2 MRPL15 MRPL37 MRPL47 MRPS12 MRPS15 MRPS23 MRPS34 MTAP MX2 MYDGF MYH14 MYL12A MYO18A NAP1L4 NAPA NDUFA10 NDUFS2 NFX1 NIPSNAP1 NOP56 NOP58 NOS1AP NSF NSUN4 NSUN5 NTAQ1 NUDT5 NUMB P3H2 PCBP1 PCMTD1 PCNA PEX16 PFKL PIGT PLAA PLAU PLEC PLEKHG3 PNN POLDIP2 POLDIP3 POLR3A PPL PPP1R10 PPP2R2A PPP3CA PPP6C PRDX4 PRDX5 PRKAR2A PRORP PSMA1 PSMB3 PSMC1 PSMC3 PSMC6 PTBP3 PTK7 PTPMT1 PUM3 PYGL RAB5B RAB8B RAB34 RAB38 RAN RBM27 RBM33 RBM47 RCN2 RFC1 RHOT1 RIDA RPP30 RPS15 RRAGB RRP1B RRP12 RTKN RUVBL1 RUVBL2 S100A6 S100A8 SDF2 SEC61B SEC62 SET SETX SF3A1 SFXN3 SIGMAR1 SLC25A3 SLC35B2 SMN1 SNRPB SPOUT1 SPRR1B SPTY2D1 SRP14 SRP72 SSRP1 STT3A STT3B SURF4 TAP2 TBCA TBL3 TECR TEFM TIMP3 TMCC1 TMEM40 TMEM214 TMX3 TOLLIP TOM1L2 TOP3B TOR1AIP2 TPM2 TPP1 TRIM65 TRMT61A TSG101 TSR1 TTC13 TUBB TUBB4B TWNK UFL1 UNC45A USP10 VDAC1 VDAC2 VPS45 WDR3 WDR36 WDR77 YTHDF1 Z02 ZNF598
A431_C	119	ABCB7 ABHD12 ACOT7 AFAP1 AFG3L2 AGO2 AHCY AHSA1 AK4P3 ANXA8L1 ARFIP1 ATL2 ATP1B1 ATP1B3 ATP5MF-PTCD1 ATP6AP1 BZW1 CAPG CAPN2 CASP14 CAT CCN1 CD46 CHERP CLPX COPB1 CSTA DEFA1 DHX37 DSC3 DSP DYNC1H1 ECHS1 ECM1 EIF2B4 EIF3L EIF5 ERAL1 ERAP1 ERLEC1 ETFA ETFB ETHE1 FAM20B FLG FMNL3 FN1 GARS1 GFM1 GIGYF2 GLUD1 H1-3 HDLBP HEXB HM13 HMGCL HSP90AB1 HSPA4 HSPBP1 HSPD1 IDH2 IGHA1 IGKC JUP KPRP KRT80 LBR LCLAT1 LMAN2L LYZ MAPK1 MCM3 MFN1 MRPL44 NDUFA4 NECTIN2 NFKB2 NUDCD1 NUP155 P4HA1 PIGK PIGS PPT1 PROSER2 PRPS1 PSMB8 PSMD1 PSMD6 PSMD13 PSMD14 PTGFRN PUM1 PUS1 PYCR1 RNF213 S100A14 SACM1L SAMD9 SEC23IP SEPTIN8 SERPINB6 SLC38A5 SMC2 SPRYD3 STK24 STX6 SUCLG2 TBL1XR1 TMEM87A TOMM70 TYMP UBA1 UCHL5 UFD1 UMPS VPS4A VPS35 YARS2 ZG16B

Supplemental table 2 : Summary table of proteins present in A431-Tks5-GFP cells seeded on plastic identified by mass spectrometry analysis of Tks5 interactome

Accession	Gene Name	Description	Abundance Ratio: (TKS5) / (Control)
A0A8I5KQW5	SH3PXD2A	SH3 and PX domains 2A	940,95
P49207	RPL34	Large ribosomal subunit protein eL34	264,35
P46777	RPL5	Large ribosomal subunit protein uL18	187,07
Q13444	ADAM15	Disintegrin and metalloproteinase domain-containing protein 15	159,48
P62917	RPL8	Large ribosomal subunit protein uL2	159,47
Q12986	NFX1	Transcriptional repressor NF-X1	153,84
P62701	RPS4X	Small ribosomal subunit protein eS4, X isoform	152,36
A0A8Q3SIY9	AFAP1L2	Actin filament associated protein 1 like 2	113,97
P05388	RPLP0	Large ribosomal subunit protein uL10	100,28
P62241	RPS8	Small ribosomal subunit protein eS8	92,09
O75915	ARL6IP5	PRA1 family protein 3	90,94
F8W930	IGF2BP2	Insulin like growth factor 2 mRNA binding protein 2	88,29
P39023	RPL3	Large ribosomal subunit protein uL3	82,48
A0A8I5KWT8	MYH9	Myosin heavy chain 9	76,76
P57678	GEMIN4	Gem-associated protein 4	74,98
P62906	RPL10A	Large ribosomal subunit protein uL1	74,37
P54619	PRKAG1	5'-AMP-activated protein kinase subunit gamma-1	71,19
P48651	PTDSS1	Phosphatidylserine synthase 1	70,35
P50914	RPL14	Large ribosomal subunit protein eL14	69,93
A0A8V8TQ35	FNBP1	Formin binding protein 1	61,72
B7ZM99	MTHFD1L	formate--tetrahydrofolate ligase	60,60
P26373	RPL13	Large ribosomal subunit protein eL13	60,39
P36578	RPL4	Large ribosomal subunit protein uL4	60,00
O43795	MYO1B	Unconventional myosin-Ib	59,47
Q12965	MYO1E	Unconventional myosin-Ie	58,86
Q13131	PRKAA1	5'-AMP-activated protein kinase catalytic subunit alpha-1	55,76
Q9HAN9	NMNAT1	Nicotinamide/nicotinic acid mononucleotide adenylyltransferase 1	55,56
P62424	RPL7A	Large ribosomal subunit protein eL8	53,81
P61247	RPS3A	Small ribosomal subunit protein eS1	52,44
P27635	RPL10	Large ribosomal subunit protein uL16	52,08
P61981	YWHAG	14-3-3 protein gamma	52,05
P46060	RANGAP1	Ran GTPase-activating protein 1	50,34
Q9H013	ADAM19	Disintegrin and metalloproteinase domain-containing protein 19	47,83
A0A7I2V535	NONO	Non-POU domain containing octamer binding	47,40
O94766	B3GAT3	Galactosylgalactosylxylosylprotein 3-beta-glucuronosyltransferase 3	46,96
P62753	RPS6	Small ribosomal subunit protein eS6	46,36
P18564	ITGB6	Integrin beta-6	45,58
Q02543	RPL18A	Large ribosomal subunit protein eL20	45,17
A0A3B3ITT5	RPL29	60S ribosomal protein L29	44,77
P27348	YWHAQ	14-3-3 protein theta	41,61
A0A8V8TMQ1	FXR1	RNA-binding protein FXR1	41,06
Q96I25	RBM17	Splicing factor 45	40,77
P38919	EIF4A3	Eukaryotic initiation factor 4A-III	40,71
Q92522	H1-10	Histone H1.10	40,20
A0A1Y0BRN3	MYO6	Unconventional myosin-VI	38,57
O00159	MYO1C	Unconventional myosin-Ic	37,76
O95793	STAU1	Double-stranded RNA-binding protein Staufen homolog 1	37,56
P62258	YWHAE	14-3-3 protein epsilon	37,47
P83111	LACTB	Serine beta-lactamase-like protein LACTB, mitochondrial	37,00
J3KTA4	DDX5	Probable ATP-dependent RNA helicase DDX5	36,86
P53621	COPA	Coatomer subunit alpha	36,43
P61158	ACTR3	Actin-related protein 3	35,34
Q9Y305	ACOT9	Acyl-coenzyme A thioesterase 9, mitochondrial	35,02
O15042	U2SURP	U2 snRNP-associated SURP motif-containing protein	34,30
A0A0A0MRX1	ELAVL2	ELAV-like protein	34,30
Q9Y446	PKP3	Plakophilin-3	34,08
Q9BUJ2	HNRNPUL1	Heterogeneous nuclear ribonucleoprotein U-like protein 1	33,23
Q7Z417	NUFIP2	FMR1-interacting protein NUFIP2	33,22
Q9Y676	MRPS18B	Small ribosomal subunit protein mS40	33,14
P52272	HNRNPM	Heterogeneous nuclear ribonucleoprotein M	32,87
A0A087WZ40	MAP7	Microtubule associated protein 7	32,60
Q9P015	MRPL15	Large ribosomal subunit protein uL15m	32,56
O60506	SYNCRIP	Heterogeneous nuclear ribonucleoprotein Q	32,02
Q16787	LAMA3	Laminin subunit alpha-3	31,92
Q13724	MOGS	Mannosyl-oligosaccharide glucosidase	31,14
Q9NUQ6	SPATS2L	SPATS2-like protein	31,08
Q9Y4I1	MYO5A	Unconventional myosin-Va	30,85
P62854	RPS26	Small ribosomal subunit protein eS26	30,80
P18124	RPL7	Large ribosomal subunit protein uL30	30,60
Q9Y3U8	RPL36	Large ribosomal subunit protein eL36	30,44
A0A994J6B4	FIP1L1	Pre-mRNA 3'-end-processing factor FIP1	30,28
O75909	CCNK	Cyclin-K	30,03
Q8IXQ6	PARP9	Protein mono-ADP-ribosyltransferase PARP9	29,55
J3QLS3	MRPS7	Mitochondrial ribosomal protein S7	29,12
A0A0A6YYL6	RPL17-C18orf32	60S ribosomal protein L17	28,89
Q7Z406	MYH14	Myosin-14	28,80
Q7KZI7	MARK2	Serine/threonine-protein kinase MARK2	28,56
P31946	YWHAB	14-3-3 protein beta/alpha	28,44
Q13310	PABPC4	Polyadenylate-binding protein 4	28,39
P05362	ICAM1	Intercellular adhesion molecule 1	28,33
Q02878	RPL6	Large ribosomal subunit protein eL6	27,58
Q8NFW8	CMAS	N-acylneuraminate cytidylyltransferase	27,39
Q12906	ILF3	Interleukin enhancer-binding factor 3	27,30
Q86V48	LUZP1	Leucine zipper protein 1	27,24
H3BPE7	FUS	FUS RNA binding protein	27,21
A0A7I2YQG2	RPL19	Ribosomal protein L19	26,42
Q13601	KRR1	KRR1 small subunit processome component homolog	26,38
O15162	PLSCR1	Phospholipid scramblase 1	26,37
Q9ULV4	CORO1C	Coronin-1C	26,02
Q08211	DHX9	ATP-dependent RNA helicase A	26,01
P11387	TOP1	DNA topoisomerase 1	25,90
Q14258	TRIM25	E3 ubiquitin/ISG15 ligase TRIM25	25,90
O75569	PRKRA	Interferon-inducible double-stranded RNA-dependent protein kinase activator A	25,68
A0A5H1ZRQ2	DDX17	RNA helicase	25,54
A0A8I5KNN2	DDX21	RNA helicase	25,46
A0A3B3ITA3	C8orf33	Chromosome 8 open reading frame 33	25,40
Q12797	ASPH	Aspartyl/asparaginyl beta-hydroxylase	25,20
Q9H0A0	NAT10	RNA cytidine acetyltransferase	24,89
Q9NVI7	ATAD3A	ATPase family AAA domain-containing protein 3A	24,83
E7ERS3	ZC3H18	Zinc finger CCCH-type containing 18	24,77
Q04917	YWHAH	14-3-3 protein eta	24,68
Q99575	POP1	Ribonucleases P/MRP protein subunit POP1	24,62
A0A8I5KZ38	MYH10	Myosin heavy chain 10	24,42
Q00839	HNRNPU	Heterogeneous nuclear ribonucleoprotein U	24,33
Q86YT6	MIB1	E3 ubiquitin-protein ligase MIB1	24,31
O00401	WASL	Actin nucleation-promoting factor WASL	24,31
P46778	RPL21	Large ribosomal subunit protein eL21	24,23
P11940	PABPC1	Polyadenylate-binding protein 1	24,15
P23396	RPS3	Small ribosomal subunit protein uS3	24,13
A0A8Q3WMA7	SFPQ	Splicing factor proline and glutamine rich	23,39
O94832	MYO1D	Unconventional myosin-Id	23,38
Q9BQ67	GRWD1	Glutamate-rich WD repeat-containing protein 1	23,38
Q9NYL9	TMOD3	Tropomodulin-3	23,10
A0A0U1RRM4	PTBP1	Polypyrimidine tract-binding protein 1	23,00
Q9Y394	DHRS7	Dehydrogenase/reductase SDR family member 7	22,94
O00425	IGF2BP3	Insulin-like growth factor 2 mRNA-binding protein 3	22,91
P20592	MX2	Interferon-induced GTP-binding protein Mx2	22,90
P61313	RPL15	Large ribosomal subunit protein eL15	22,70
Q92499	DDX1	ATP-dependent RNA helicase DDX1	22,57
O43143	DHX15	ATP-dependent RNA helicase DHX15	22,17
A7XZE4	TPM2	Beta tropomyosin isoform	22,14
A0A5F9ZI21	ARHGEF2	Rho guanine nucleotide exchange factor 2	22,03
P51398	DAP3	Small ribosomal subunit protein mS29	21,98
Q9Y478	PRKAB1	5'-AMP-activated protein kinase subunit beta-1	21,82
Q6WCQ1	MPRIP	Myosin phosphatase Rho-interacting protein	21,78
O95782	AP2A1	AP-2 complex subunit alpha-1	21,36
Q9H2U1	DHX36	ATP-dependent DNA/RNA helicase DHX36	21,23
Q7Z2W4	ZC3HAV1	Zinc finger CCCH-type antiviral protein 1	21,15
Q5STZ8	ABCF1	ATP binding cassette subfamily F member 1	21,10
Q9HC36	MRM3	rRNA methyltransferase 3, mitochondrial	21,06
Q9H0U9	TSPYL1	Testis-specific Y-encoded-like protein 1	20,83
Q9BZE4	GTPBP4	GTP-binding protein 4	20,78
Q9UN81	L1RE1	LINE-1 retrotransposable element ORF1 protein	20,73
Q9UHB6	LIMA1	LIM domain and actin-binding protein 1	20,69
Q13191	CBLB	E3 ubiquitin-protein ligase CBL-B	20,66
Q86XL3	ANKLE2	Ankyrin repeat and LEM domain-containing protein 2	20,49
A0A2U3TZU2	GPI	Glucose-6-phosphate isomerase	20,30
Q9GZR7	DDX24	ATP-dependent RNA helicase DDX24	20,28
A0A087X009	ABO	ABO, alpha 1-3-N-acetylgalactosaminyltransferase and alpha 1-3-galactosyltransferase	20,16
Q96AG4	LRRC59	Leucine-rich repeat-containing protein 59	20,11
O43390	HNRNPR	Heterogeneous nuclear ribonucleoprotein R	20,09
Q86UE4	MTDH	Protein LYRIC	20,04
Q9NW13	RBM28	RNA-binding protein 28	19,92
Q9NXH8	TOR4A	Torsin-4A	19,77
P46940	IQGAP1	Ras GTPase-activating-like protein IQGAP1	19,77
P82933	MRPS9	Small ribosomal subunit protein uS9m	19,75
A0A8Q3WKF1	MRPS27	Mitochondrial ribosomal protein S27	19,61
P13010	XRCC5	X-ray repair cross-complementing protein 5	19,55
Q15629	TRAM1	Translocating chain-associated membrane protein 1	19,40
Q8IY21	DDX60	Probable ATP-dependent RNA helicase DDX60	19,40
Q9BRJ6	C7orf50	Uncharacterized protein C7orf50	19,35
A0A994J4D9	EXOSC4	Exosome component 4	19,35
Q6PKG0	LARP1	La-related protein 1	19,27
A0A590UJ43	CSNK1A1	Casein kinase 1 alpha 1	19,17
A0A8C8NWT4	PLEKHG3	Pleckstrin homology and RhoGEF domain containing G3	19,15
P63104	YWHAZ	14-3-3 protein zeta/delta	19,02
A0A2R8Y7R9	PPFIA1	PTPRF interacting protein alpha 1 (Fragment)	18,95
A0A1W2PPR6	PIGT	Phosphatidylinositol glycan anchor biosynthesis class T	18,94
Q9Y4P3	TBL2	Transducin beta-like protein 2	18,94
H7C2Q8	EBNA1BP2	EBNA1 binding protein 2	18,86
P46776	RPL27A	Large ribosomal subunit protein uL15	18,85
Q55RQ6	CSNK2B	Casein kinase II subunit beta	18,78
P62888	RPL30	Large ribosomal subunit protein eL30	18,66
Q8WU90	ZC3H15	Zinc finger CCCH domain-containing protein 15	18,59
Q5T653	MRPL2	Large ribosomal subunit protein uL2m	18,46
Q14103	HNRNPD	Heterogeneous nuclear ribonucleoprotein D0	18,38
E7EVA0	MAP4	Microtubule-associated protein	18,29
P62266	RPS23	Small ribosomal subunit protein uS12	18,22
Q6ZMY3	SPOCD1	SPOC domain-containing protein 1	18,19
C9JJ19	MRPS34	Mitochondrial ribosomal protein S34	18,17
O75822	EIF3J	Eukaryotic translation initiation factor 3 subunit J	18,13
Q92544	TM9SF4	Transmembrane 9 superfamily member 4	18,03
P16989	YBX3	Y-box-binding protein 3	18,01
P53992	SEC24C	Protein transport protein Sec24C	17,81
Q16666	IFI16	Gamma-interferon-inducible protein 16	17,78
Q13610	PWP1	Periodic tryptophan protein 1 homolog	17,69
Q9UGJ0	PRKAG2	5'-AMP-activated protein kinase subunit gamma-2	17,69
Q9Y3I0	RTCB	RNA-splicing ligase RtcB homolog	17,67
P35606	COPB2	Coatomer subunit beta'	17,37
Q8IXI1	RHOT2	Mitochondrial Rho GTPase 2	17,31
P30050	RPL12	Large ribosomal subunit protein uL11	17,29
Q13643	FHL3	Four and a half LIM domains protein 3	17,29
J3QQ67	RPL18	Ribosomal protein L18 (Fragment)	17,19
A0A0A0MTI1	DHCR24	24-dehydrocholesterol reductase	17,15
P06396	GSN	Gelsolin	17,15
Q7L2E3	DHX30	ATP-dependent RNA helicase DHX30	17,14
O00411	POLRMT	DNA-directed RNA polymerase, mitochondrial	17,07
Q9UMS4	PRPF19	Pre-mRNA-processing factor 19	17,07
O15143	ARPC1B	Actin-related protein 2/3 complex subunit 1B	17,03
Q562R1	ACTBL2	Beta-actin-like protein 2	16,92
P47755	CAPZA2	F-actin-capping protein subunit alpha-2	16,85
P61160	ACTR2	Actin-related protein 2	16,64
A0A7P0T8C7	SNX9	Sorting nexin 9	16,62
Q9Y6H1	CHCHD2	Coiled-coil-helix-coiled-coil-helix domain-containing protein 2	16,62
P67809	YBX1	Y-box-binding protein 1	16,61
P26196	DDX6	Probable ATP-dependent RNA helicase DDX6	16,60
Q9NZB2	FAM120A	Constitutive coactivator of PPAR-gamma-like protein 1	16,58
P62829	RPL23	Large ribosomal subunit protein uL14	16,28
P09874	PARP1	Poly [ADP-ribose] polymerase 1	16,24
Q9H0D6	XRN2	5'-3' exoribonuclease 2	16,23
Q07065	CKAP4	Cytoskeleton-associated protein 4	16,19
Q9NZM1	MYOF	Myoferlin	16,17
H7BY16	NCL	Nucleolin	16,11
O43663	PRC1	Protein regulator of cytokinesis 1	16,11
O43293	DAPK3	Death-associated protein kinase 3	15,90
I3L4N8	ACTG1	Actin gamma 1	15,89
P46063	RECQL	ATP-dependent DNA helicase Q1	15,83
Q9H089	LSG1	Large subunit GTPase 1 homolog	15,71
Q13011	ECH1	Delta(3,5)-Delta(2,4)-dienoyl-CoA isomerase, mitochondrial	15,69
Q9BRZ2	TRIM56	E3 ubiquitin-protein ligase TRIM56	15,61
O94925	GLS	Glutaminase kidney isoform, mitochondrial	15,55
Q5T0N5	FNBP1L	Formin-binding protein 1-like	15,53
A0A6Q8PG37	KIF2A	Kinesin-like protein	15,50
Q9Y2P8	RCL1	RNA 3'-terminal phosphate cyclase-like protein	15,46
P50281	MMP14	Matrix metalloproteinase-14	15,45
A1L170	C1orf226	Uncharacterized protein C1orf226	15,41
Q15646	OASL	2'-5'-oligoadenylate synthase-like protein	15,40
H7BYT1	CSNK1D	Casein kinase 1 delta	15,38
Q14694	USP10	Ubiquitin carboxyl-terminal hydrolase 10	15,27
P56182	RRP1	Ribosomal RNA processing protein 1 homolog A	15,23
Q9BTT6	LRRC1	Leucine-rich repeat-containing protein 1	15,22
M0QXB4	COPE	Coatomer subunit epsilon	15,14
O75165	DNAJC13	DnaJ homolog subfamily C member 13	15,13
Q8WVM8	SCFD1	Sec1 family domain-containing protein 1	15,09
Q6ZXV5	TMTC3	Protein O-mannosyl-transferase TMTC3	15,06
O95425	SVIL	Supervillin	15,05
Q9NQT4	EXOSC5	Exosome complex component RRP46	15,01
P48634	PRRC2A	Protein PRRC2A	15,01
P05198	EIF2S1	Eukaryotic translation initiation factor 2 subunit 1	15,00
Q99459	CDC5L	Cell division cycle 5-like protein	14,98
Q8IZP0	ABI1	Abl interactor 1	14,98
O75676	RPS6KA4	Ribosomal protein S6 kinase alpha-4	14,91
P29728	OAS2	2'-5'-oligoadenylate synthase 2	14,88
P09651	HNRNPA1	Heterogeneous nuclear ribonucleoprotein A1	14,87
Q96J02	ITCH	E3 ubiquitin-protein ligase Itchy homolog	14,84
P41091	EIF2S3	Eukaryotic translation initiation factor 2 subunit 3	14,73
P19525	EIF2AK2	Interferon-induced, double-stranded RNA-activated protein kinase	14,72
P63010	AP2B1	AP-2 complex subunit beta	14,69
P07954	FH	Fumarate hydratase, mitochondrial	14,65
O15091	PRORP	Mitochondrial ribonuclease P catalytic subunit	14,65
P49748	ACADVL	Very long-chain specific acyl-CoA dehydrogenase, mitochondrial	14,61
Q13263	TRIM28	Transcription intermediary factor 1-beta	14,52
Q96FW1	OTUB1	Ubiquitin thioesterase OTUB1	14,45
Q15046	KARS1	Lysine--tRNA ligase	14,42
P46087	NOP2	Probable 28S rRNA (cytosine(4447)-C(5))-methyltransferase	14,35
P32969	RPL9	Large ribosomal subunit protein uL6	14,33
J3KPT4	TRABD	TraB domain containing	14,30
Q9Y6N5	SQOR	Sulfide:quinone oxidoreductase, mitochondrial	14,26
A0A6Q8PFE1	AIFM1	Apoptosis inducing factor mitochondria associated 1	14,22
P52758	RIDA	2-iminobutanoate/2-iminopropanoate deaminase	14,18
G3V1L9	TJP1	Tight junction protein 1	14,08
O95470	SGPL1	Sphingosine-1-phosphate lyase 1	14,06
H7BYY1	TPM1	Tropomyosin 1	14,05
P61011	SRP54	Signal recognition particle subunit SRP54	14,02
P22626	HNRNPA2B1	Heterogeneous nuclear ribonucleoproteins A2/B1	14,01
Q12792	TWF1	Twinfilin-1	13,96
Q96RR1	TWNK	Twinkle mtDNA helicase	13,96
E9PQ57	RAE1	Ribonucleic acid export 1	13,91
P08240	SRPRA	Signal recognition particle receptor subunit alpha	13,85
Q8NCA5	FAM98A	Protein FAM98A	13,83
Q9P2E9	RRBP1	Ribosome-binding protein 1	13,75
A0A7P0TAU8	OAS3	2'-5' oligoadenylate synthase	13,68
Q70UQ0	IKBIP	Inhibitor of nuclear factor kappa-B kinase-interacting protein	13,56
Q8TCJ2	STT3B	Dolichyl-diphosphooligosaccharide--protein glycosyltransferase subunit STT3B	13,56
P14868	DARS1	Aspartate--tRNA ligase, cytoplasmic	13,55
O75534	CSDE1	Cold shock domain-containing protein E1	13,53
Q9P2N5	RBM27	RNA-binding protein 27	13,47
Q9P2J5	LARS1	Leucine--tRNA ligase, cytoplasmic	13,47
E7ETE2	HARS1	Histidine--tRNA ligase, cytoplasmic	13,44
H0YFD6	HADHA	enoyl-CoA hydratase	13,30
P10398	ARAF	Serine/threonine-protein kinase A-Raf	13,27
Q9P206	KIAA1522	Uncharacterized protein KIAA1522	13,26
Q12905	ILF2	Interleukin enhancer-binding factor 2	13,24
Q9NVN8	GNL3L	Guanine nucleotide-binding protein-like 3-like protein	13,23
Q9BZF1	OSBPL8	Oxysterol-binding protein-related protein 8	13,09
C9JEJ2	PCYT1A	choline-phosphate cytidylyltransferase	13,04
Q9Y4F5	CEP170B	Centrosomal protein of 170 kDa protein B	12,94
A0A0A0MSV9	TAPBP	Tapasin	12,94
Q9NVV4	MTPAP	Poly(A) RNA polymerase, mitochondrial	12,89
P07199	CENPB	Major centromere autoantigen B	12,88
E9PB61	ALYREF	Aly/REF export factor	12,77
Q15650	TRIP4	Activating signal cointegrator 1	12,77
X6R8F3	LCN2	Lipocalin 2	12,76
P35221	CTNNA1	Catenin alpha-1	12,62
P30740	SERPINB1	Leukocyte elastase inhibitor	12,60
P05783	KRT18	Keratin, type I cytoskeletal 18	12,53
Q15020	SART3	Squamous cell carcinoma antigen recognized by T-cells 3	12,47
P05161	ISG15	Ubiquitin-like protein ISG15	12,46
P42677	RPS27	Small ribosomal subunit protein eS27	12,33
Q96FX7	TRMT61A	tRNA (adenine(58)-N(1))-methyltransferase catalytic subunit TRMT61A	12,29
P62913	RPL11	Large ribosomal subunit protein uL5	12,25
A0A8V8TNT7	WDR1	WD repeat domain 1	12,20
Q00577	PURA	Transcriptional activator protein Pur-alpha	12,19
Q13151	HNRNPA0	Heterogeneous nuclear ribonucleoprotein A0	12,19
Q9Y399	MRPS2	Small ribosomal subunit protein uS2m	12,18
Q658Y4	FAM91A1	Protein FAM91A1	12,16
Q96PK6	RBM14	RNA-binding protein 14	12,14
O94851	MICAL2	[F-actin]-monooxygenase MICAL2	12,12
P84022	SMAD3	Mothers against decapentaplegic homolog 3	12,12
Q9UJA5	TRMT6	tRNA (adenine(58)-N(1))-methyltransferase non-catalytic subunit TRM6	12,11
F6VRR5	POLDIP3	DNA polymerase delta interacting protein 3	12,01
Q8IVL5	P3H2	Prolyl 3-hydroxylase 2	12,00
Q14004	CDK13	Cyclin-dependent kinase 13	12,00
C9JRZ6	CHCHD3	Coiled-coil-helix-coiled-coil-helix domain containing 3	11,97
A0A494C0G7	DIMT1	rRNA adenine N(6)-methyltransferase	11,96
Q6NZI2	CAVIN1	Caveolae-associated protein 1	11,95
Q08AF3	SLFN5	Schlafen family member 5	11,88
O43865	AHCYL1	S-adenosylhomocysteine hydrolase-like protein 1	11,86
Q9GZL7	WDR12	Ribosome biogenesis protein WDR12	11,85
Q02809	PLOD1	Procollagen-lysine,2-oxoglutarate 5-dioxygenase 1	11,82
P26368	U2AF2	Splicing factor U2AF 65 kDa subunit	11,80
Q3KQU3	MAP7D1	MAP7 domain-containing protein 1	11,77
Q92900	UPF1	Regulator of nonsense transcripts 1	11,76
Q6UY14	ADAMTSL4	ADAMTS-like protein 4	11,75
P27105	STOM	Stomatin	11,74
Q8IWT6	LRRC8A	Volume-regulated anion channel subunit LRRC8A	11,72
Q12788	TBL3	Transducin beta-like protein 3	11,66
H0Y6G2	CAMK2G	calcium/calmodulin-dependent protein kinase	11,62
Q6ICG6	KIAA0930	Uncharacterized protein KIAA0930	11,56
P42285	MTREX	Exosome RNA helicase MTR4	11,56
Q9BYJ9	YTHDF1	YTH domain-containing family protein 1	11,53
Q8TDB6	DTX3L	E3 ubiquitin-protein ligase DTX3L	11,50
Q7Z2T5	TRMT1L	TRMT1-like protein	11,47
Q6NUQ4	TMEM214	Transmembrane protein 214	11,46
A0A8I5KX72	NKRF	NFKB repressing factor	11,43
A0A804HKI2	LRPPRC	Leucine rich pentatricopeptide repeat containing	11,42
M0R2N5	TECR	very-long-chain enoyl-CoA reductase	11,41
Q07666	KHDRBS1	KH domain-containing, RNA-binding, signal transduction-associated protein 1	11,40
Q16531	DDB1	DNA damage-binding protein 1	11,31
O00566	MPHOSPH10	U3 small nucleolar ribonucleoprotein protein MPP10	11,30
Q15334	LLGL1	Lethal(2) giant larvae protein homolog 1	11,30
P62195	PSMC5	26S proteasome regulatory subunit 8	11,25
Q9BVP2	GNL3	Guanine nucleotide-binding protein-like 3	11,23
P83731	RPL24	Large ribosomal subunit protein eL24	11,20
Q96QR8	PURB	Transcriptional activator protein Pur-beta	11,17
Q8NEZ5	FBXO22	F-box only protein 22	11,16
Q9Y6X9	MORC2	ATPase MORC2	11,10
O75934	BCAS2	Pre-mRNA-splicing factor SPF27	11,10
Q9UK99	FBXO3	F-box only protein 3	11,08
P07305	H1-0	Histone H1.0	11,07
P50750	CDK9	Cyclin-dependent kinase 9	11,07
O95573	ACSL3	Fatty acid CoA ligase Acsl3	11,05
A0A5F9ZHP1	ASCC1	Activating signal cointegrator 1 complex subunit 1	11,04
P54136	RARS1	Arginine--tRNA ligase, cytoplasmic	11,01
P15880	RPS2	Small ribosomal subunit protein uS5	10,96
Q9BSJ8	ESYT1	Extended synaptotagmin-1	10,93
Q06265	EXOSC9	Exosome complex component RRP45	10,93
Q8NHQ9	DDX55	ATP-dependent RNA helicase DDX55	10,87
P16401	H1-5	Histone H1.5	10,87
P62280	RPS11	Small ribosomal subunit protein uS17	10,82
Q8N3C0	ASCC3	Activating signal cointegrator 1 complex subunit 3	10,77
Q9HCE1	MOV10	Helicase MOV-10	10,74
P07996	THBS1	Thrombospondin-1	10,73
Q92747	ARPC1A	Actin-related protein 2/3 complex subunit 1A	10,72
O15144	ARPC2	Actin-related protein 2/3 complex subunit 2	10,72
Q99832	CCT7	T-complex protein 1 subunit eta	10,72
M0QYS1	RPL13A	60S ribosomal protein L13a (Fragment)	10,70
M0QYN0	MYDGF	Myeloid derived growth factor	10,66
Q9BVJ6	UTP14A	U3 small nucleolar RNA-associated protein 14 homolog A	10,63
Q03169	TNFAIP2	Tumor necrosis factor alpha-induced protein 2	10,62
Q8WWA1	TMEM40	Transmembrane protein 40	10,61
O75208	COQ9	Ubiquinone biosynthesis protein COQ9, mitochondrial	10,58
P43358	MAGEA4	Melanoma-associated antigen 4	10,54
Q9NTJ3	SMC4	Structural maintenance of chromosomes protein 4	10,45
Q15436	SEC23A	Protein transport protein Sec23A	10,37
P20042	EIF2S2	Eukaryotic translation initiation factor 2 subunit 2	10,35
B7Z6Z4	MYL6	Myosin light chain 6	10,34
Q99755	PIP5K1A	Phosphatidylinositol 4-phosphate 5-kinase type-1 alpha	10,31
Q14554	PDIA5	Protein disulfide-isomerase A5	10,31
Q86XZ4	SPATS2	Spermatogenesis-associated serine-rich protein 2	10,30
Q9H6F5	CCDC86	Coiled-coil domain-containing protein 86	10,30
Q8NFQ8	TOR1AIP2	Torsin-1A-interacting protein 2	10,29
P52597	HNRNPF	Heterogeneous nuclear ribonucleoprotein F	10,29
Q14192	FHL2	Four and a half LIM domains protein 2	10,26
Q9NS86	LANCL2	LanC-like protein 2	10,25
P98174	FGD1	FYVE, RhoGEF and PH domain-containing protein 1	10,25
Q8TB61	SLC35B2	Adenosine 3'-phospho 5'-phosphosulfate transporter 1	10,24
Q14435	GALNT3	Polypeptide N-acetylgalactosaminyltransferase 3	10,23
Q6WKZ4	RAB11FIP1	Rab11 family-interacting protein 1	10,23
Q13895	BYSL	Bystin	10,19
O43896	KIF1C	Kinesin-like protein KIF1C	10,15
O00541	PES1	Pescadillo homolog	10,14
A0A7I2V647	G3BP2	G3BP stress granule assembly factor 2	10,11
P05787	KRT8	Keratin, type II cytoskeletal 8	10,10
A0A669KB89	SCRIB	Scribble planar cell polarity protein	10,09
Q9UG56	PISD	Phosphatidylserine decarboxylase proenzyme, mitochondrial	10,08
Q2NL82	TSR1	Pre-rRNA-processing protein TSR1 homolog	10,08
Q9H0E2	TOLLIP	Toll-interacting protein	10,04
A0A669KBL1	USP7	Ubiquitin carboxyl-terminal hydrolase 7	10,02
P22223	CDH3	Cadherin-3	10,01
Q9UKD2	MRTO4	mRNA turnover protein 4 homolog	10,00
P55265	ADAR	Double-stranded RNA-specific adenosine deaminase	9,94
Q7Z333	SETX	Probable helicase senataxin	9,91
A0A994J7P9	FXR2	FMR1 autosomal homolog 2	9,91
E9PIE3	CAVIN3	Caveolae associated protein 3	9,85
P07814	EPRS1	Bifunctional glutamate/proline--tRNA ligase	9,84
P82673	MRPS35	Small ribosomal subunit protein mS35	9,84
A0A1W2PNX8	UNC45A	Unc-45 myosin chaperone A	9,83
O95985	TOP3B	DNA topoisomerase 3-beta-1	9,82
Q8WWM7	ATXN2L	Ataxin-2-like protein	9,80
Q96EY1	DNAJA3	DnaJ homolog subfamily A member 3, mitochondrial	9,78
H0YDT6	EIF3F	Eukaryotic translation initiation factor 3 subunit F	9,75
P18077	RPL35A	Large ribosomal subunit protein eL33	9,70
Q00059	TFAM	Transcription factor A, mitochondrial	9,68
Q14444	CAPRIN1	Caprin-1	9,61
Q15019	SEPTIN2	Septin-2	9,61
Q6NUM9	RETSAT	All-trans-retinol 13,14-reductase	9,61
P13861	PRKAR2A	cAMP-dependent protein kinase type II-alpha regulatory subunit	9,60
Q9NSB4	KRT82	Keratin, type II cuticular Hb2	9,60
Q9H0S4	DDX47	Probable ATP-dependent RNA helicase DDX47	9,59
P13928	ANXA8	Annexin A8	9,57
Q13155	AIMP2	Aminoacyl tRNA synthase complex-interacting multifunctional protein 2	9,56
Q13283	G3BP1	Ras GTPase-activating protein-binding protein 1	9,53
Q14008	CKAP5	Cytoskeleton-associated protein 5	9,52
P51665	PSMD7	26S proteasome non-ATPase regulatory subunit 7	9,47
Q70CQ2	USP34	Ubiquitin carboxyl-terminal hydrolase 34	9,43
P82675	MRPS5	Small ribosomal subunit protein uS5m	9,39
Q9Y520	PRRC2C	Protein PRRC2C	9,35
Q5VZM2	RRAGB	Ras-related GTP-binding protein B	9,32
P37108	SRP14	Signal recognition particle 14 kDa protein	9,32
A0A2R8YDH4	ZO2	Tight junction protein ZO-2	9,27
Q13049	TRIM32	E3 ubiquitin-protein ligase TRIM32	9,25
P35249	RFC4	Replication factor C subunit 4	9,23
Q15717	ELAVL1	ELAV-like protein 1	9,22
Q9UNX4	WDR3	WD repeat-containing protein 3	9,21
P47897	QARS1	Glutamine--tRNA ligase	9,18
Q12907	LMAN2	Vesicular integral-membrane protein VIP36	9,17
Q9Y5B9	SUPT16H	FACT complex subunit SPT16	9,16
Q12849	GRSF1	G-rich sequence factor 1	9,16
P21964	COMT	Catechol O-methyltransferase	9,14
A0A8J9AUI2	RAB3GAP1	Rab3 GTPase-activating protein catalytic subunit	9,12
Q8WUA4	GTF3C2	General transcription factor 3C polypeptide 2	9,09
Q9UKN8	GTF3C4	General transcription factor 3C polypeptide 4	9,09
B4E2Q0	ATP2C1	Calcium-transporting ATPase	9,09
Q9Y2G8	DNAJC16	DnaJ homolog subfamily C member 16	9,07
Q13888	GTF2H2	General transcription factor IIH subunit 2	8,98
O60437	PPL	Periplakin	8,98
A0A087WX97	BCL2L13	BCL2 like 13	8,98
Q9P031	CCDC59	Thyroid transcription factor 1-associated protein 26	8,95
K7EJJ5	CLTC	Clathrin heavy chain	8,89
Q6ZMP0	THSD4	Thrombospondin type-1 domain-containing protein 4	8,87
O14974	PPP1R12A	Protein phosphatase 1 regulatory subunit 12A	8,87
E9PFW3	AP2M1	AP-2 complex subunit mu	8,85
Q9P258	RCC2	Protein RCC2	8,85
Q96EY7	PTCD3	Small ribosomal subunit protein mS39	8,85
Q8IVS2	MCAT	Malonyl-CoA-acyl carrier protein transacylase, mitochondrial	8,81
P15407	FOSL1	Fos-related antigen 1	8,80
Q9C0C2	TNKS1BP1	182 kDa tankyrase-1-binding protein	8,80
Q9ULV0	MYO5B	Unconventional myosin-Vb	8,80
P53701	HCCS	Holocytochrome c-type synthase	8,77
A8MXP9	MATR3	Matrin 3	8,70
P55084	HADHB	Trifunctional enzyme subunit beta, mitochondrial	8,69
Q14451	GRB7	Growth factor receptor-bound protein 7	8,67
P68400	CSNK2A1	Casein kinase II subunit alpha	8,65
Q92665	MRPS31	Small ribosomal subunit protein mS31	8,65
P46781	RPS9	Small ribosomal subunit protein uS4	8,61
P42766	RPL35	Large ribosomal subunit protein uL29	8,58
A0A0G2JLV0	TAP2	ABC-type antigen peptide transporter	8,50
Q15459	SF3A1	Splicing factor 3A subunit 1	8,48
Q68D10	SPTY2D1	Protein SPT2 homolog	8,47
Q9Y3F4	STRAP	Serine-threonine kinase receptor-associated protein	8,45
Q8NBJ4	GOLM1	Golgi membrane protein 1	8,45
Q9P035	HACD3	Very-long-chain (3R)-3-hydroxyacyl-CoA dehydratase 3	8,44
Q09161	NCBP1	Nuclear cap-binding protein subunit 1	8,44
Q9BW92	TARS2	Threonine--tRNA ligase, mitochondrial	8,40
Q8WVM0	TFB1M	Dimethyladenosine transferase 1, mitochondrial	8,36
Q9H5Q4	TFB2M	Dimethyladenosine transferase 2, mitochondrial	8,36
P04259	KRT6B	Keratin, type II cytoskeletal 6B	8,36
Q15006	EMC2	ER membrane protein complex subunit 2	8,36
P35268	RPL22	Large ribosomal subunit protein eL22	8,33
Q9NWT1	PAK1IP1	p21-activated protein kinase-interacting protein 1	8,32
Q9H3Z4	DNAJC5	DnaJ homolog subfamily C member 5	8,32
Q7KZF4	SND1	Staphylococcal nuclease domain-containing protein 1	8,26
O75955	FLOT1	Flotillin-1	8,26
Q9H9L3	ISG20L2	Interferon-stimulated 20 kDa exonuclease-like 2	8,25
P62191	PSMC1	26S proteasome regulatory subunit 4	8,22
A0A5F9ZHN9	ALDH3A2	Aldehyde dehydrogenase	8,21
O75390	CS	Citrate synthase, mitochondrial	8,15
H3BNC9	RPS17	40S ribosomal protein S17	8,15
Q92614	MYO18A	Unconventional myosin-XVIIIa	8,11
Q9H6S0	YTHDC2	3'-5' RNA helicase YTHDC2	8,10
Q6NXG1	ESRP1	Epithelial splicing regulatory protein 1	8,09
H0Y368	DPM1	Dolichol-phosphate mannosyltransferase subunit 1	8,08
Q92598	HSPH1	Heat shock protein 105 kDa	8,07
Q9Y5A9	YTHDF2	YTH domain-containing family protein 2	8,06
Q8N884	CGAS	Cyclic GMP-AMP synthase	8,03
Q9UJV9	DDX41	Probable ATP-dependent RNA helicase DDX41	7,99
J3KQJ1	SUMF2	Sulfatase modifying factor 2	7,99
P41252	IARS1	Isoleucine--tRNA ligase, cytoplasmic	7,97
P56192	MARS1	Methionine--tRNA ligase, cytoplasmic	7,94
O95758	PTBP3	Polypyrimidine tract-binding protein 3	7,94
Q12904	AIMP1	Aminoacyl tRNA synthase complex-interacting multifunctional protein 1	7,92
Q5T280	SPOUT1	Putative methyltransferase C9orf114	7,91
Q9UQ35	SRRM2	Serine/arginine repetitive matrix protein 2	7,90
O43615	TIMM44	Mitochondrial import inner membrane translocase subunit TIM44	7,87
A0A499FIZ0	WDR26	WD repeat domain 26	7,87
Q9UGP8	SEC63	Translocation protein SEC63 homolog	7,86
P21333	FLNA	Filamin-A	7,85
Q9UHD8	SEPTIN9	Septin-9	7,80
A0A8V8TQJ0	MSH6	DNA mismatch repair protein	7,79
K7ER00	FARSA	Phenylalanine--tRNA ligase alpha subunit	7,73
O43684	BUB3	Mitotic checkpoint protein BUB3	7,70
A0A8V8TM00	VPS45	Vacuolar protein sorting 45 homolog	7,69
A0A8I5KVU2	LARP4B	La ribonucleoprotein 4B	7,65
O94973	AP2A2	AP-2 complex subunit alpha-2	7,65
Q8N684	CPSF7	Cleavage and polyadenylation specificity factor subunit 7	7,64
P82650	MRPS22	Small ribosomal subunit protein mS22	7,63
H3BLV9	SRPK1	SRSF protein kinase 1 (Fragment)	7,61
Q96II8	LRCH3	DISP complex protein LRCH3	7,59
Q8NI36	WDR36	WD repeat-containing protein 36	7,58
O43290	SART1	U4/U6.U5 tri-snRNP-associated protein 1	7,57
Q5SSJ5	HP1BP3	Heterochromatin protein 1-binding protein 3	7,56
Q9ULS5	TMCC3	Transmembrane and coiled-coil domain protein 3	7,53
P29317	EPHA2	Ephrin type-A receptor 2	7,47
A0A0S2Z4G6	TPM1	Tropomyosin 1 (Fragment)	7,47
Q8IZH2	XRN1	5'-3' exoribonuclease 1	7,43
Q86UY5	FAM83A	Protein FAM83A	7,43
Q96CB9	NSUN4	5-methylcytosine rRNA methyltransferase NSUN4	7,42
Q16698	DECR1	2,4-dienoyl-CoA reductase [(3E)-enoyl-CoA-producing], mitochondrial	7,41
Q9Y6E2	BZW2	eIF5-mimic protein 1	7,38
Q16630	CPSF6	Cleavage and polyadenylation specificity factor subunit 6	7,38
A0A8Q3SIU1	RASAL2	RAS protein activator like 2	7,37
A0A7I2V4G0	HNRNPA3	Heterogeneous nuclear ribonucleoprotein A3	7,34
A0A0J9YWL0	CRYBG1	Crystallin beta-gamma domain containing 1	7,33
Q14137	BOP1	Ribosome biogenesis protein BOP1	7,33
Q9UKM7	MAN1B1	Endoplasmic reticulum mannosyl-oligosaccharide 1,2-alpha-mannosidase	7,31
Q9P0I2	EMC3	ER membrane protein complex subunit 3	7,31
Q15149	PLEC	Plectin	7,31
P08648	ITGA5	Integrin alpha-5	7,30
Q9BUF5	TUBB6	Tubulin beta-6 chain	7,28
Q5T0Z8	C6orf132	Uncharacterized protein C6orf132	7,28
P09497	CLTB	Clathrin light chain B	7,27
Q9H1I8	ASCC2	Activating signal cointegrator 1 complex subunit 2	7,22
Q96CS3	FAF2	FAS-associated factor 2	7,19
Q99614	TTC1	Tetratricopeptide repeat protein 1	7,17
Q9P0V3	SH3BP4	SH3 domain-binding protein 4	7,17
D6RBW1	EIF4E	Eukaryotic translation initiation factor 4E	7,15
O15355	PPM1G	Protein phosphatase 1G	7,14
O00567	NOP56	Nucleolar protein 56	7,11
O43741	PRKAB2	5'-AMP-activated protein kinase subunit beta-2	7,07
P07476	IVL	Involucrin	7,06
P11021	HSPA5	Endoplasmic reticulum chaperone BiP	7,05
E7ETY2	TCOF1	Treacle ribosome biogenesis factor 1	7,04
Q01780	EXOSC10	Exosome component 10	7,03
A0A6Q8PHA2	INF2	Inverted formin 2	7,03
Q14241	ELOA	Elongin-A	7,02
Q9BWF3	RBM4	RNA-binding protein 4	7,00
O43278	SPINT1	Kunitz-type protease inhibitor 1	6,99
Q9HAU0	PLEKHA5	Pleckstrin homology domain-containing family A member 5	6,95
Q15642	TRIP10	Cdc42-interacting protein 4	6,94
Q96I24	FUBP3	Far upstream element-binding protein 3	6,92
Q9UBB4	ATXN10	Ataxin-10	6,92
B5ME19	EIF3CL	Eukaryotic translation initiation factor 3 subunit C-like protein	6,91
Q13435	SF3B2	Splicing factor 3B subunit 2	6,88
Q53GQ0	HSD17B12	Very-long-chain 3-oxoacyl-CoA reductase	6,85
O75844	ZMPSTE24	CAAX prenyl protease 1 homolog	6,85
Q86X29	LSR	Lipolysis-stimulated lipoprotein receptor	6,81
Q15582	TGFBI	Transforming growth factor-beta-induced protein ig-h3	6,79
Q96CX2	KCTD12	BTB/POZ domain-containing protein KCTD12	6,77
P80723	BASP1	Brain acid soluble protein 1	6,75
Q8NC56	LEMD2	LEM domain-containing protein 2	6,75
O14744	PRMT5	Protein arginine N-methyltransferase 5	6,73
Q6P1M0	SLC27A4	Long-chain fatty acid transport protein 4	6,71
Q8TC12	RDH11	Retinol dehydrogenase 11	6,70
O95218	ZRANB2	Zinc finger Ran-binding domain-containing protein 2	6,68
Q14498	RBM39	RNA-binding protein 39	6,68
Q9UHR4	BAIAP2L1	Brain-specific angiogenesis inhibitor 1-associated protein 2-like protein 1	6,68
A0A0D9SF53	DDX3X	RNA helicase	6,67
F2Z2W7	TRMT2A	tRNA (uracil(54)-C(5))-methyltransferase	6,66
Q08J23	NSUN2	RNA cytosine C(5)-methyltransferase NSUN2	6,64
P61964	WDR5	WD repeat-containing protein 5	6,63
Q8TF74	WIPF2	WAS/WASL-interacting protein family member 2	6,63
Q5JTH9	RRP12	RRP12-like protein	6,58
O43464	HTRA2	Serine protease HTRA2, mitochondrial	6,58
Q9H329	EPB41L4B	Band 4.1-like protein 4B	6,58
Q02952	AKAP12	A-kinase anchor protein 12	6,54
Q99985	SEMA3C	Semaphorin-3C	6,54
Q53EP0	FNDC3B	Fibronectin type III domain-containing protein 3B	6,53
P78527	PRKDC	DNA-dependent protein kinase catalytic subunit	6,52
A0A7I2YQC0	NPM1	Nucleophosmin	6,52
Q96G21	IMP4	U3 small nucleolar ribonucleoprotein protein IMP4	6,52
Q9BRQ8	AIFM2	Ferroptosis suppressor protein 1	6,52
Q9BT40	INPP5K	Inositol polyphosphate 5-phosphatase K	6,52
Q96GQ7	DDX27	Probable ATP-dependent RNA helicase DDX27	6,50
P05121	SERPINE1	Plasminogen activator inhibitor 1	6,50
O14908	GIPC1	PDZ domain-containing protein GIPC1	6,48
A0A804HII9	ACTN1	Actinin alpha 1	6,44
G0XQ39	STIM1	STIM1L	6,44
Q9Y224	RTRAF	RNA transcription, translation and transport factor protein	6,43
P49915	GMPS	GMP synthase [glutamine-hydrolyzing]	6,42
Q13823	GNL2	Nucleolar GTP-binding protein 2	6,41
P23219	PTGS1	Prostaglandin G/H synthase 1	6,40
P62851	RPS25	Small ribosomal subunit protein eS25	6,40
Q9BYK8	HELZ2	Helicase with zinc finger domain 2	6,38
Q13753	LAMC2	Laminin subunit gamma-2	6,32
Q96IZ0	PAWR	PRKC apoptosis WT1 regulator protein	6,31
Q96A33	CCDC47	PAT complex subunit CCDC47	6,30
B0QZ18	CPNE1	Copine 1	6,29
I3L0E3	hCG_1984214	HCG1984214, isoform CRA_a	6,29
A0A7I2V3S3	EIF3E	Eukaryotic translation initiation factor 3 subunit E	6,29
A0A0G2JLB3	GBA1	Glucosylceramidase	6,29
A0AV96	RBM47	RNA-binding protein 47	6,29
O60716	CTNND1	Catenin delta-1	6,27
P61769	B2M	Beta-2-microglobulin	6,26
Q6PJ69	TRIM65	E3 ubiquitin-protein ligase TRIM65	6,24
Q15738	NSDHL	Sterol-4-alpha-carboxylate 3-dehydrogenase, decarboxylating	6,22
A0A087X2I1	PSMC6	Proteasome 26S subunit, ATPase 6	6,20
Q9Y624	F11R	Junctional adhesion molecule A	6,18
P05120	SERPINB2	Plasminogen activator inhibitor 2	6,13
Q8TDN6	BRIX1	Ribosome biogenesis protein BRX1 homolog	6,13
Q15437	SEC23B	Protein transport protein Sec23B	6,12
O75436	VPS26A	Vacuolar protein sorting-associated protein 26A	6,11
P11279	LAMP1	Lysosome-associated membrane glycoprotein 1	6,08
Q9UL25	RAB21	Ras-related protein Rab-21	6,06
P40938	RFC3	Replication factor C subunit 3	6,06
P62277	RPS13	Small ribosomal subunit protein uS15	6,00
Q9H936	SLC25A22	Mitochondrial glutamate carrier 1	5,98
Q99442	SEC62	Translocation protein SEC62	5,98
Q71RC2	LARP4	La-related protein 4	5,97
O43242	PSMD3	26S proteasome non-ATPase regulatory subunit 3	5,97
Q13162	PRDX4	Peroxiredoxin-4	5,96
P40937	RFC5	Replication factor C subunit 5	5,96
Q08174	PCDH1	Protocadherin-1	5,96
Q9BVI4	NOC4L	Nucleolar complex protein 4 homolog	5,95
P35998	PSMC2	26S proteasome regulatory subunit 7	5,95
A0A7I2YQF8	EIF3I	Eukaryotic translation initiation factor 3 subunit I	5,95
Q8WVX9	FAR1	Fatty acyl-CoA reductase 1	5,94
Q3ZCQ8	TIMM50	Mitochondrial import inner membrane translocase subunit TIM50	5,93
P61513	RPL37A	Large ribosomal subunit protein eL43	5,93
H7C5A2	EMC1	ER membrane protein complex subunit 1	5,92
Q9Y4E8	USP15	Ubiquitin carboxyl-terminal hydrolase 15	5,91
A0A8J8YWN6	CYB5R3	cytochrome-b5 reductase	5,91
D3DQV9	EIF4G2	Eukaryotic translation initiation factor 4 gamma 2 (Fragment)	5,91
Q96MG8	PCMTD1	Protein-L-isoaspartate O-methyltransferase domain-containing protein 1	5,91
D6RBZ0	HNRNPAB	Heterogeneous nuclear ribonucleoprotein A/B	5,89
A0A024QZP7	CDK1	Cell division cycle 2, G1 to S and G2 to M, isoform CRA_a	5,89
P49368	CCT3	T-complex protein 1 subunit gamma	5,88
Q7LDY3	TRMT10C	tRNA methyltransferase 10 homolog C	5,87
P00749	PLAU	Urokinase-type plasminogen activator	5,87
P49757	NUMB	Protein numb homolog	5,86
Q9BST9	RTKN	Rhotekin	5,86
Q6Q0C0	TRAF7	E3 ubiquitin-protein ligase TRAF7	5,84
E9PL17	CLP1	Polyribonucleotide 5'-hydroxyl-kinase Clp1	5,83
A0A087WZN1	IDH3B	Isocitrate dehydrogenase [NAD] subunit, mitochondrial	5,82
Q92896	GLG1	Golgi apparatus protein 1	5,81
Q96SQ9	CYP2S1	Cytochrome P450 2S1	5,81
Q14764	MVP	Major vault protein	5,81
O00442	RTCA	RNA 3'-terminal phosphate cyclase	5,81
Q6P158	DHX57	Putative ATP-dependent RNA helicase DHX57	5,81
A0A994J6W3	SPTAN1	Spectrin alpha, non-erythrocytic 1	5,80
Q9H223	EHD4	EH domain-containing protein 4	5,79
Q96JK2	DCAF5	DDB1- and CUL4-associated factor 5	5,77
A0A7P0T9U7	SF1	Splicing factor 1	5,77
Q96AQ6	PBXIP1	Pre-B-cell leukemia transcription factor-interacting protein 1	5,77
P52907	CAPZA1	F-actin-capping protein subunit alpha-1	5,74
Q6ZVM7	TOM1L2	TOM1-like protein 2	5,73
F8W727	RPL32	Ribosomal protein L32	5,71
Q13148	TARDBP	TAR DNA-binding protein 43	5,71
Q5VYS8	TUT7	Terminal uridylyltransferase 7	5,71
Q9Y263	PLAA	Phospholipase A-2-activating protein	5,70
Q969X5	ERGIC1	Endoplasmic reticulum-Golgi intermediate compartment protein 1	5,68
Q9UHQ9	CYB5R1	NADH-cytochrome b5 reductase 1	5,68
Q99720	SIGMAR1	Sigma non-opioid intracellular receptor 1	5,68
O43847	NRDC	Nardilysin	5,67
O00469	PLOD2	Procollagen-lysine,2-oxoglutarate 5-dioxygenase 2	5,66
P27482	CALML3	Calmodulin-like protein 3	5,65
O75369	FLNB	Filamin-B	5,65
P10253	GAA	Lysosomal alpha-glucosidase	5,63
O15270	SPTLC2	Serine palmitoyltransferase 2	5,60
J3KPS0	DNAJB12	DnaJ (Hsp40) homolog, subfamily B, member 12, isoform CRA_c	5,60
P04843	RPN1	Dolichyl-diphosphooligosaccharide--protein glycosyltransferase subunit 1	5,59
P27701	CD82	CD82 antigen	5,59
Q9NQC3	RTN4	Reticulon-4	5,59
P07204	THBD	Thrombomodulin	5,58
P58107	EPPK1	Epiplakin	5,58
A0A8V8TM76	TAP1	ABC-type antigen peptide transporter	5,58
Q99805	TM9SF2	Transmembrane 9 superfamily member 2	5,57
Q86W92	PPFIBP1	Liprin-beta-1	5,56
Q5JTV8	TOR1AIP1	Torsin-1A-interacting protein 1	5,55
Q969S8	HDAC10	Polyamine deacetylase HDAC10	5,54
P29590	PML	Protein PML	5,54
Q14165	MLEC	Malectin	5,53
P54819	AK2	Adenylate kinase 2, mitochondrial	5,53
Q9UHI6	DDX20	Probable ATP-dependent RNA helicase DDX20	5,53
P22087	FBL	rRNA 2'-O-methyltransferase fibrillarin	5,53
A0A0A0MTJ9	NCEH1	Neutral cholesterol ester hydrolase 1	5,52
P16615	ATP2A2	Sarcoplasmic/endoplasmic reticulum calcium ATPase 2	5,51
O60488	ACSL4	Long-chain-fatty-acid--CoA ligase 4	5,50
J3QRS3	MYL12A	Myosin light chain 12A	5,50
Q6DD88	ATL3	Atlastin-3	5,49
Q9UIQ6	LNPEP	Leucyl-cystinyl aminopeptidase	5,48
A0A2R8Y891	PFKM	ATP-dependent 6-phosphofructokinase	5,47
P11498	PC	Pyruvate carboxylase, mitochondrial	5,44
Q9Y5Q8	GTF3C5	General transcription factor 3C polypeptide 5	5,41
P06756	ITGAV	Integrin alpha-V	5,38
P09758	TACSTD2	Tumor-associated calcium signal transducer 2	5,37
Q13596	SNX1	Sorting nexin-1	5,37
A0A087WT44	HMOX2	heme oxygenase (biliverdin-producing)	5,32
Q9NUJ1	ABHD10	Palmitoyl-protein thioesterase ABHD10, mitochondrial	5,29
B1AK88	CAPZB	F-actin-capping protein subunit beta	5,28
A3KFL5	EXOSC2	Ribosomal RNA-processing protein 4	5,28
Q15397	PUM3	Pumilio homolog 3	5,27
Q9NYU2	UGGT1	UDP-glucose:glycoprotein glucosyltransferase 1	5,27
Q96PM9	ZNF385A	Zinc finger protein 385A	5,27
A0A0G2JIW1	HSPA1B	Heat shock 70 kDa protein 1B	5,27
P22695	UQCRC2	Cytochrome b-c1 complex subunit 2, mitochondrial	5,26
P11142	HSPA8	Heat shock cognate 71 kDa protein	5,24
P62861	FAU	Ubiquitin-like FUBI-ribosomal protein eS30 fusion protein	5,23
Q8IXI2	RHOT1	Mitochondrial Rho GTPase 1	5,21
G5EA30	CELF1	CUG triplet repeat, RNA binding protein 1, isoform CRA_c	5,19
P10412	H1-4	Histone H1.4	5,18
O60568	PLOD3	Multifunctional procollagen lysine hydroxylase and glycosyltransferase LH3	5,18
A0A087WUZ3	SPTBN1	Spectrin beta chain	5,17
Q14152	EIF3A	Eukaryotic translation initiation factor 3 subunit A	5,16
P35250	RFC2	Replication factor C subunit 2	5,16
Q7L5N7	LPCAT2	Lysophosphatidylcholine acyltransferase 2	5,15
P17480	UBTF	Nucleolar transcription factor 1	5,15
P50454	SERPINH1	Serpin H1	5,14
Q86UP2	KTN1	Kinectin	5,12
Q9BS26	ERP44	Endoplasmic reticulum resident protein 44	5,12
P04181	OAT	Ornithine aminotransferase, mitochondrial	5,11
O60313	OPA1	Dynamin-like 120 kDa protein, mitochondrial	5,11
P31689	DNAJA1	DnaJ homolog subfamily A member 1	5,11
A0A8V8TN34	SLC44A1	Choline transporter-like protein	5,11
Q5JPT2	SH3KBP1	SH3 domain containing kinase binding protein 1	5,11
Q9UMD9	COL17A1	Collagen alpha-1(XVII) chain	5,10
A0A8V8TRB3	PPP2CA	Serine/threonine-protein phosphatase	5,10
Q969V3	NCLN	BOS complex subunit NCLN	5,08
P54652	HSPA2	Heat shock-related 70 kDa protein 2	5,08
P99999	CYCS	Cytochrome c	5,08
A0A7I2V409	MARK3	non-specific serine/threonine protein kinase	5,06
P35625	TIMP3	Metalloproteinase inhibitor 3	5,02
Q13751	LAMB3	Laminin subunit beta-3	5,00
P13646	KRT13	Keratin, type I cytoskeletal 13	5,00
Q9H7Z7	PTGES2	Prostaglandin E synthase 2	5,00
P05387	RPLP2	Large ribosomal subunit protein P2	4,99
O15027	SEC16A	Protein transport protein Sec16A	4,97
Q99714	HSD17B10	3-hydroxyacyl-CoA dehydrogenase type-2	4,97
P61020	RAB5B	Ras-related protein Rab-5B	4,97
Q96GM8	TOE1	Target of EGR1 protein 1	4,97
P12956	XRCC6	X-ray repair cross-complementing protein 6	4,96
H0YHG0	DNAJC14	DnaJ homolog subfamily C member 14 (Fragment)	4,96
X6RAY8	MRPL4	Mitochondrial ribosomal protein L4	4,96
Q9UNF1	MAGED2	Melanoma-associated antigen D2	4,96
P31947	SFN	14-3-3 protein sigma	4,95
Q96G23	CERS2	Ceramide synthase 2	4,95
P62140	PPP1CB	Serine/threonine-protein phosphatase PP1-beta catalytic subunit	4,94
Q9Y639	NPTN	Neuroplastin	4,91
Q10471	GALNT2	Polypeptide N-acetylgalactosaminyltransferase 2	4,90
Q8NC51	SERBP1	SERPINE1 mRNA-binding protein 1	4,90
P21127	CDK11B	Cyclin-dependent kinase 11B	4,90
Q96HE7	ERO1A	ERO1-like protein alpha	4,88
O95159	ZFPL1	Zinc finger protein-like 1	4,88
Q08945	SSRP1	FACT complex subunit SSRP1	4,87
P22528	SPRR1B	Cornifin-B	4,86
Q9NZN4	EHD2	EH domain-containing protein 2	4,85
P04792	HSPB1	Heat shock protein beta-1	4,84
C9JMN9	PDIA4	Protein disulfide-isomerase	4,84
Q9Y295	DRG1	Developmentally-regulated GTP-binding protein 1	4,83
P62263	RPS14	Small ribosomal subunit protein uS11	4,83
Q8IUF8	RIOX2	Ribosomal oxygenase 2	4,83
P51659	HSD17B4	Peroxisomal multifunctional enzyme type 2	4,82
Q86UK7	ZNF598	E3 ubiquitin-protein ligase ZNF598	4,82
P61978	HNRNPK	Heterogeneous nuclear ribonucleoprotein K	4,79
P49411	TUFM	Elongation factor Tu, mitochondrial	4,79
O43660	PLRG1	Pleiotropic regulator 1	4,79
A0A804HL12	MYCBP2	RCR-type E3 ubiquitin transferase	4,79
Q14247	CTTN	Src substrate cortactin	4,77
Q9BQA1	WDR77	Methylosome protein WDR77	4,76
Q52LJ0	FAM98B	Protein FAM98B	4,76
Q9NUL3	STAU2	Double-stranded RNA-binding protein Staufen homolog 2	4,75
P82914	MRPS15	Small ribosomal subunit protein uS15m	4,75
Q9NSD9	FARSB	Phenylalanine--tRNA ligase beta subunit	4,74
P36542	ATP5F1C	ATP synthase subunit gamma, mitochondrial	4,74
A0A1B0GV77	FBXO31	F-box protein 31	4,73
O00391	QSOX1	Sulfhydryl oxidase 1	4,73
O95202	LETM1	Mitochondrial proton/calcium exchanger protein	4,72
P55884	EIF3B	Eukaryotic translation initiation factor 3 subunit B	4,72
P62993	GRB2	Growth factor receptor-bound protein 2	4,71
Q9BRR6	ADPGK	ADP-dependent glucokinase	4,71
E9PGZ1	CALD1	Caldesmon 1	4,68
P62899	RPL31	Large ribosomal subunit protein eL31	4,68
P62249	RPS16	Small ribosomal subunit protein uS9	4,68
E7EUU4	EIF4G1	Eukaryotic translation initiation factor 4 gamma 1	4,67
P14678	SNRPB	Small nuclear ribonucleoprotein-associated proteins B and B'	4,65
P56537	EIF6	Eukaryotic translation initiation factor 6	4,64
Q8NHP8	PLBD2	Putative phospholipase B-like 2	4,64
P47895	ALDH1A3	Retinaldehyde dehydrogenase 3	4,63
O95429	BAG4	BAG family molecular chaperone regulator 4	4,62
O00231	PSMD11	26S proteasome non-ATPase regulatory subunit 11	4,57
B0YIW6	ARCN1	Coatomer subunit delta	4,56
P09661	SNRPA1	U2 small nuclear ribonucleoprotein A'	4,54
A0A8V8TMY4	PFKP	ATP-dependent 6-phosphofructokinase	4,54
A0A8J9BZL8	GOLGA2	Golgin A2	4,53
Q93008	USP9X	Probable ubiquitin carboxyl-terminal hydrolase FAF-X	4,52
P61254	RPL26	Large ribosomal subunit protein uL24	4,52
Q14974	KPNB1	Importin subunit beta-1	4,51
A0A3B3ITW3	ABCD3	ATP binding cassette subfamily D member 3	4,48
P50416	CPT1A	Carnitine O-palmitoyltransferase 1, liver isoform	4,48
Q8IWB1	ITPRIP	Inositol 1,4,5-trisphosphate receptor-interacting protein	4,46
A0A0G2JN29	NOMO1	Nodal modulator 1	4,45
O15460	P4HA2	Prolyl 4-hydroxylase subunit alpha-2	4,45
A0A8I5QKQ8	SPTLC1	Serine palmitoyltransferase long chain base subunit 1	4,43
Q13308	PTK7	Inactive tyrosine-protein kinase 7	4,42
Q5T4S7	UBR4	E3 ubiquitin-protein ligase UBR4	4,38
Q5RKV6	EXOSC6	Exosome complex component MTR3	4,38
P05455	SSB	Lupus La protein	4,37
Q07960	ARHGAP1	Rho GTPase-activating protein 1	4,35
Q9NY93	DDX56	Probable ATP-dependent RNA helicase DDX56	4,33
P17931	LGALS3	Galectin-3	4,31
P61353	RPL27	Large ribosomal subunit protein eL27	4,31
P13473	LAMP2	Lysosome-associated membrane glycoprotein 2	4,30
Q15366	PCBP2	Poly(rC)-binding protein 2	4,28
H3BQK9	MACF1	Microtubule actin crosslinking factor 1	4,25
Q96N66	MBOAT7	Lysophospholipid acyltransferase 7	4,21
Q9NX58	LYAR	Cell growth-regulating nucleolar protein	4,21
P30048	PRDX3	Thioredoxin-dependent peroxide reductase, mitochondrial	4,20
Q9Y277	VDAC3	Voltage-dependent anion-selective channel protein 3	4,20
Q12931	TRAP1	Heat shock protein 75 kDa, mitochondrial	4,20
Q9NRG9	AAAS	Aladin	4,17
P35251	RFC1	Replication factor C subunit 1	4,17
P60842	EIF4A1	Eukaryotic initiation factor 4A-I	4,16
Q92520	FAM3C	Protein FAM3C	4,16
P50991	CCT4	T-complex protein 1 subunit delta	4,14
A0A804HI25	DHCR7	7-dehydrocholesterol reductase	4,14
Q96N67	DOCK7	Dedicator of cytokinesis protein 7	4,10
P23634	ATP2B4	Plasma membrane calcium-transporting ATPase 4	4,09
P17812	CTPS1	CTP synthase 1	4,05
O43681	GET3	ATPase GET3	4,04
I3L4C2	BAIAP2	Brain-specific angiogenesis inhibitor 1-associated protein 2	4,02
A0A6Q8PFY7	TFG	Trafficking from ER to golgi regulator	4,02
P67936	TPM4	Tropomyosin alpha-4 chain	4,01
A0A669KBH4	CUL7	Cullin 7	4,01
P17858	PFKL	ATP-dependent 6-phosphofructokinase, liver type	4,01
P08236	GUSB	Beta-glucuronidase	4,01
J3KTL2	SRSF1	Serine/arginine-rich splicing factor 1	4,00
Q96C19	EFHD2	EF-hand domain-containing protein D2	4,00
A0A087WUS3	MAGT1	Magnesium transporter 1	3,99
A0A8V8TQ44	PSMD8	26S proteasome non-ATPase regulatory subunit 8	3,99
P26572	MGAT1	Alpha-1,3-mannosyl-glycoprotein 2-beta-N-acetylglucosaminyltransferase	3,98
Q14257	RCN2	Reticulocalbin-2	3,98
P13639	EEF2	Elongation factor 2	3,96
Q99873	PRMT1	Protein arginine N-methyltransferase 1	3,95
Q07021	C1QBP	Complement component 1 Q subcomponent-binding protein, mitochondrial	3,95
P31431	SDC4	Syndecan-4	3,95
O15235	MRPS12	Small ribosomal subunit protein uS12m	3,94
O14734	ACOT8	Acyl-coenzyme A thioesterase 8	3,94
P25398	RPS12	Small ribosomal subunit protein eS12	3,93
B0QYD3	APOBEC3B	Apolipoprotein B mRNA editing enzyme catalytic subunit 3B	3,92
Q6P2E9	EDC4	Enhancer of mRNA-decapping protein 4	3,91
P11234	RALB	Ras-related protein Ral-B	3,91
P09429	HMGB1	High mobility group protein B1	3,91
Q12846	STX4	Syntaxin-4	3,91
P39019	RPS19	Small ribosomal subunit protein eS19	3,90
P27708	CAD	CAD protein	3,90
A0A7P0TAE9	CANX	Calnexin	3,88
P51636	CAV2	Caveolin-2	3,88
P51572	BCAP31	B-cell receptor-associated protein 31	3,87
P09543	CNP	2',3'-cyclic-nucleotide 3'-phosphodiesterase	3,87
Q9UJS0	SLC25A13	Electrogenic aspartate/glutamate antiporter SLC25A13, mitochondrial	3,86
A0A994J5J1	RPN2	Dolichyl-diphosphooligosaccharide--protein glycosyltransferase subunit 2	3,86
Q9H444	CHMP4B	Charged multivesicular body protein 4b	3,86
Q7L576	CYFIP1	Cytoplasmic FMR1-interacting protein 1	3,85
Q8WUK0	PTPMT1	Phosphatidylglycerophosphatase and protein-tyrosine phosphatase 1	3,85
Q99470	SDF2	Stromal cell-derived factor 2	3,82
P02538	KRT6A	Keratin, type II cytoskeletal 6A	3,82
P23284	PPIB	Peptidyl-prolyl cis-trans isomerase B	3,81
Q9H0J9	PARP12	Protein mono-ADP-ribosyltransferase PARP12	3,81
O43395	PRPF3	U4/U6 small nuclear ribonucleoprotein Prp3	3,80
P07737	PFN1	Profilin-1	3,79
Q9H5V8	CDCP1	CUB domain-containing protein 1	3,78
P00492	HPRT1	Hypoxanthine-guanine phosphoribosyltransferase	3,78
Q9Y5M8	SRPRB	Signal recognition particle receptor subunit beta	3,78
K7EQD6	NSF	Vesicle-fusing ATPase	3,77
A0A8V8TPC7	ERBIN	Erbb2 interacting protein	3,76
P55072	VCP	Transitional endoplasmic reticulum ATPase	3,76
Q8IXB1	DNAJC10	DnaJ homolog subfamily C member 10	3,75
P80303	NUCB2	Nucleobindin-2	3,74
P63151	PPP2R2A	Serine/threonine-protein phosphatase 2A 55 kDa regulatory subunit B alpha isoform	3,74
A0A7P0T871	ACAD9	Acyl-CoA dehydrogenase family member 9	3,74
O95817	BAG3	BAG family molecular chaperone regulator 3	3,73
Q6YN16	HSDL2	Hydroxysteroid dehydrogenase-like protein 2	3,72
P42167	TMPO	Lamina-associated polypeptide 2, isoforms beta/gamma	3,71
P01889	HLA-B	HLA class I histocompatibility antigen, B alpha chain	3,70
Q7L3T8	PARS2	Probable proline--tRNA ligase, mitochondrial	3,68
P25789	PSMA4	Proteasome subunit alpha type-4	3,68
Q9Y266	NUDC	Nuclear migration protein nudC	3,68
P40227	CCT6A	T-complex protein 1 subunit zeta	3,68
Q08380	LGALS3BP	Galectin-3-binding protein	3,67
Q9BUP3	HTATIP2	Oxidoreductase HTATIP2	3,67
Q9Y5Y5	PEX16	Peroxisomal membrane protein PEX16	3,67
S4R369	MRPL37	Mitochondrial ribosomal protein L37	3,66
Q13510	ASAH1	Acid ceramidase	3,65
Q14683	SMC1A	Structural maintenance of chromosomes protein 1A	3,64
P0DUB6	AMY1A	Alpha-amylase 1A	3,64
O15127	SCAMP2	Secretory carrier-associated membrane protein 2	3,63
Q53H12	AGK	Acylglycerol kinase, mitochondrial	3,62
Q03468	ERCC6	DNA excision repair protein ERCC-6	3,62
P46782	RPS5	Small ribosomal subunit protein uS7	3,62
O43707	ACTN4	Alpha-actinin-4	3,61
Q14697	GANAB	Neutral alpha-glucosidase AB	3,61
Q96QE5	TEFM	Transcription elongation factor, mitochondrial	3,60
Q15293	RCN1	Reticulocalbin-1	3,60
O15427	SLC16A3	Monocarboxylate transporter 4	3,60
P09622	DLD	Dihydrolipoyl dehydrogenase, mitochondrial	3,58
P34897	SHMT2	Serine hydroxymethyltransferase, mitochondrial	3,57
P62633	CNBP	CCHC-type zinc finger nucleic acid binding protein	3,56
O75052	NOS1AP	Carboxyl-terminal PDZ ligand of neuronal nitric oxide synthase protein	3,55
A0A0C4DG49	PVR	PVR cell adhesion molecule	3,55
P78346	RPP30	Ribonuclease P protein subunit p30	3,55
Q9BT09	CNPY3	Protein canopy homolog 3	3,54
Q16891	IMMT	MICOS complex subunit MIC60	3,54
P30044	PRDX5	Peroxiredoxin-5, mitochondrial	3,51
C9J9C1	PPP2R1A	Protein phosphatase 2 scaffold subunit Aalpha	3,51
O15400	STX7	Syntaxin-7	3,50
A0A8V8TLA4	RPSA	40S ribosomal protein SA	3,50
P50990	CCT8	T-complex protein 1 subunit theta	3,49
P36551	CPOX	Oxygen-dependent coproporphyrinogen-III oxidase, mitochondrial	3,48
O43809	NUDT21	Cleavage and polyadenylation specificity factor subunit 5	3,47
Q13200	PSMD2	26S proteasome non-ATPase regulatory subunit 2	3,44
Q9UJ70	NAGK	N-acetyl-D-glucosamine kinase	3,44
Q96P11	NSUN5	28S rRNA (cytosine-C(5))-methyltransferase	3,44
A0A8V8TLM6	TFRC	Transferrin receptor protein 1	3,42
Q9H307	PNN	Pinin	3,42
Q08722	CD47	Leukocyte surface antigen CD47	3,41
P62081	RPS7	Small ribosomal subunit protein eS7	3,41
P13489	RNH1	Ribonuclease inhibitor	3,40
E5RHG6	TBCA	Tubulin-specific chaperone A	3,39
Q9Y383	LUC7L2	Putative RNA-binding protein Luc7-like 2	3,38
P55209	NAP1L1	Nucleosome assembly protein 1-like 1	3,37
A0A0C4DFV9	SET	SET nuclear proto-oncogene	3,36
Q06210	GFPT1	Glutamine--fructose-6-phosphate aminotransferase [isomerizing] 1	3,36
Q9Y678	COPG1	Coatomer subunit gamma-1	3,36
O14773	TPP1	Tripeptidyl-peptidase 1	3,32
O15260	SURF4	Surfeit locus protein 4	3,32
Q96HY6	DDRGK1	DDRGK domain-containing protein 1	3,32
Q6UXN9	WDR82	WD repeat-containing protein 82	3,30
Q92930	RAB8B	Ras-related protein Rab-8B	3,30
P16435	POR	NADPH--cytochrome P450 reductase	3,29
O95292	VAPB	Vesicle-associated membrane protein-associated protein B/C	3,26
P78371	CCT2	T-complex protein 1 subunit beta	3,23
P60953	CDC42	Cell division control protein 42 homolog	3,23
Q00587	CDC42EP1	Cdc42 effector protein 1	3,23
P50402	EMD	Emerin	3,22
Q99536	VAT1	Synaptic vesicle membrane protein VAT-1 homolog	3,21
Q15024	EXOSC7	Exosome complex component RRP42	3,19
P68371	TUBB4B	Tubulin beta-4B chain	3,19
O94874	UFL1	E3 UFM1-protein ligase 1	3,19
O15126	SCAMP1	Secretory carrier-associated membrane protein 1	3,19
A0A8I5KZB3	RAB3GAP2	RAB3 GTPase activating non-catalytic protein subunit 2	3,18
P54920	NAPA	Alpha-soluble NSF attachment protein	3,18
O75323	NIPSNAP2	Protein NipSnap homolog 2	3,17
Q9UKS6	PACSIN3	Protein kinase C and casein kinase substrate in neurons protein 3	3,16
A0A994J6K9	NCKAP1	NCK associated protein 1	3,16
Q14240	EIF4A2	Eukaryotic initiation factor 4A-II	3,15
A0A7P0S5H5	RPS20	40S ribosomal protein S20 (Fragment)	3,15
Q6NUK1	SLC25A24	Mitochondrial adenyl nucleotide antiporter SLC25A24	3,15
O00458	IFRD1	Interferon-related developmental regulator 1	3,14
Q96IF1	AJUBA	LIM domain-containing protein ajuba	3,14
P33992	MCM5	DNA replication licensing factor MCM5	3,14
Q96JJ7	TMX3	Protein disulfide-isomerase TMX3	3,13
A0A0J9YXF2	PON2	Paraoxonase	3,12
Q8WV24	PHLDA1	Pleckstrin homology-like domain family A member 1	3,11
Q96QC0	PPP1R10	Serine/threonine-protein phosphatase 1 regulatory subunit 10	3,11
Q9UHB9	SRP68	Signal recognition particle subunit SRP68	3,10
Q99733	NAP1L4	Nucleosome assembly protein 1-like 4	3,08
O60832	DKC1	H/ACA ribonucleoprotein complex subunit DKC1	3,07
P20645	M6PR	Cation-dependent mannose-6-phosphate receptor	3,06
O60563	CCNT1	Cyclin-T1	3,05
Q9NXC5	MIOS	GATOR complex protein MIOS	3,04
Q9BZG1	RAB34	Ras-related protein Rab-34	3,04
E9PHZ5	CTSB	Cathepsin B	3,03
P54886	ALDH18A1	Delta-1-pyrroline-5-carboxylate synthase	3,02
A0A2R8Y5A3	CTNNB1	Catenin beta 1	3,02
P52789	HK2	Hexokinase-2	3,02
A0A2R8YG02	MSH2	DNA mismatch repair protein	3,01
P17301	ITGA2	Integrin alpha-2	3,00
Q14677	CLINT1	Clathrin interactor 1	2,99
P31040	SDHA	Succinate dehydrogenase [ubiquinone] flavoprotein subunit, mitochondrial	2,99
A8MUS3	RPL23A	Ribosomal protein L23a	2,99
Q9UQ80	PA2G4	Proliferation-associated protein 2G4	2,99
P07602	PSAP	Prosaposin	2,98
Q14684	RRP1B	Ribosomal RNA processing protein 1 homolog B	2,97
Q9HDC9	APMAP	Adipocyte plasma membrane-associated protein	2,97
Q9P0L0	VAPA	Vesicle-associated membrane protein-associated protein A	2,96
Q9UKY7	CDV3	Protein CDV3 homolog	2,95
P16070	CD44	CD44 antigen	2,95
M0R0C6	KHSRP	KH-type splicing regulatory protein	2,93
P06703	S100A6	Protein S100-A6	2,92
Q9Y265	RUVBL1	RuvB-like 1	2,92
Q1KMD3	HNRNPUL2	Heterogeneous nuclear ribonucleoprotein U-like protein 2	2,91
Q96EV2	RBM33	RNA-binding protein 33	2,91
A0A8V8TP06	SFXN3	Sideroflexin	2,90
P49720	PSMB3	Proteasome subunit beta type-3	2,88
B3KS98	EIF3H	Eukaryotic translation initiation factor 3 subunit H	2,87
O94876	TMCC1	Transmembrane and coiled-coil domains protein 1	2,86
Q92597	NDRG1	Protein NDRG1	2,86
Q9Y3D9	MRPS23	Small ribosomal subunit protein mS23	2,85
Q14847	LASP1	LIM and SH3 domain protein 1	2,84
P20591	MX1	Interferon-induced GTP-binding protein Mx1	2,84
P57772	EEFSEC	Selenocysteine-specific elongation factor	2,84
Q12891	HYAL2	Hyaluronidase-2	2,84
P30101	PDIA3	Protein disulfide-isomerase A3	2,84
Q13838	DDX39B	Spliceosome RNA helicase DDX39B	2,83
P06737	PYGL	Glycogen phosphorylase, liver form	2,83
K7ELC2	RPS15	40S ribosomal protein S15	2,82
Q9Y5Q9	GTF3C3	General transcription factor 3C polypeptide 3	2,82
Q9UBN7	HDAC6	Histone deacetylase 6	2,82
P21397	MAOA	Amine oxidase [flavin-containing] A	2,82
P35613	BSG	Basigin	2,81
Q9HD33	MRPL47	Large ribosomal subunit protein uL29m	2,79
Q6RW13	AGTRAP	Type-1 angiotensin II receptor-associated protein	2,79
P25786	PSMA1	Proteasome subunit alpha type-1	2,79
Q8NBJ5	COLGALT1	Procollagen galactosyltransferase 1	2,78
E7EX17	EIF4B	Eukaryotic translation initiation factor 4B	2,78
A0A8V8TMZ5	SEC61A1	SEC61 translocon subunit alpha 1	2,78
P62136	PPP1CA	Serine/threonine-protein phosphatase PP1-alpha catalytic subunit	2,77
Q92542	NCSTN	Nicastrin	2,77
A0A1W2PRS1	SCARB2	Scavenger receptor class B member 2 (Fragment)	2,77
A0A2R8YD14	RPS24	40S ribosomal protein S24	2,77
A0A0U1RRH7	H2A	Histone H2A	2,77
O94905	ERLIN2	Erlin-2	2,75
Q5SRN5	HLA-A	Major histocompatibility complex, class I, A	2,74
Q9H3P7	ACBD3	Golgi resident protein GCP60	2,73
Q9ULT8	HECTD1	E3 ubiquitin-protein ligase HECTD1	2,73
F8WCF6	ARPC4-TTLL3	Actin-related protein 2/3 complex subunit 4	2,72
O00161	SNAP23	Synaptosomal-associated protein 23	2,71
H0Y8C6	IPO5	Importin 5 (Fragment)	2,71
Q04695	KRT17	Keratin, type I cytoskeletal 17	2,70
A0A994J3Z2	GPAA1	Glycosylphosphatidylinositol anchor attachment 1	2,70
P30040	ERP29	Endoplasmic reticulum resident protein 29	2,70
P29350	PTPN6	Tyrosine-protein phosphatase non-receptor type 6	2,70
Q86Y82	STX12	Syntaxin-12	2,70
P55145	MANF	Mesencephalic astrocyte-derived neurotrophic factor	2,70
Q86VP6	CAND1	Cullin-associated NEDD8-dissociated protein 1	2,69
E9PL19	PUF60	Poly(U)-binding-splicing factor PUF60	2,68
P25686	DNAJB2	DnaJ homolog subfamily B member 2	2,68
Q96NY8	NECTIN4	Nectin-4	2,68
Q9Y230	RUVBL2	RuvB-like 2	2,67
O14980	XPO1	Exportin-1	2,67
F8VVM2	SLC25A3	Phosphate carrier protein, mitochondrial	2,66
P07437	TUBB	Tubulin beta chain	2,65
P55081	MFAP1	Microfibrillar-associated protein 1	2,64
P17980	PSMC3	26S proteasome regulatory subunit 6A	2,64
Q01650	SLC7A5	Large neutral amino acids transporter small subunit 1	2,64
P01008	SERPINC1	Antithrombin-III	2,63
P12532	CKMT1A	Creatine kinase U-type, mitochondrial	2,63
P17987	TCP1	T-complex protein 1 subunit alpha	2,61
Q8WW12	PCNP	PEST proteolytic signal-containing nuclear protein	2,61
P02794	FTH1	Ferritin heavy chain	2,59
B4DUC8	MTAP	S-methyl-5'-thioadenosine phosphorylase	2,58
P68871	HBB	Hemoglobin subunit beta	2,58
E9PAV3	NACA	Nascent polypeptide-associated complex subunit alpha, muscle-specific form	2,57
P29372	MPG	DNA-3-methyladenine glycosylase	2,56
Q13217	DNAJC3	DnaJ homolog subfamily C member 3	2,56
Q9BTV4	TMEM43	Transmembrane protein 43	2,55
P00533	EGFR	Epidermal growth factor receptor	2,55
P19012	KRT15	Keratin, type I cytoskeletal 15	2,54
P36957	DLST	Dihydrolipoyllysine-residue succinyltransferase component of 2-oxoglutarate dehydrogenase complex, mitochondrial	2,53
D6RE79	MFSD10	Major facilitator superfamily domain containing 10	2,53
P04179	SOD2	Superoxide dismutase [Mn], mitochondrial	2,53
O00515	LAD1	Ladinin-1	2,52
O75821	EIF3G	Eukaryotic translation initiation factor 3 subunit G	2,49
E7ESZ7	NDUFA10	NADH dehydrogenase [ubiquinone] 1 alpha subcomplex subunit 10, mitochondrial	2,48
Q8TER5	ARHGEF40	Rho guanine nucleotide exchange factor 40	2,48
Q15365	PCBP1	Poly(rC)-binding protein 1	2,48
Q9UBS4	DNAJB11	DnaJ homolog subfamily B member 11	2,47
H0YKD8	RPL28	60S ribosomal protein L28	2,47
A6NFX8	NUDT5	Nudix hydrolase 5	2,47
A0A994J749	IMPDH	Inosine-5'-monophosphate dehydrogenase	2,46
Q9Y2X3	NOP58	Nucleolar protein 58	2,46
E9PK25	CFL1	Cofilin 1	2,46
C9J0Y3	GOLGA4	Golgin A4	2,44
O75381	PEX14	Peroxisomal membrane protein PEX14	2,43
Q7Z2K6	ERMP1	Endoplasmic reticulum metallopeptidase 1	2,42
P54727	RAD23B	UV excision repair protein RAD23 homolog B	2,42
H0Y5K5	ERGIC3	Endoplasmic reticulum-Golgi intermediate compartment protein (Fragment)	2,42
O14745	NHERF1	Na(+)/H(+) exchange regulatory cofactor NHE-RF1	2,40
Q9BPW8	NIPSNAP1	Protein NipSnap homolog 1	2,39
Q13045	FLII	Protein flightless-1 homolog	2,38
Q9HB71	CACYBP	Calcyclin-binding protein	2,38
Q8TEQ6	GEMIN5	Gem-associated protein 5	2,36
P07910	HNRNPC	Heterogeneous nuclear ribonucleoproteins C1/C2	2,36
P26038	MSN	Moesin	2,36
P61026	RAB10	Ras-related protein Rab-10	2,35
E7EQZ4	SMN1	Survival motor neuron protein	2,35
P26006	ITGA3	Integrin alpha-3	2,34
Q7Z434	MAVS	Mitochondrial antiviral-signaling protein	2,34
P27797	CALR	Calreticulin	2,34
P46109	CRKL	Crk-like protein	2,32
Q9NX40	OCIAD1	OCIA domain-containing protein 1	2,31
P55060	CSE1L	Exportin-2	2,31
A0A0A0MQX8	MBNL1	Muscleblind like splicing regulator 1	2,30
Q15691	MAPRE1	Microtubule-associated protein RP/EB family member 1	2,30
P04083	ANXA1	Annexin A1	2,29
P39656	DDOST	Dolichyl-diphosphooligosaccharide--protein glycosyltransferase 48 kDa subunit	2,28
P07384	CAPN1	Calpain-1 catalytic subunit	2,28
A0A7P0TAT8	HSP90B1	Heat shock protein 90 beta family member 1	2,27
O14672	ADAM10	Disintegrin and metalloproteinase domain-containing protein 10	2,27
C9JH19	CTSD	Cathepsin D	2,26
Q9NR28	DIABLO	Diablo IAP-binding mitochondrial protein	2,26
A0A7I2YQG7	NDUFS2	NADH dehydrogenase [ubiquinone] iron-sulfur protein 2, mitochondrial	2,26
P12830	CDH1	Cadherin-1	2,26
O43852	CALU	Calumenin	2,25
P26583	HMGB2	High mobility group protein B2	2,25
Q06323	PSME1	Proteasome activator complex subunit 1	2,25
O00743	PPP6C	Serine/threonine-protein phosphatase 6 catalytic subunit	2,25
Q9Y2S7	POLDIP2	Polymerase delta-interacting protein 2	2,24
Q01844	EWSR1	RNA-binding protein EWS	2,24
P05023	ATP1A1	Sodium/potassium-transporting ATPase subunit alpha-1	2,23
Q13243	SRSF5	Serine/arginine-rich splicing factor 5	2,23
P18031	PTPN1	Tyrosine-protein phosphatase non-receptor type 1	2,21
O14802	POLR3A	DNA-directed RNA polymerase III subunit RPC1	2,20
P43304	GPD2	Glycerol-3-phosphate dehydrogenase, mitochondrial	2,20
P62269	RPS18	Small ribosomal subunit protein uS13	2,20
Q9NQR4	NIT2	Omega-amidase NIT2	2,19
Q8NBP0	TTC13	Tetratricopeptide repeat protein 13	2,19
J3KQE5	RAN	GTP-binding nuclear protein Ran (Fragment)	2,18
P45880	VDAC2	Voltage-dependent anion-selective channel protein 2	2,17
Q9HAV7	GRPEL1	GrpE protein homolog 1, mitochondrial	2,17
P21796	VDAC1	Voltage-dependent anion-selective channel protein 1	2,16
P49257	LMAN1	Protein ERGIC-53	2,15
Q99816	TSG101	Tumor susceptibility gene 101 protein	2,15
A0A7P0Z4P5	SLC3A2	Solute carrier family 3 member 2	2,15
P62857	RPS28	Small ribosomal subunit protein eS28	2,15
P17900	GM2A	Ganglioside GM2 activator	2,15
P13995	MTHFD2	Bifunctional methylenetetrahydrofolate dehydrogenase/cyclohydrolase, mitochondrial	2,14
O76094	SRP72	Signal recognition particle subunit SRP72	2,14
P08574	CYC1	Cytochrome c1, heme protein, mitochondrial	2,14
C9JSZ1	FUBP1	Far upstream element binding protein 1	2,12
P40926	MDH2	Malate dehydrogenase, mitochondrial	2,12
G8JLB6	HNRNPH1	Heterogeneous nuclear ribonucleoprotein H1	2,11
Q08209	PPP3CA	Protein phosphatase 3 catalytic subunit alpha	2,11
P57729	RAB38	Ras-related protein Rab-38	2,10
P60468	SEC61B	Protein transport protein Sec61 subunit beta	2,09
Q58FF6	HSP90AB4P	Putative heat shock protein HSP 90-beta 4	2,08
Q96HA8	NTAQ1	Protein N-terminal glutamine amidohydrolase	2,08
O60884	DNAJA2	DnaJ homolog subfamily A member 2	2,07
O95373	IPO7	Importin-7	2,06
Q96D15	RCN3	Reticulocalbin-3	2,06
P49721	PSMB2	Proteasome subunit beta type-2	2,06
P05109	S100A8	Protein S100-A8	2,06
A0A494C039	HYOU1	Hypoxia up-regulated 1	2,06
P12004	PCNA	Proliferating cell nuclear antigen	2,06
P63244	RACK1	Small ribosomal subunit protein RACK1	2,04
P25705	ATP5F1A	ATP synthase subunit alpha, mitochondrial	2,03
P46977	STT3A	Dolichyl-diphosphooligosaccharide--protein glycosyltransferase subunit STT3A	2,02
F5GXT1	CD9	Tetraspanin	2,02
K7ELL7	PRKCSH	Glucosidase 2 subunit beta	2,02
E7EMB3	CALM2	Calmodulin 2	2,01

Supplemental table 3 : Summary table of proteins present in A431-Tks5-GFP cells seeded on collagen identified by mass spectrometry analysis of Tks5 interactome

Accession	Gene Name	Description	Abundance Ratio: (TKS5) / (Control)
Q5TCZ1	SH3PXD2A	SH3 and PX domain-containing protein 2A	614,03
P18124	RPL7	Large ribosomal subunit protein uL30	81,80
A0A8Q3SIY9	AFAP1L2	Actin filament associated protein 1 like 2	73,20
Q13444	ADAM15	Disintegrin and metalloproteinase domain-containing protein 15	69,28
P05388	RPLP0	Large ribosomal subunit protein uL10	59,05
P62241	RPS8	Small ribosomal subunit protein eS8	57,90
P46777	RPL5	Large ribosomal subunit protein uL18	55,49
P62701	RPS4X	Small ribosomal subunit protein eS4, X isoform	50,19
Q00839	HNRNPU	Heterogeneous nuclear ribonucleoprotein U	48,73
P26373	RPL13	Large ribosomal subunit protein eL13	43,96
P36578	RPL4	Large ribosomal subunit protein uL4	43,48
P61981	YWHAG	14-3-3 protein gamma	42,73
P54619	PRKAG1	5'-AMP-activated protein kinase subunit gamma-1	42,71
P62753	RPS6	Small ribosomal subunit protein eS6	42,61
P50914	RPL14	Large ribosomal subunit protein eL14	42,51
P62917	RPL8	Large ribosomal subunit protein uL2	41,90
P62424	RPL7A	Large ribosomal subunit protein eL8	41,54
O75569	PRKRA	Interferon-inducible double-stranded RNA-dependent protein kinase activator A	36,14
P61247	RPS3A	Small ribosomal subunit protein eS1	35,80
J3QQ67	RPL18	Ribosomal protein L18 (Fragment)	34,58
P61313	RPL15	Large ribosomal subunit protein eL15	34,55
Q12965	MYO1E	Unconventional myosin-Ie	34,38
P39023	RPL3	Large ribosomal subunit protein uL3	33,86
Q02878	RPL6	Large ribosomal subunit protein eL6	32,68
Q6NXG1	ESRP1	Epithelial splicing regulatory protein 1	30,92
Q14103	HNRNPD	Heterogeneous nuclear ribonucleoprotein D0	30,20
F8W930	IGF2BP2	Insulin like growth factor 2 mRNA binding protein 2	29,11
P62854	RPS26	Small ribosomal subunit protein eS26	28,67
Q02543	RPL18A	Large ribosomal subunit protein eL20	28,64
Q9BQ67	GRWD1	Glutamate-rich WD repeat-containing protein 1	28,52
O75915	ARL6IP5	PRA1 family protein 3	28,43
Q9Y305	ACOT9	Acyl-coenzyme A thioesterase 9, mitochondrial	28,20
B7ZM99	MTHFD1L	formate--tetrahydrofolate ligase	27,93
Q9H0U9	TSPYL1	Testis-specific Y-encoded-like protein 1	27,68
Q9Y676	MRPS18B	Small ribosomal subunit protein mS40	27,62
P48735	IDH2	Isocitrate dehydrogenase [NADP], mitochondrial	27,53
P49207	RPL34	Large ribosomal subunit protein eL34	26,89
Q16787	LAMA3	Laminin subunit alpha-3	26,66
O15127	SCAMP2	Secretory carrier-associated membrane protein 2	26,34
Q13131	PRKAA1	5'-AMP-activated protein kinase catalytic subunit alpha-1	26,11
Q9ULV4	CORO1C	Coronin-1C	25,09
O95793	STAU1	Double-stranded RNA-binding protein Staufen homolog 1	24,92
Q12905	ILF2	Interleukin enhancer-binding factor 2	24,51
P57678	GEMIN4	Gem-associated protein 4	24,35
A0A0U1RRM4	PTBP1	Polypyrimidine tract-binding protein 1	24,28
Q9Y446	PKP3	Plakophilin-3	24,27
A0A6Q8PHA2	INF2	Inverted formin 2	23,80
Q9H013	ADAM19	Disintegrin and metalloproteinase domain-containing protein 19	23,69
P53621	COPA	Coatomer subunit alpha	23,57
Q9NVN8	GNL3L	Guanine nucleotide-binding protein-like 3-like protein	23,29
Q8NHH9	ATL2	Atlastin-2	23,18
P61160	ACTR2	Actin-related protein 2	22,87
Q9NYL9	TMOD3	Tropomodulin-3	22,48
P01834	IGKC	Immunoglobulin kappa constant	22,19
P02751	FN1	Fibronectin	21,87
P48651	PTDSS1	Phosphatidylserine synthase 1	21,79
P46063	RECQL	ATP-dependent DNA helicase Q1	21,54
Q8NCA5	FAM98A	Protein FAM98A	21,51
P35914	HMGCL	Hydroxymethylglutaryl-CoA lyase, mitochondrial	21,02
Q9Y478	PRKAB1	5'-AMP-activated protein kinase subunit beta-1	20,72
Q13724	MOGS	Mannosyl-oligosaccharide glucosidase	20,60
P62906	RPL10A	Large ribosomal subunit protein uL1	20,18
A0A5F9ZHN7	SACM1L	SAC1 like phosphatidylinositide phosphatase	20,12
P15880	RPS2	Small ribosomal subunit protein uS5	20,00
P34932	HSPA4	Heat shock 70 kDa protein 4	19,84
P07996	THBS1	Thrombospondin-1	19,62
P62913	RPL11	Large ribosomal subunit protein uL5	19,62
Q13310	PABPC4	Polyadenylate-binding protein 4	19,60
E9PC69	MARK2	non-specific serine/threonine protein kinase	19,58
Q9NYU2	UGGT1	UDP-glucose:glycoprotein glucosyltransferase 1	19,55
P52272	HNRNPM	Heterogeneous nuclear ribonucleoprotein M	19,46
P98174	FGD1	FYVE, RhoGEF and PH domain-containing protein 1	19,45
A0A8V8TQ35	FNBP1	Formin binding protein 1	19,29
A0A8V8TMQ1	FXR1	RNA-binding protein FXR1	19,03
A0A5H1ZRQ2	DDX17	RNA helicase	18,93
Q9HAN9	NMNAT1	Nicotinamide/nicotinic acid mononucleotide adenylyltransferase 1	18,91
A0A0A0MSV9	TAPBP	Tapasin	18,76
P11387	TOP1	DNA topoisomerase 1	18,75
P62258	YWHAE	14-3-3 protein epsilon	18,73
Q9BUJ2	HNRNPUL1	Heterogeneous nuclear ribonucleoprotein U-like protein 1	18,70
Q9H1I8	ASCC2	Activating signal cointegrator 1 complex subunit 2	18,65
P63010	AP2B1	AP-2 complex subunit beta	18,39
Q92522	H1-10	Histone H1.10	18,14
E9PQ57	RAE1	Ribonucleic acid export 1	17,81
Q08211	DHX9	ATP-dependent RNA helicase A	17,80
Q3KQU3	MAP7D1	MAP7 domain-containing protein 1	17,69
P46940	IQGAP1	Ras GTPase-activating-like protein IQGAP1	17,43
Q86YT6	MIB1	E3 ubiquitin-protein ligase MIB1	17,41
Q9UHB6	LIMA1	LIM domain and actin-binding protein 1	17,40
P23396	RPS3	Small ribosomal subunit protein uS3	17,37
Q6ZMY3	SPOCD1	SPOC domain-containing protein 1	17,29
Q5LJA9	UCHL5	Ubiquitin carboxyl-terminal hydrolase (Fragment)	17,26
Q12797	ASPH	Aspartyl/asparaginyl beta-hydroxylase	17,18
O15042	U2SURP	U2 snRNP-associated SURP motif-containing protein	16,94
Q96I25	RBM17	Splicing factor 45	16,93
Q9BRJ6	C7orf50	Uncharacterized protein C7orf50	16,89
Q9GZR7	DDX24	ATP-dependent RNA helicase DDX24	16,84
P48634	PRRC2A	Protein PRRC2A	16,64
H0Y2W2	ATAD3A	ATPase family AAA domain containing 3A (Fragment)	16,43
A0A7I2YQG2	RPL19	Ribosomal protein L19	16,42
P27348	YWHAQ	14-3-3 protein theta	16,41
Q08174	PCDH1	Protocadherin-1	16,40
P30050	RPL12	Large ribosomal subunit protein uL11	16,16
O00541	PES1	Pescadillo homolog	16,13
O43795	MYO1B	Unconventional myosin-Ib	15,83
A0A8I5KWT8	MYH9	Myosin heavy chain 9	15,62
Q16666	IFI16	Gamma-interferon-inducible protein 16	15,46
P46778	RPL21	Large ribosomal subunit protein eL21	15,45
A0A0G2JLB3	GBA1	Glucosylceramidase	15,45
P13674	P4HA1	Prolyl 4-hydroxylase subunit alpha-1	15,42
Q5STZ8	ABCF1	ATP binding cassette subfamily F member 1	15,39
P62266	RPS23	Small ribosomal subunit protein uS12	15,36
P51398	DAP3	Small ribosomal subunit protein mS29	15,12
Q8IXQ6	PARP9	Protein mono-ADP-ribosyltransferase PARP9	15,09
A0A087X009	ABO	ABO, alpha 1-3-N-acetylgalactosaminyltransferase and alpha 1-3-galactosyltransferase	14,98
Q9UGJ0	PRKAG2	5'-AMP-activated protein kinase subunit gamma-2	14,94
Q9P2E9	RRBP1	Ribosome-binding protein 1	14,87
Q9P206	KIAA1522	Uncharacterized protein KIAA1522	14,79
Q14258	TRIM25	E3 ubiquitin/ISG15 ligase TRIM25	14,74
Q96AG4	LRRC59	Leucine-rich repeat-containing protein 59	14,73
O43865	AHCYL1	S-adenosylhomocysteine hydrolase-like protein 1	14,66
Q86V48	LUZP1	Leucine zipper protein 1	14,63
Q92499	DDX1	ATP-dependent RNA helicase DDX1	14,50
G0XQ39	STIM1	STIM1L	14,46
Q8N128	FAM177A1	Protein FAM177A1	14,14
Q99985	SEMA3C	Semaphorin-3C	14,02
O00425	IGF2BP3	Insulin-like growth factor 2 mRNA-binding protein 3	14,01
Q9NUQ6	SPATS2L	SPATS2-like protein	13,99
A0A0A6YYL6	RPL17-C18orf32	60S ribosomal protein L17	13,82
A0A7I2V535	NONO	Non-POU domain containing octamer binding	13,70
A0A8I5KX72	NKRF	NFKB repressing factor	13,65
O00411	POLRMT	DNA-directed RNA polymerase, mitochondrial	13,34
H0YKD8	RPL28	60S ribosomal protein L28	13,20
Q6UWP7	LCLAT1	Lysocardiolipin acyltransferase 1	13,18
Q7Z2W4	ZC3HAV1	Zinc finger CCCH-type antiviral protein 1	13,12
Q13263	TRIM28	Transcription intermediary factor 1-beta	13,02
Q9NZM1	MYOF	Myoferlin	12,97
Q07065	CKAP4	Cytoskeleton-associated protein 4	12,79
Q9UMS4	PRPF19	Pre-mRNA-processing factor 19	12,77
Q8NFW8	CMAS	N-acylneuraminate cytidylyltransferase	12,74
Q9Y394	DHRS7	Dehydrogenase/reductase SDR family member 7	12,66
Q9Y520	PRRC2C	Protein PRRC2C	12,64
P80723	BASP1	Brain acid soluble protein 1	12,63
A0A5F9ZHP1	ASCC1	Activating signal cointegrator 1 complex subunit 1	12,59
Q9H089	LSG1	Large subunit GTPase 1 homolog	12,58
Q15020	SART3	Squamous cell carcinoma antigen recognized by T-cells 3	12,58
Q92900	UPF1	Regulator of nonsense transcripts 1	12,57
A0FGR8	ESYT2	Extended synaptotagmin-2	12,51
A0A286YFF7	PPT1	Palmitoyl-protein thioesterase 1	12,48
P40121	CAPG	Macrophage-capping protein	12,43
P32322	PYCR1	Pyrroline-5-carboxylate reductase 1, mitochondrial	12,38
Q9Y3U8	RPL36	Large ribosomal subunit protein eL36	12,37
Q92665	MRPS31	Small ribosomal subunit protein mS31	12,34
O00401	WASL	Actin nucleation-promoting factor WASL	12,31
Q15629	TRAM1	Translocating chain-associated membrane protein 1	12,17
O75909	CCNK	Cyclin-K	12,13
Q16698	DECR1	2,4-dienoyl-CoA reductase [(3E)-enoyl-CoA-producing], mitochondrial	12,00
Q9Y3I0	RTCB	RNA-splicing ligase RtcB homolog	11,97
H7BY16	NCL	Nucleolin	11,96
O14908	GIPC1	PDZ domain-containing protein GIPC1	11,95
A0A0J9YXF2	PON2	Paraoxonase	11,93
A1L170	C1orf226	Uncharacterized protein C1orf226	11,90
Q04917	YWHAH	14-3-3 protein eta	11,84
Q6ZMP0	THSD4	Thrombospondin type-1 domain-containing protein 4	11,83
P32969	RPL9	Large ribosomal subunit protein uL6	11,76
Q14444	CAPRIN1	Caprin-1	11,70
P23284	PPIB	Peptidyl-prolyl cis-trans isomerase B	11,62
P11940	PABPC1	Polyadenylate-binding protein 1	11,52
E7ERS3	ZC3H18	Zinc finger CCCH-type containing 18	11,50
Q9H6F5	CCDC86	Coiled-coil domain-containing protein 86	11,47
Q9P2J5	LARS1	Leucine--tRNA ligase, cytoplasmic	11,45
P51665	PSMD7	26S proteasome non-ATPase regulatory subunit 7	11,44
Q6ICG6	KIAA0930	Uncharacterized protein KIAA0930	11,39
Q9NZB2	FAM120A	Constitutive coactivator of PPAR-gamma-like protein 1	11,27
P49368	CCT3	T-complex protein 1 subunit gamma	11,25
A0A994J6B4	FIP1L1	Pre-mRNA 3'-end-processing factor FIP1	11,25
P38919	EIF4A3	Eukaryotic initiation factor 4A-III	11,17
Q99459	CDC5L	Cell division cycle 5-like protein	11,13
O94832	MYO1D	Unconventional myosin-Id	11,12
Q15650	TRIP4	Activating signal cointegrator 1	11,11
Q9BT40	INPP5K	Inositol polyphosphate 5-phosphatase K	11,07
Q9UN81	L1RE1	LINE-1 retrotransposable element ORF1 protein	11,07
P41091	EIF2S3	Eukaryotic translation initiation factor 2 subunit 3	11,07
E9PFW3	AP2M1	AP-2 complex subunit mu	11,03
O95782	AP2A1	AP-2 complex subunit alpha-1	11,01
P02452	COL1A1	Collagen alpha-1(I) chain	10,97
P06396	GSN	Gelsolin	10,95
Q9BZE4	GTPBP4	GTP-binding protein 4	10,91
Q9NW13	RBM28	RNA-binding protein 28	10,89
P46776	RPL27A	Large ribosomal subunit protein uL15	10,74
E7EVA0	MAP4	Microtubule-associated protein	10,73
O43293	DAPK3	Death-associated protein kinase 3	10,70
P05362	ICAM1	Intercellular adhesion molecule 1	10,70
Q9Y4P3	TBL2	Transducin beta-like protein 2	10,67
O95425	SVIL	Supervillin	10,65
O00622	CCN1	CCN family member 1	10,62
Q8TC12	RDH11	Retinol dehydrogenase 11	10,58
Q8WVM8	SCFD1	Sec1 family domain-containing protein 1	10,53
Q14192	FHL2	Four and a half LIM domains protein 2	10,51
P83111	LACTB	Serine beta-lactamase-like protein LACTB, mitochondrial	10,50
A0A1Y0BRN3	MYO6	Unconventional myosin-VI	10,50
Q53EP0	FNDC3B	Fibronectin type III domain-containing protein 3B	10,50
Q13049	TRIM32	E3 ubiquitin-protein ligase TRIM32	10,49
A0A499FHX9	MCM3	DNA replication licensing factor MCM3	10,48
J3KTA4	DDX5	Probable ATP-dependent RNA helicase DDX5	10,46
Q96SQ9	CYP2S1	Cytochrome P450 2S1	10,34
A0A0A0MTJ9	NCEH1	Neutral cholesterol ester hydrolase 1	10,32
P35268	RPL22	Large ribosomal subunit protein eL22	10,31
Q658Y4	FAM91A1	Protein FAM91A1	10,25
A0A8Q3WKF1	MRPS27	Mitochondrial ribosomal protein S27	10,24
P50281	MMP14	Matrix metalloproteinase-14	10,23
Q13136	PPFIA1	Liprin-alpha-1	10,22
Q70CQ2	USP34	Ubiquitin carboxyl-terminal hydrolase 34	10,19
Q8N556	AFAP1	Actin filament-associated protein 1	10,19
Q9GZL7	WDR12	Ribosome biogenesis protein WDR12	10,17
X6R8F3	LCN2	Lipocalin 2	10,15
P83731	RPL24	Large ribosomal subunit protein eL24	10,13
A0A6Q8PG37	KIF2A	Kinesin-like protein	10,11
O75616	ERAL1	GTPase Era, mitochondrial	10,08
P31431	SDC4	Syndecan-4	10,07
Q9Y6H1	CHCHD2	Coiled-coil-helix-coiled-coil-helix domain-containing protein 2	10,06
P49748	ACADVL	Very long-chain specific acyl-CoA dehydrogenase, mitochondrial	9,97
O95470	SGPL1	Sphingosine-1-phosphate lyase 1	9,96
O15031	PLXNB2	Plexin-B2	9,93
Q12906	ILF3	Interleukin enhancer-binding factor 3	9,91
A0A8J9BZL8	GOLGA2	Golgin A2	9,89
Q13838	DDX39B	Spliceosome RNA helicase DDX39B	9,86
Q9Y6N5	SQOR	Sulfide:quinone oxidoreductase, mitochondrial	9,81
Q8WVM0	TFB1M	Dimethyladenosine transferase 1, mitochondrial	9,81
A0A7P0TAU8	OAS3	2'-5' oligoadenylate synthase	9,80
P67809	YBX1	Y-box-binding protein 1	9,78
A0A6Q8PFE1	AIFM1	Apoptosis inducing factor mitochondria associated 1	9,74
Q7Z2T5	TRMT1L	TRMT1-like protein	9,74
Q8WU90	ZC3H15	Zinc finger CCCH domain-containing protein 15	9,66
P47897	QARS1	Glutamine--tRNA ligase	9,62
P62829	RPL23	Large ribosomal subunit protein uL14	9,56
Q16706	MAN2A1	Alpha-mannosidase 2	9,54
Q9P258	RCC2	Protein RCC2	9,43
P46781	RPS9	Small ribosomal subunit protein uS4	9,42
H0YFD6	HADHA	enoyl-CoA hydratase	9,40
Q9HAU0	PLEKHA5	Pleckstrin homology domain-containing family A member 5	9,39
Q6DD88	ATL3	Atlastin-3	9,39
F8W727	RPL32	Ribosomal protein L32	9,37
Q9Y678	COPG1	Coatomer subunit gamma-1	9,37
A0A804HKI2	LRPPRC	Leucine rich pentatricopeptide repeat containing	9,33
A0A669KB89	SCRIB	Scribble planar cell polarity protein	9,32
Q14451	GRB7	Growth factor receptor-bound protein 7	9,27
Q8TDB6	DTX3L	E3 ubiquitin-protein ligase DTX3L	9,24
A0A5F9ZI21	ARHGEF2	Rho guanine nucleotide exchange factor 2	9,23
P63104	YWHAZ	14-3-3 protein zeta/delta	9,13
Q9H0D6	XRN2	5'-3' exoribonuclease 2	9,12
Q5T0Z8	C6orf132	Uncharacterized protein C6orf132	9,07
P54819	AK2	Adenylate kinase 2, mitochondrial	9,06
P29728	OAS2	2'-5'-oligoadenylate synthase 2	9,05
Q92599	SEPTIN8	Septin-8	9,01
Q9BSJ8	ESYT1	Extended synaptotagmin-1	8,95
Q12849	GRSF1	G-rich sequence factor 1	8,95
J3KPT4	TRABD	TraB domain containing	8,95
Q9NZ08	ERAP1	Endoplasmic reticulum aminopeptidase 1	8,86
Q6NZI2	CAVIN1	Caveolae-associated protein 1	8,82
A0A3B3IT54	C8orf33	Chromosome 8 open reading frame 33	8,82
E7ETE2	HARS1	Histidine--tRNA ligase, cytoplasmic	8,79
Q8IY21	DDX60	Probable ATP-dependent RNA helicase DDX60	8,78
Q9BRZ2	TRIM56	E3 ubiquitin-protein ligase TRIM56	8,73
Q06787	FMR1	Fragile X messenger ribonucleoprotein 1	8,71
O60506	SYNCRIP	Heterogeneous nuclear ribonucleoprotein Q	8,70
A0A5F9ZHN9	ALDH3A2	Aldehyde dehydrogenase	8,68
A0A024QZP7	CDK1	Cell division cycle 2, G1 to S and G2 to M, isoform CRA_a	8,68
P54136	RARS1	Arginine--tRNA ligase, cytoplasmic	8,67
Q14554	PDIA5	Protein disulfide-isomerase A5	8,66
Q15642	TRIP10	Cdc42-interacting protein 4	8,65
A0A8Q3SIU1	RASAL2	RAS protein activator like 2	8,63
P11498	PC	Pyruvate carboxylase, mitochondrial	8,61
O76031	CLPX	ATP-dependent Clp protease ATP-binding subunit clpX-like, mitochondrial	8,60
Q9ULT8	HECTD1	E3 ubiquitin-protein ligase HECTD1	8,58
P20930	FLG	Filaggrin	8,55
P31946	YWHAB	14-3-3 protein beta/alpha	8,51
Q9H0S4	DDX47	Probable ATP-dependent RNA helicase DDX47	8,50
P84022	SMAD3	Mothers against decapentaplegic homolog 3	8,43
Q9NTJ3	SMC4	Structural maintenance of chromosomes protein 4	8,42
A0A2R8YDH4	1 SV	Tight junction protein ZO-2	8,41
A0A590UI43	CSNK1A1	Casein kinase 1 alpha 1	8,40
Q8N3C0	ASCC3	Activating signal cointegrator 1 complex subunit 3	8,33
Q14739	LBR	Delta(14)-sterol reductase LBR	8,28
Q15738	NSDHL	Sterol-4-alpha-carboxylate 3-dehydrogenase, decarboxylating	8,27
P53992	SEC24C	Protein transport protein Sec24C	8,23
Q8IZH2	XRN1	5'-3' exoribonuclease 1	8,22
P46087	NOP2	Probable 28S rRNA (cytosine(4447)-C(5))-methyltransferase	8,16
Q13011	ECH1	Delta(3,5)-Delta(2,4)-dienoyl-CoA isomerase, mitochondrial	8,16
P50454	SERPINH1	Serpin H1	8,15
Q96AQ6	PBXIP1	Pre-B-cell leukemia transcription factor-interacting protein 1	8,15
P29590	PML	Protein PML	8,13
Q9Y399	MRPS2	Small ribosomal subunit protein uS2m	8,07
O43143	DHX15	ATP-dependent RNA helicase DHX15	8,04
Q15019	SEPTIN2	Septin-2	8,03
P82933	MRPS9	Small ribosomal subunit protein uS9m	8,03
P16989	YBX3	Y-box-binding protein 3	8,03
Q86XZ4	SPATS2	Spermatogenesis-associated serine-rich protein 2	8,01
Q9Y6E2	BZW2	eIF5-mimic protein 1	7,97
Q16531	DDB1	DNA damage-binding protein 1	7,95
Q9NVV4	MTPAP	Poly(A) RNA polymerase, mitochondrial	7,94
P07476	IVL	Involucrin	7,94
P05783	KRT18	Keratin, type I cytoskeletal 18	7,91
O43707	ACTN4	Alpha-actinin-4	7,90
P04040	CAT	Catalase	7,87
Q92598	HSPH1	Heat shock protein 105 kDa	7,81
P14868	DARS1	Aspartate--tRNA ligase, cytoplasmic	7,77
A0A3B3ITT5	RPL29	60S ribosomal protein L29	7,75
A0A994J4D9	4 SV	Exosome component 4	7,74
Q15008	PSMD6	26S proteasome non-ATPase regulatory subunit 6	7,74
Q02952	AKAP12	A-kinase anchor protein 12	7,72
Q14004	CDK13	Cyclin-dependent kinase 13	7,72
P61158	ACTR3	Actin-related protein 3	7,72
Q9BWF3	RBM4	RNA-binding protein 4	7,70
A0A087WT44	HMOX2	heme oxygenase (biliverdin-producing)	7,70
Q9NVI7	ATAD3A	ATPase family AAA domain-containing protein 3A	7,69
P78527	PRKDC	DNA-dependent protein kinase catalytic subunit	7,66
F8VWW7	SPRYD3	SPRY domain containing 3	7,60
Q13435	SF3B2	Splicing factor 3B subunit 2	7,59
Q99755	PIP5K1A	Phosphatidylinositol 4-phosphate 5-kinase type-1 alpha	7,53
O43278	SPINT1	Kunitz-type protease inhibitor 1	7,53
A0A494C0G7	DIMT1	rRNA adenine N(6)-methyltransferase	7,52
P35606	COPB2	Coatomer subunit beta'	7,51
Q03169	TNFAIP2	Tumor necrosis factor alpha-induced protein 2	7,50
Q70UQ0	IKBIP	Inhibitor of nuclear factor kappa-B kinase-interacting protein	7,49
C9JMN9	PDIA4	Protein disulfide-isomerase	7,49
P55084	HADHB	Trifunctional enzyme subunit beta, mitochondrial	7,48
E7EX17	EIF4B	Eukaryotic translation initiation factor 4B	7,48
F8WE88	MYO5A	Unconventional myosin-Va	7,46
O00159	MYO1C	Unconventional myosin-Ic	7,46
Q8NHP8	PLBD2	Putative phospholipase B-like 2	7,43
P62280	RPS11	Small ribosomal subunit protein uS17	7,39
P08236	GUSB	Beta-glucuronidase	7,37
A0A994J7M6	PIGS	Phosphatidylinositol glycan anchor biosynthesis class S	7,35
P43358	MAGEA4	Melanoma-associated antigen 4	7,25
Q15582	TGFBI	Transforming growth factor-beta-induced protein ig-h3	7,21
P09874	PARP1	Poly [ADP-ribose] polymerase 1	7,19
Q15046	KARS1	Lysine--tRNA ligase	7,12
P19525	EIF2AK2	Interferon-induced, double-stranded RNA-activated protein kinase	7,12
P34897	SHMT2	Serine hydroxymethyltransferase, mitochondrial	7,10
P41250	GARS1	Glycine--tRNA ligase	7,08
Q86UE4	MTDH	Protein LYRIC	7,03
P08579	SNRPB2	U2 small nuclear ribonucleoprotein B''	7,01
O60568	PLOD3	Multifunctional procollagen lysine hydroxylase and glycosyltransferase LH3	7,01
O75676	RPS6KA4	Ribosomal protein S6 kinase alpha-4	7,00
A0A8I5KNN2	DDX21	RNA helicase	6,99
P12956	XRCC6	X-ray repair cross-complementing protein 6	6,97
P07686	HEXB	Beta-hexosaminidase subunit beta	6,96
Q00577	PURA	Transcriptional activator protein Pur-alpha	6,94
P68400	CSNK2A1	Casein kinase II subunit alpha	6,94
O15460	P4HA2	Prolyl 4-hydroxylase subunit alpha-2	6,91
A0A087WUZ3	SPTBN1	Spectrin beta chain	6,91
O75844	ZMPSTE24	CAAX prenyl protease 1 homolog	6,91
P52597	HNRNPF	Heterogeneous nuclear ribonucleoprotein F	6,90
P09758	TACSTD2	Tumor-associated calcium signal transducer 2	6,88
O75390	CS	Citrate synthase, mitochondrial	6,86
P08240	SRPRA	Signal recognition particle receptor subunit alpha	6,86
P30040	ERP29	Endoplasmic reticulum resident protein 29	6,85
O75165	DNAJC13	DnaJ homolog subfamily C member 13	6,85
P07814	EPRS1	Bifunctional glutamate/proline--tRNA ligase	6,84
O15355	PPM1G	Protein phosphatase 1G	6,83
P05787	KRT8	Keratin, type II cytoskeletal 8	6,81
Q9UMD9	COL17A1	Collagen alpha-1(XVII) chain	6,77
D6RBZ0	HNRNPAB	Heterogeneous nuclear ribonucleoprotein A/B	6,76
P35221	CTNNA1	Catenin alpha-1	6,75
O60716	CTNND1	Catenin delta-1	6,74
A0A804HHU1	MAN1B1	alpha-1,2-Mannosidase	6,72
Q7L2E3	DHX30	ATP-dependent RNA helicase DHX30	6,71
H3BLV9	SRPK1	SRSF protein kinase 1 (Fragment)	6,68
Q13151	HNRNPA0	Heterogeneous nuclear ribonucleoprotein A0	6,68
Q9NQC3	RTN4	Reticulon-4	6,66
A0A8Q3WMA7	SFPQ	Splicing factor proline and glutamine rich	6,64
Q9UKS6	PACSIN3	Protein kinase C and casein kinase substrate in neurons protein 3	6,63
Q8IWA4	MFN1	Mitofusin-1	6,59
Q96CS3	FAF2	FAS-associated factor 2	6,58
A0A087WZN1	IDH3B	Isocitrate dehydrogenase [NAD] subunit, mitochondrial	6,50
P17987	TCP1	T-complex protein 1 subunit alpha	6,49
Q96GM8	TOE1	Target of EGR1 protein 1	6,45
A0A994J6W3	SPTAN1	Spectrin alpha, non-erythrocytic 1	6,44
Q9H9L3	ISG20L2	Interferon-stimulated 20 kDa exonuclease-like 2	6,41
O43847	NRDC	Nardilysin	6,41
P18564	ITGB6	Integrin beta-6	6,41
P26196	DDX6	Probable ATP-dependent RNA helicase DDX6	6,40
Q9Y3F4	STRAP	Serine-threonine kinase receptor-associated protein	6,37
Q13155	AIMP2	Aminoacyl tRNA synthase complex-interacting multifunctional protein 2	6,36
Q9Y606	PUS1	Pseudouridylate synthase 1 homolog	6,35
Q86W92	PPFIBP1	Liprin-beta-1	6,35
O43290	SART1	U4/U6.U5 tri-snRNP-associated protein 1	6,33
P00505	GOT2	Aspartate aminotransferase, mitochondrial	6,33
Q96JK2	DCAF5	DDB1- and CUL4-associated factor 5	6,33
D3DQV9	EIF4G2	Eukaryotic translation initiation factor 4 gamma 2 (Fragment)	6,31
P62888	RPL30	Large ribosomal subunit protein eL30	6,31
Q9P035	HACD3	Very-long-chain (3R)-3-hydroxyacyl-CoA dehydratase 3	6,30
Q92890	UFD1	Ubiquitin recognition factor in ER-associated degradation protein 1	6,28
P17931	LGALS3	Galectin-3	6,27
Q99613	EIF3C	Eukaryotic translation initiation factor 3 subunit C	6,27
Q7L0Y3	TRMT10C	tRNA methyltransferase 10 homolog C	6,27
Q969G5	CAVIN3	Caveolae-associated protein 3	6,27
O95159	ZFPL1	Zinc finger protein-like 1	6,26
O15162	PLSCR1	Phospholipid scramblase 1	6,24
Q6YN16	HSDL2	Hydroxysteroid dehydrogenase-like protein 2	6,24
Q15437	SEC23B	Protein transport protein Sec23B	6,24
Q9Y5B9	SUPT16H	FACT complex subunit SPT16	6,18
Q08AF3	SLFN5	Schlafen family member 5	6,18
Q9BVP2	GNL3	Guanine nucleotide-binding protein-like 3	6,18
Q5T0N5	FNBP1L	Formin-binding protein 1-like	6,18
A0A0A0MRX1	ELAVL2	ELAV-like protein	6,17
E5KLJ9	OPA1	Dynamin-like 120 kDa protein, mitochondrial	6,15
Q15006	EMC2	ER membrane protein complex subunit 2	6,14
P04181	OAT	Ornithine aminotransferase, mitochondrial	6,07
J3KPS0	DNAJB12	DnaJ (Hsp40) homolog, subfamily B, member 12, isoform CRA_c	6,04
Q99575	POP1	Ribonucleases P/MRP protein subunit POP1	6,03
P47755	CAPZA2	F-actin-capping protein subunit alpha-2	6,02
Q9NXH8	TOR4A	Torsin-4A	6,01
Q09161	NCBP1	Nuclear cap-binding protein subunit 1	6,00
Q14008	CKAP5	Cytoskeleton-associated protein 5	5,97
Q9UQ35	SRRM2	Serine/arginine repetitive matrix protein 2	5,95
P40926	MDH2	Malate dehydrogenase, mitochondrial	5,94
Q08J23	NSUN2	RNA cytosine C(5)-methyltransferase NSUN2	5,92
A0A8J9AUJ2	RAB3GAP1	Rab3 GTPase-activating protein catalytic subunit	5,92
P30740	SERPINB1	Leukocyte elastase inhibitor	5,91
A0A994J7P9	FXR2	FMR1 autosomal homolog 2	5,91
P23526	AHCY	Adenosylhomocysteinase	5,90
A0A669KBH4	CUL7	Cullin 7	5,90
Q9H5Q4	TFB2M	Dimethyladenosine transferase 2, mitochondrial	5,90
P50990	CCT8	T-complex protein 1 subunit theta	5,89
Q9BYK8	HELZ2	Helicase with zinc finger domain 2	5,86
M0QYS1	RPL13A	60S ribosomal protein L13a (Fragment)	5,84
Q9Y4F5	CEP170B	Centrosomal protein of 170 kDa protein B	5,81
O14744	PRMT5	Protein arginine N-methyltransferase 5	5,80
O75534	CSDE1	Cold shock domain-containing protein E1	5,80
Q9H0V9	LMAN2L	VIP36-like protein	5,79
Q9NUL3	STAU2	Double-stranded RNA-binding protein Staufen homolog 2	5,79
A0A0G2JN29	NOMO1	Nodal modulator 1	5,78
Q96HE7	ERO1A	ERO1-like protein alpha	5,76
O94925	GLS	Glutaminase kidney isoform, mitochondrial	5,74
Q8N684	CPSF7	Cleavage and polyadenylation specificity factor subunit 7	5,70
P50991	CCT4	T-complex protein 1 subunit delta	5,68
Q6P1M0	SLC27A4	Long-chain fatty acid transport protein 4	5,65
Q9HCE1	MOV10	Helicase MOV-10	5,65
P23219	PTGS1	Prostaglandin G/H synthase 1	5,64
P40938	RFC3	Replication factor C subunit 3	5,63
Q06265	EXOSC9	Exosome complex component RRP45	5,62
Q96N66	MBOAT7	Lysophospholipid acyltransferase 7	5,62
P11279	LAMP1	Lysosome-associated membrane glycoprotein 1	5,62
Q9BZF1	OSBPL8	Oxysterol-binding protein-related protein 8	5,60
Q8IWT6	LRRC8A	Volume-regulated anion channel subunit LRRC8A	5,59
Q9UHR4	BAIAP2L1	Brain-specific angiogenesis inhibitor 1-associated protein 2-like protein 1	5,58
Q7KZF4	SND1	Staphylococcal nuclease domain-containing protein 1	5,56
Q6UY14	ADAMTSL4	ADAMTS-like protein 4	5,56
P21333	FLNA	Filamin-A	5,55
Q5JPT2	SH3KBP1	SH3 domain containing kinase binding protein 1	5,53
Q9NQT4	EXOSC5	Exosome complex component RRP46	5,53
Q14240	EIF4A2	Eukaryotic initiation factor 4A-II	5,53
P05120	SERPINB2	Plasminogen activator inhibitor 2	5,52
Q9UHD8	SEPTIN9	Septin-9	5,51
Q9UJS0	SLC25A13	Electrogenic aspartate/glutamate antiporter SLC25A13, mitochondrial	5,49
M0QXB4	COPE	Coatomer subunit epsilon	5,46
Q6WKZ4	RAB11FIP1	Rab11 family-interacting protein 1	5,45
Q12907	LMAN2	Vesicular integral-membrane protein VIP36	5,45
Q07960	ARHGAP1	Rho GTPase-activating protein 1	5,43
Q9UHB9	SRP68	Signal recognition particle subunit SRP68	5,41
P08779	KRT16	Keratin, type I cytoskeletal 16	5,40
Q9UBB4	ATXN10	Ataxin-10	5,39
Q9H223	EHD4	EH domain-containing protein 4	5,38
Q969V3	NCLN	BOS complex subunit NCLN	5,37
O15144	ARPC2	Actin-related protein 2/3 complex subunit 2	5,36
O95202	LETM1	Mitochondrial proton/calcium exchanger protein	5,35
Q14764	MVP	Major vault protein	5,34
Q9BUF5	TUBB6	Tubulin beta-6 chain	5,33
H7C2Q8	EBNA1BP2	EBNA1 binding protein 2	5,33
P27635	RPL10	Large ribosomal subunit protein uL16	5,30
P42766	RPL35	Large ribosomal subunit protein uL29	5,29
K7EJJ5	CLTC	Clathrin heavy chain	5,28
P16615	ATP2A2	Sarcoplasmic/endoplasmic reticulum calcium ATPase 2	5,28
C9JEJ2	PCYT1A	choline-phosphate cytidylyltransferase	5,26
A0A7I2V5M7	G3BP1	G3BP stress granule assembly factor 1	5,25
P40937	RFC5	Replication factor C subunit 5	5,24
Q8WUA4	GTF3C2	General transcription factor 3C polypeptide 2	5,24
Q9UK99	FBXO3	F-box only protein 3	5,24
Q86X29	LSR	Lipolysis-stimulated lipoprotein receptor	5,23
P60842	EIF4A1	Eukaryotic initiation factor 4A-I	5,22
Q9UGP8	SEC63	Translocation protein SEC63 homolog	5,21
Q9Y639	NPTN	Neuroplastin	5,21
Q14574	DSC3	Desmocollin-3	5,19
A0A3B3ISF9	ECE1	Endothelin converting enzyme 1	5,18
P05198	EIF2S1	Eukaryotic translation initiation factor 2 subunit 1	5,17
P62244	RPS15A	Small ribosomal subunit protein uS8	5,15
P50416	CPT1A	Carnitine O-palmitoyltransferase 1, liver isoform	5,14
E7ERK9	EIF2B4	Eukaryotic translation initiation factor 2B subunit delta	5,13
Q13753	LAMC2	Laminin subunit gamma-2	5,12
Q8NHQ9	DDX55	ATP-dependent RNA helicase DDX55	5,12
P07204	THBD	Thrombomodulin	5,11
Q7L5N7	LPCAT2	Lysophosphatidylcholine acyltransferase 2	5,11
Q14435	GALNT3	Polypeptide N-acetylgalactosaminyltransferase 3	5,10
O75369	FLNB	Filamin-B	5,10
Q10471	GALNT2	Polypeptide N-acetylgalactosaminyltransferase 2	5,10
Q3ZCQ8	TIMM50	Mitochondrial import inner membrane translocase subunit TIM50	5,09
Q9P0V3	SH3BP4	SH3 domain-binding protein 4	5,09
Q8N884	CGAS	Cyclic GMP-AMP synthase	5,09
Q6ZXV5	TMTC3	Protein O-mannosyl-transferase TMTC3	5,07
Q6PKG0	LARP1	La-related protein 1	5,07
O94973	AP2A2	AP-2 complex subunit alpha-2	5,06
Q9BW92	TARS2	Threonine--tRNA ligase, mitochondrial	5,05
P07305	H1-0	Histone H1.0	5,05
O43681	GET3	ATPase GET3	5,03
A0A7I2V3S3	EIF3E	Eukaryotic translation initiation factor 3 subunit E	5,03
Q14697	GANAB	Neutral alpha-glucosidase AB	5,00
Q9UKD2	MRTO4	mRNA turnover protein 4 homolog	4,98
A0A8V8TM76	TAP1	ABC-type antigen peptide transporter	4,98
A0A8I5KW96	AK4P3	Adenylate kinase 4, mitochondrial	4,96
Q13643	FHL3	Four and a half LIM domains protein 3	4,94
P13010	XRCC5	X-ray repair cross-complementing protein 5	4,91
A0A2R8Y5A3	CTNNB1	Catenin beta 1	4,90
Q86UY5	FAM83A	Protein FAM83A	4,87
P61964	WDR5	WD repeat-containing protein 5	4,86
Q9UJ70	NAGK	N-acetyl-D-glucosamine kinase	4,85
Q13501	SQSTM1	Sequestosome-1	4,84
Q00653	NFKB2	Nuclear factor NF-kappa-B p100 subunit	4,83
P04843	RPN1	Dolichyl-diphosphooligosaccharide--protein glycosyltransferase subunit 1	4,83
A0A0A0MTC1	RNF213	Ring finger protein 213	4,83
Q8TF74	WIPF2	WAS/WASL-interacting protein family member 2	4,83
Q13510	ASAH1	Acid ceramidase	4,81
P35998	PSMC2	26S proteasome regulatory subunit 7	4,81
Q9UHG3	PCYOX1	Prenylcysteine oxidase 1	4,80
A0A087WZ40	MAP7	Microtubule associated protein 7	4,80
Q13610	PWP1	Periodic tryptophan protein 1 homolog	4,79
Q9H7Z7	PTGES2	Prostaglandin E synthase 2	4,79
Q13191	CBLB	E3 ubiquitin-protein ligase CBL-B	4,78
B3KS98	EIF3H	Eukaryotic translation initiation factor 3 subunit H	4,78
A0A804HII9	ACTN1	Actinin alpha 1	4,77
P40227	CCT6A	T-complex protein 1 subunit zeta	4,71
Q9NS86	LANCL2	LanC-like protein 2	4,70
P19971	TYMP	Thymidine phosphorylase	4,68
A0A8J8YWN6	CYB5R3	cytochrome-b5 reductase	4,68
P07199	CENPB	Major centromere autoantigen B	4,67
P61011	SRP54	Signal recognition particle subunit SRP54	4,66
P82673	MRPS35	Small ribosomal subunit protein mS35	4,66
Q15646	OASL	2'-5'-oligoadenylate synthase-like protein	4,65
Q02809	PLOD1	Procollagen-lysine,2-oxoglutarate 5-dioxygenase 1	4,64
P10398	ARAF	Serine/threonine-protein kinase A-Raf	4,64
Q9UBN7	HDAC6	Histone deacetylase 6	4,63
Q7L3T8	PARS2	Probable proline--tRNA ligase, mitochondrial	4,62
Q562R1	ACTBL2	Beta-actin-like protein 2	4,62
P55265	ADAR	Double-stranded RNA-specific adenosine deaminase	4,62
J3QK89	CHERP	Calcium homeostasis endoplasmic reticulum protein	4,62
P26368	U2AF2	Splicing factor U2AF 65 kDa subunit	4,62
P62195	PSMC5	26S proteasome regulatory subunit 8	4,59
Q5JTV8	TOR1AIP1	Torsin-1A-interacting protein 1	4,59
P49411	TUFM	Elongation factor Tu, mitochondrial	4,58
P27708	CAD	CAD protein	4,55
P35249	RFC4	Replication factor C subunit 4	4,55
P09651	HNRNPA1	Heterogeneous nuclear ribonucleoprotein A1	4,54
Q9H9J2	MRPL44	Large ribosomal subunit protein mL44	4,52
Q01780	EXOSC10	Exosome component 10	4,51
Q99832	CCT7	T-complex protein 1 subunit eta	4,50
I3L4N8	ACTG1	Actin gamma 1	4,47
Q5T1Z8	PUM1	Pumilio RNA binding family member 1	4,47
A0A669KBL1	USP7	Ubiquitin carboxyl-terminal hydrolase 7	4,46
P22314	UBA1	Ubiquitin-like modifier-activating enzyme 1	4,45
Q5SRN5	HLA-A	Major histocompatibility complex, class I, A	4,44
A0A087WX97	BCL2L13	BCL2 like 13	4,44
P82650	MRPS22	Small ribosomal subunit protein mS22	4,44
Q00059	TFAM	Transcription factor A, mitochondrial	4,44
Q15075	EEA1	Early endosome antigen 1	4,44
Q9C0C2	TNKS1BP1	182 kDa tankyrase-1-binding protein	4,43
Q9Y4E8	USP15	Ubiquitin carboxyl-terminal hydrolase 15	4,42
J3QLS3	MRPS7	Mitochondrial ribosomal protein S7	4,41
P23634	ATP2B4	Plasma membrane calcium-transporting ATPase 4	4,40
Q92643	PIGK	GPI-anchor transamidase	4,40
P11021	HSPA5	Endoplasmic reticulum chaperone BiP	4,40
P22307	SCP2	Sterol carrier protein 2	4,39
P27105	STOM	Stomatin	4,38
Q53H12	AGK	Acylglycerol kinase, mitochondrial	4,37
P29317	EPHA2	Ephrin type-A receptor 2	4,36
Q9UG56	PISD	Phosphatidylserine decarboxylase proenzyme, mitochondrial	4,33
Q13751	LAMB3	Laminin subunit beta-3	4,33
Q96RP9	GFM1	Elongation factor G, mitochondrial	4,32
Q9NWT1	PAK1IP1	p21-activated protein kinase-interacting protein 1	4,32
Q12904	AIMP1	Aminoacyl tRNA synthase complex-interacting multifunctional protein 1	4,32
P50750	CDK9	Cyclin-dependent kinase 9	4,31
Q9UHQ9	CYB5R1	NADH-cytochrome b5 reductase 1	4,31
Q96CX2	KCTD12	BTB/POZ domain-containing protein KCTD12	4,31
O75955	FLOT1	Flotillin-1	4,30
O43684	BUB3	Mitotic checkpoint protein BUB3	4,28
A0A7I2YQC0	NPM1	Nucleophosmin	4,28
O94851	MICAL2	[F-actin]-monooxygenase MICAL2	4,28
A0A0D9SF53	DDX3X	RNA helicase	4,28
Q9NQR4	NIT2	Omega-amidase NIT2	4,28
Q13045	FLII	Protein flightless-1 homolog	4,26
E7EX44	CALD1	Caldesmon 1	4,26
A0A2R8YG02	MSH2	DNA mismatch repair protein	4,26
O75063	FAM20B	Glycosaminoglycan xylosylkinase	4,25
Q96QR8	PURB	Transcriptional activator protein Pur-beta	4,22
Q53GQ0	HSD17B12	Very-long-chain 3-oxoacyl-CoA reductase	4,19
Q96EY7	PTCD3	Small ribosomal subunit protein mS39	4,18
Q8NBJ5	COLGALT1	Procollagen galactosyltransferase 1	4,18
Q92692	NECTIN2	Nectin-2	4,18
P36551	CPOX	Oxygen-dependent coproporphyrinogen-III oxidase, mitochondrial	4,17
P10253	GAA	Lysosomal alpha-glucosidase	4,16
Q8WWM7	ATXN2L	Ataxin-2-like protein	4,14
A0A8V8TLM6	TFRC	Transferrin receptor protein 1	4,13
P16435	POR	NADPH--cytochrome P450 reductase	4,13
P61978	HNRNPK	Heterogeneous nuclear ribonucleoprotein K	4,12
Q86UP2	KTN1	Kinectin	4,11
Q9NUJ1	ABHD10	Palmitoyl-protein thioesterase ABHD10, mitochondrial	4,11
A0A994J6K9	NCKAP1	NCK associated protein 1	4,09
O75436	VPS26A	Vacuolar protein sorting-associated protein 26A	4,09
P55884	EIF3B	Eukaryotic translation initiation factor 3 subunit B	4,06
Q9Y624	F11R	Junctional adhesion molecule A	4,06
Q9H845	ACAD9	Complex I assembly factor ACAD9, mitochondrial	4,06
P12532	CKMT1A	Creatine kinase U-type, mitochondrial	4,04
A3KFL5	EXOSC2	Ribosomal RNA-processing protein 4	4,00
Q9H0J9	PARP12	Protein mono-ADP-ribosyltransferase PARP12	3,99
Q6NUM9	RETSAT	All-trans-retinol 13,14-reductase	3,98
A0A8I5QKQ8	SPTLC1	Serine palmitoyltransferase long chain base subunit 1	3,95
G3V1L9	TJP1	Tight junction protein 1	3,94
A0A8V8TP22	WDR1	WD repeat domain 1	3,94
A0A7P0TAT8	HSP90B1	Heat shock protein 90 beta family member 1	3,93
Q9BRR6	ADPGK	ADP-dependent glucokinase	3,92
P06756	ITGAV	Integrin alpha-V	3,91
Q9UJA5	TRMT6	tRNA (adenine(58)-N(1))-methyltransferase non-catalytic subunit TRM6	3,91
H3BQK9	MACF1	Microtubule actin crosslinking factor 1	3,91
P17480	UBTF	Nucleolar transcription factor 1	3,89
Q9BTT6	LRRC1	Leucine-rich repeat-containing protein 1	3,89
Q9HDC9	APMAP	Adipocyte plasma membrane-associated protein	3,89
P05121	SERPINE1	Plasminogen activator inhibitor 1	3,88
Q96DA0	ZG16B	Zymogen granule protein 16 homolog B	3,87
P82675	MRPS5	Small ribosomal subunit protein uS5m	3,86
Q7Z434	MAVS	Mitochondrial antiviral-signaling protein	3,85
B0QY89	EIF3L	Eukaryotic translation initiation factor 3 subunit L	3,84
P55072	VCP	Transitional endoplasmic reticulum ATPase	3,81
Q5VT79	ANXA8L1	Annexin A8-like protein 1	3,80
J3KQJ1	SUMF2	Sulfatase modifying factor 2	3,80
Q16630	CPSF6	Cleavage and polyadenylation specificity factor subunit 6	3,77
P14923	JUP	Junction plakoglobin	3,76
P55081	MFAP1	Microfibrillar-associated protein 1	3,76
A0A5F9ZA98	ABCB7	ATP binding cassette subfamily B member 7	3,72
P54652	HSPA2	Heat shock-related 70 kDa protein 2	3,72
P27797	CALR	Calreticulin	3,70
P13473	LAMP2	Lysosome-associated membrane glycoprotein 2	3,70
A0A7P0TAE9	CANX	Calnexin	3,69
Q14152	EIF3A	Eukaryotic translation initiation factor 3 subunit A	3,69
O15027	SEC16A	Protein transport protein Sec16A	3,68
Q9UNM6	PSMD13	26S proteasome non-ATPase regulatory subunit 13	3,68
O14672	ADAM10	Disintegrin and metalloproteinase domain-containing protein 10	3,68
Q9Y2P8	RCL1	RNA 3'-terminal phosphate cyclase-like protein	3,67
H3BTS6	TMEM87A	Transmembrane protein 87A	3,64
Q7Z417	NUFIP2	FMR1-interacting protein NUFIP2	3,64
P47895	ALDH1A3	Retinaldehyde dehydrogenase 3	3,63
A0A7P0T8C7	SNX9	Sorting nexin 9	3,63
C9J9C1	PPP2R1A	Protein phosphatase 2 scaffold subunit Aalpha	3,62
Q99805	TM9SF2	Transmembrane 9 superfamily member 2	3,62
P04792	HSPB1	Heat shock protein beta-1	3,61
P28482	MAPK1	Mitogen-activated protein kinase 1	3,60
O43741	PRKAB2	5'-AMP-activated protein kinase subunit beta-2	3,59
M0QXB5	ETHE1	ETHE1 persulfide dioxygenase	3,59
A0A8V8TM48	RHOT2	Mitochondrial Rho GTPase	3,58
O00469	PLOD2	Procollagen-lysine,2-oxoglutarate 5-dioxygenase 2	3,57
A0A2R8Y891	PFKM	ATP-dependent 6-phosphofructokinase	3,56
B0YIW6	ARCN1	Coatomer subunit delta	3,55
Q13895	BYSL	Bystin	3,55
E7EUU4	EIF4G1	Eukaryotic translation initiation factor 4 gamma 1	3,55
B0QZ18	CPNE1	Copine 1	3,55
Q9UNF1	MAGED2	Melanoma-associated antigen D2	3,54
P01889	HLA-B	HLA class I histocompatibility antigen, B alpha chain	3,54
Q7L576	CYFIP1	Cytoplasmic FMR1-interacting protein 1	3,54
O95817	BAG3	BAG family molecular chaperone regulator 3	3,54
O75821	EIF3G	Eukaryotic translation initiation factor 3 subunit G	3,53
O43390	HNRNPR	Heterogeneous nuclear ribonucleoprotein R	3,53
Q96NY8	NECTIN4	Nectin-4	3,52
Q9Y5A9	YTHDF2	YTH domain-containing family protein 2	3,52
P22626	HNRNPA2B1	Heterogeneous nuclear ribonucleoproteins A2/B1	3,52
P22223	CDH3	Cadherin-3	3,51
Q9H936	SLC25A22	Mitochondrial glutamate carrier 1	3,51
P61254	RPL26	Large ribosomal subunit protein uL24	3,51
A0A499FIZ0	WDR26	WD repeat domain 26	3,51
P07954	FH	Fumarate hydratase, mitochondrial	3,51
Q8WUX1	SLC38A5	Sodium-coupled neutral amino acid transporter 5	3,50
D6RBW1	EIF4E	Eukaryotic translation initiation factor 4E	3,50
P17812	CTPS1	CTP synthase 1	3,49
A0A2R8Y7H4	PRPS1	Ribose-phosphate pyrophosphokinase	3,49
Q9UQ80	PA2G4	Proliferation-associated protein 2G4	3,49
Q8WVX9	FAR1	Fatty acyl-CoA reductase 1	3,49
P31947	SFN	14-3-3 protein sigma	3,49
P08648	ITGA5	Integrin alpha-5	3,49
Q96J02	ITCH	E3 ubiquitin-protein ligase Itchy homolog	3,48
Q969X5	ERGIC1	Endoplasmic reticulum-Golgi intermediate compartment protein 1	3,48
Q9BRQ8	AIFM2	Ferroptosis suppressor protein 1	3,48
Q5VYS8	TUT7	Terminal uridylyltransferase 7	3,48
Q8NEZ5	FBXO22	F-box only protein 22	3,48
P33992	MCM5	DNA replication licensing factor MCM5	3,47
A0A8V8TPC7	ERBIN	Erbb2 interacting protein	3,47
P53618	COPB1	Coatomer subunit beta	3,45
F2Z2W7	TRMT2A	tRNA (uracil(54)-C(5))-methyltransferase	3,40
Q14683	SMC1A	Structural maintenance of chromosomes protein 1A	3,40
Q9BTV4	TMEM43	Transmembrane protein 43	3,39
P78371	CCT2	T-complex protein 1 subunit beta	3,39
C9JH19	CTSD	Cathepsin D	3,37
P35613	BSG	Basigin	3,36
P16401	H1-5	Histone H1.5	3,36
Q07666	KHDRBS1	KH domain-containing, RNA-binding, signal transduction-associated protein 1	3,36
Q00587	CDC42EP1	Cdc42 effector protein 1	3,35
A0A994J5J1	RPN2	Dolichyl-diphosphooligosaccharide--protein glycosyltransferase subunit 2	3,34
Q5K651	SAMD9	Sterile alpha motif domain-containing protein 9	3,33
P51659	HSD17B4	Peroxisomal multifunctional enzyme type 2	3,32
A0A1W2PRS1	SCARB2	Scavenger receptor class B member 2 (Fragment)	3,31
P17301	ITGA2	Integrin alpha-2	3,31
A0A8V8TLA4	RPSA	40S ribosomal protein SA	3,30
Q6Q0C0	TRAF7	E3 ubiquitin-protein ligase TRAF7	3,30
Q04695	KRT17	Keratin, type I cytoskeletal 17	3,30
Q9H2U1	DHX36	ATP-dependent DNA/RNA helicase DHX36	3,29
Q08722	CD47	Leukocyte surface antigen CD47	3,27
O95573	ACSL3	Fatty acid CoA ligase Acsl3	3,26
A0A0A0MTI1	DHCR24	24-dehydrocholesterol reductase	3,26
Q9UKV8	AGO2	Protein argonaute-2	3,25
O00483	NDUFA4	Cytochrome c oxidase subunit NDUFA4	3,24
Q9Y5M8	SRPRB	Signal recognition particle receptor subunit beta	3,24
Q01650	SLC7A5	Large neutral amino acids transporter small subunit 1	3,24
P04259	KRT6B	Keratin, type II cytoskeletal 6B	3,23
E9PL19	PUF60	Poly(U)-binding-splicing factor PUF60	3,23
Q9NRG9	AAAS	Aladin	3,22
P42285	MTREX	Exosome RNA helicase MTR4	3,21
O15427	SLC16A3	Monocarboxylate transporter 4	3,21
P12830	CDH1	Cadherin-1	3,20
X6RAY8	MRPL4	Mitochondrial ribosomal protein L4	3,20
P53367	ARFIP1	Arfaptin-1	3,19
P22695	UQCRC2	Cytochrome b-c1 complex subunit 2, mitochondrial	3,19
O15270	SPTLC2	Serine palmitoyltransferase 2	3,18
P18031	PTPN1	Tyrosine-protein phosphatase non-receptor type 1	3,17
A0A8V8TQJ0	MSH6	DNA mismatch repair protein	3,16
P05455	SSB	Lupus La protein	3,16
H0Y8C6	IPO5	Importin 5 (Fragment)	3,16
P0DUB6	AMY1A	Alpha-amylase 1A	3,15
Q96FW1	OTUB1	Ubiquitin thioesterase OTUB1	3,15
Q86VP6	CAND1	Cullin-associated NEDD8-dissociated protein 1	3,15
Q14974	KPNB1	Importin subunit beta-1	3,11
H0Y5K5	ERGIC3	Endoplasmic reticulum-Golgi intermediate compartment protein (Fragment)	3,11
G5EA30	CELF1	CUG triplet repeat, RNA binding protein 1, isoform CRA_c	3,11
P30084	ECHS1	Enoyl-CoA hydratase, mitochondrial	3,11
A0A024R4E5	HDLBP	High density lipoprotein binding protein	3,10
Q99714	HSD17B10	3-hydroxyacyl-CoA dehydrogenase type-2	3,10
P13639	EEF2	Elongation factor 2	3,09
P01876	IGHA1	Immunoglobulin heavy constant alpha 1	3,09
C9J0Y3	GOLGA4	Golgin A4	3,06
H7BYY1	TPM1	Tropomyosin 1	3,06
E9PHZ5	CTSB	Cathepsin B	3,06
O60488	ACSL4	Long-chain-fatty-acid--CoA ligase 4	3,06
P07910	HNRNPC	Heterogeneous nuclear ribonucleoproteins C1/C2	3,06
Q14498	RBM39	RNA-binding protein 39	3,05
Q6UXN9	WDR82	WD repeat-containing protein 82	3,04
Q9Y2Z4	YARS2	Tyrosine--tRNA ligase, mitochondrial	3,04
Q92597	NDRG1	Protein NDRG1	3,03
Q9ULS5	TMCC3	Transmembrane and coiled-coil domain protein 3	3,03
P62249	RPS16	Small ribosomal subunit protein uS9	3,03
P00533	EGFR	Epidermal growth factor receptor	3,00
Q6P2E9	EDC4	Enhancer of mRNA-decapping protein 4	3,00
P50402	EMD	Emerin	2,99
Q15436	SEC23A	Protein transport protein Sec23A	2,99
Q1KMD3	HNRNPUL2	Heterogeneous nuclear ribonucleoprotein U-like protein 2	2,99
Q13148	TARDBP	TAR DNA-binding protein 43	2,99
O00154	ACOT7	Cytosolic acyl coenzyme A thioester hydrolase	2,98
H0YDT6	EIF3F	Eukaryotic translation initiation factor 3 subunit F	2,98
P05023	ATP1A1	Sodium/potassium-transporting ATPase subunit alpha-1	2,98
Q14165	MLEC	Malectin	2,97
O43615	TIMM44	Mitochondrial import inner membrane translocase subunit TIM44	2,97
A0A0J9YWL0	CRYBG1	Crystallin beta-gamma domain containing 1	2,97
Q96I99	SUCLG2	Succinate--CoA ligase [GDP-forming] subunit beta, mitochondrial	2,97
P30101	PDIA3	Protein disulfide-isomerase A3	2,96
A0A6Q8PFY7	TFG	Trafficking from ER to golgi regulator	2,96
P27701	CD82	CD82 antigen	2,95
Q6NUK1	SLC25A24	Mitochondrial adenyl nucleotide antiporter SLC25A24	2,95
P11142	HSPA8	Heat shock cognate 71 kDa protein	2,95
Q9Y6X9	MORC2	ATPase MORC2	2,94
E9PF18	HADH	Hydroxyacyl-CoA dehydrogenase	2,94
E9PL17	CLP1	Polyribonucleotide 5'-hydroxyl-kinase Clp1	2,94
Q15366	PCBP2	Poly(rC)-binding protein 2	2,92
Q8IZP0	ABI1	Abl interactor 1	2,92
Q8IVF7	FMNL3	Formin-like protein 3	2,91
O75323	NIPSNAP2	Protein NipSnap homolog 2	2,89
Q71RC2	LARP4	La-related protein 4	2,88
Q9Y6E0	STK24	Serine/threonine-protein kinase 24	2,88
Q99460	PSMD1	26S proteasome non-ATPase regulatory subunit 1	2,88
Q5T749	KPRP	Keratinocyte proline-rich protein	2,87
Q12931	TRAP1	Heat shock protein 75 kDa, mitochondrial	2,87
H0YLU7	ETFA	Electron transfer flavoprotein subunit alpha	2,87
P36542	ATP5F1C	ATP synthase subunit gamma, mitochondrial	2,86
A0A8I5KZB3	RAB3GAP2	RAB3 GTPase activating non-catalytic protein subunit 2	2,86
P38117	ETFB	Electron transfer flavoprotein subunit beta	2,86
Q96EY1	DNAJA3	DnaJ homolog subfamily A member 3, mitochondrial	2,84
Q99536	VAT1	Synaptic vesicle membrane protein VAT-1 homolog	2,84
P39019	RPS19	Small ribosomal subunit protein eS19	2,83
P42167	TMPO	Lamina-associated polypeptide 2, isoforms beta/gamma	2,83
O95429	BAG4	BAG family molecular chaperone regulator 4	2,83
O43242	PSMD3	26S proteasome non-ATPase regulatory subunit 3	2,82
Q8IY37	DHX37	Probable ATP-dependent RNA helicase DHX37	2,80
Q6P158	DHX57	Putative ATP-dependent RNA helicase DHX57	2,80
A0A087X1N8	SERPINB6	Serpin family B member 6	2,80
Q9BXP5	SRRT	Serrate RNA effector molecule homolog	2,79
P01040	CSTA	Cystatin-A	2,79
P63244	RACK1	Small ribosomal subunit protein RACK1	2,78
Q96DZ1	ERLEC1	Endoplasmic reticulum lectin 1	2,78
A0A0G2JIW1	HSPA1B	Heat shock 70 kDa protein 1B	2,78
B7Z4C8	RPL31	60S ribosomal protein L31	2,78
P43304	GPD2	Glycerol-3-phosphate dehydrogenase, mitochondrial	2,77
Q9P0L0	VAPA	Vesicle-associated membrane protein-associated protein A	2,77
O75940	SMNDC1	Survival of motor neuron-related-splicing factor 30	2,77
Q8N6T3	ARFGAP1	ADP-ribosylation factor GTPase-activating protein 1	2,77
Q9P2B2	PTGFRN	Prostaglandin F2 receptor negative regulator	2,75
Q9BS26	ERP44	Endoplasmic reticulum resident protein 44	2,75
Q9NZL4	HSPBP1	Hsp70-binding protein 1	2,74
Q92520	FAM3C	Protein FAM3C	2,74
A0A494C039	HYOU1	Hypoxia up-regulated 1	2,72
P62861	FAU	Ubiquitin-like FUBI-ribosomal protein eS30 fusion protein	2,71
P20042	EIF2S2	Eukaryotic translation initiation factor 2 subunit 2	2,70
Q92542	NCSTN	Nicastrin	2,70
P13995	MTHFD2	Bifunctional methylenetetrahydrofolate dehydrogenase/cyclohydrolase, mitochondrial	2,70
P00367	GLUD1	Glutamate dehydrogenase 1, mitochondrial	2,69
P61626	LYZ	Lysozyme C	2,68
P20591	MX1	Interferon-induced GTP-binding protein Mx1	2,68
O43395	PRPF3	U4/U6 small nuclear ribonucleoprotein Prp3	2,67
P61513	RPL37A	Large ribosomal subunit protein eL43	2,67
P09661	SNRPA1	U2 small nuclear ribonucleoprotein A'	2,66
P35250	RFC2	Replication factor C subunit 2	2,66
Q6KB66	KRT80	Keratin, type II cytoskeletal 80	2,66
J3KTL2	SRSF1	Serine/arginine-rich splicing factor 1	2,65
Q13200	PSMD2	26S proteasome non-ATPase regulatory subunit 2	2,65
Q8IUF8	RIOX2	Ribosomal oxygenase 2	2,64
Q9HCY8	S100A14	Protein S100-A14	2,64
Q08380	LGALS3BP	Galectin-3-binding protein	2,63
P25398	RPS12	Small ribosomal subunit protein eS12	2,63
P99999	CYCS	Cytochrome c	2,63
O00231	PSMD11	26S proteasome non-ATPase regulatory subunit 11	2,62
A0A7P0T9C4	DYNC1H1	Dynein cytoplasmic 1 heavy chain 1	2,62
O95433	AHSA1	Activator of 90 kDa heat shock protein ATPase homolog 1	2,62
A0A0C4DG49	PVR	PVR cell adhesion molecule	2,62
P61353	RPL27	Large ribosomal subunit protein eL27	2,61
P56192	MARS1	Methionine--tRNA ligase, cytoplasmic	2,61
P17655	CAPN2	Calpain-2 catalytic subunit	2,60
P02538	KRT6A	Keratin, type II cytoskeletal 6A	2,59
Q15904	ATP6AP1	V-type proton ATPase subunit S1	2,58
Q96PM9	ZNF385A	Zinc finger protein 385A	2,58
P49915	GMPS	GMP synthase [glutamine-hydrolyzing]	2,58
P25789	PSMA4	Proteasome subunit alpha type-4	2,57
P62136	PPP1CA	Serine/threonine-protein phosphatase PP1-alpha catalytic subunit	2,57
Q9H3P7	ACBD3	Golgi resident protein GCP60	2,57
P16402	H1-3	Histone H1.3	2,57
A0A7P0Z4P5	SLC3A2	Solute carrier family 3 member 2	2,56
Q9ULV0	MYO5B	Unconventional myosin-Vb	2,55
P41252	IARS1	Isoleucine--tRNA ligase, cytoplasmic	2,54
P21397	MAOA	Amine oxidase [flavin-containing] A	2,53
Q9Y6Y8	SEC23IP	SEC23-interacting protein	2,53
O43660	PLRG1	Pleiotropic regulator 1	2,52
Q9H5V8	CDCP1	CUB domain-containing protein 1	2,52
A0A8V8TMZ5	SEC61A1	SEC61 translocon subunit alpha 1	2,50
P62851	RPS25	Small ribosomal subunit protein eS25	2,49
H7C5A2	EMC1	ER membrane protein complex subunit 1	2,48
P55010	EIF5	Eukaryotic translation initiation factor 5	2,48
E7ETY2	TCOF1	Treacle ribosome biogenesis factor 1	2,47
Q92896	GLG1	Golgi apparatus protein 1	2,47
A0A8V8TQ44	PSMD8	26S proteasome non-ATPase regulatory subunit 8	2,47
Q5T4S7	UBR4	E3 ubiquitin-protein ligase UBR4	2,47
O75822	EIF3J	Eukaryotic translation initiation factor 3 subunit J	2,46
I1E4Y6	GIGYF2	GRB10 interacting GYF protein 2	2,46
Q96HC4	PDLIM5	PDZ and LIM domain protein 5	2,46
Q9BZK7	TBL1XR1	F-box-like/WD repeat-containing protein TBL1XR1	2,45
Q8TER5	ARHGEF40	Rho guanine nucleotide exchange factor 40	2,44
P59665	DEFA1	Neutrophil defensin 1	2,44
P05026	ATP1B1	Sodium/potassium-transporting ATPase subunit beta-1	2,43
O75694	NUP155	Nuclear pore complex protein Nup155	2,43
P16070	CD44	CD44 antigen	2,42
G3V325	ATP5MF-PTCD1	ATP5MF-PTCD1 readthrough	2,42
Q9NZN4	EHD2	EH domain-containing protein 2	2,42
Q8N2K0	ABHD12	Lysophosphatidylserine lipase ABHD12	2,41
Q52LJ0	FAM98B	Protein FAM98B	2,41
P31040	SDHA	Succinate dehydrogenase [ubiquinone] flavoprotein subunit, mitochondrial	2,40
P29350	PTPN6	Tyrosine-protein phosphatase non-receptor type 6	2,40
A0A8I5KW61	3 SV	C-X-C motif chemokine	2,39
P11172	UMPS	Uridine 5'-monophosphate synthase	2,39
O94826	TOMM70	Mitochondrial import receptor subunit TOM70	2,39
O95292	VAPB	Vesicle-associated membrane protein-associated protein B/C	2,38
O00487	PSMD14	26S proteasome non-ATPase regulatory subunit 14	2,38
O15400	STX7	Syntaxin-7	2,37
P31944	CASP14	Caspase-14	2,37
Q92544	TM9SF4	Transmembrane 9 superfamily member 4	2,36
Q8WV24	PHLDA1	Pleckstrin homology-like domain family A member 1	2,36
O95347	SMC2	Structural maintenance of chromosomes protein 2	2,35
P08238	HSP90AB1	Heat shock protein HSP 90-beta	2,35
P25705	ATP5F1A	ATP synthase subunit alpha, mitochondrial	2,33
P09497	CLTB	Clathrin light chain B	2,32
P39656	DDOST	Dolichyl-diphosphooligosaccharide--protein glycosyltransferase 48 kDa subunit	2,32
Q9H6S0	YTHDC2	3'-5' RNA helicase YTHDC2	2,32
O43752	STX6	Syntaxin-6	2,32
P13489	RNH1	Ribonuclease inhibitor	2,31
P55209	NAP1L1	Nucleosome assembly protein 1-like 1	2,31
P07384	CAPN1	Calpain-1 catalytic subunit	2,31
O00391	QSOX1	Sulfhydryl oxidase 1	2,31
Q16610	ECM1	Extracellular matrix protein 1	2,31
Q9NXC5	MIOS	GATOR complex protein MIOS	2,30
P42677	RPS27	Small ribosomal subunit protein eS27	2,28
A0A8V8TRB3	PPP2CA	Serine/threonine-protein phosphatase	2,28
Q9Y277	VDAC3	Voltage-dependent anion-selective channel protein 3	2,26
P49257	LMAN1	Protein ERGIC-53	2,26
Q9UN37	VPS4A	Vacuolar protein sorting-associated protein 4A	2,25
A0A087X1Z3	PSME2	Proteasome activator subunit 2	2,25
P15924	DSP	Desmoplakin	2,25
P26038	MSN	Moesin	2,25
A0A494C0J7	3 SV	Transglutaminase-like domain-containing protein	2,24
P18077	RPL35A	Large ribosomal subunit protein eL33	2,22
Q96PK6	RBM14	RNA-binding protein 14	2,21
A0A804HL12	MYCBP2	RCR-type E3 ubiquitin transferase	2,20
Q7L1Q6	BZW1	eIF5-mimic protein 2	2,20
A0A3B3IUB5	HM13	Histocompatibility minor 13	2,18
Q9BUL8	PDCD10	Programmed cell death protein 10	2,18
Q93008	USP9X	Probable ubiquitin carboxyl-terminal hydrolase FAF-X	2,17
A0A7P0TA71	P4HB	Protein disulfide-isomerase	2,17
Q9Y224	RTRAF	RNA transcription, translation and transport factor protein	2,16
P54709	ATP1B3	Sodium/potassium-transporting ATPase subunit beta-3	2,15
Q99873	PRMT1	Protein arginine N-methyltransferase 1	2,14
Q01130	SRSF2	Serine/arginine-rich splicing factor 2	2,14
P04083	ANXA1	Annexin A1	2,14
O14734	ACOT8	Acyl-coenzyme A thioesterase 8	2,13
G8JLB6	HNRNPH1	Heterogeneous nuclear ribonucleoprotein H1	2,13
Q96RS6	NUDCD1	NudC domain-containing protein 1	2,13
B7Z6Z4	MYL6	Myosin light chain 6	2,12
O14980	XPO1	Exportin-1	2,12
P62277	RPS13	Small ribosomal subunit protein uS15	2,12
F8WF69	CLTA	Clathrin light chain	2,11
P62269	RPS18	Small ribosomal subunit protein uS13	2,11
P28062	PSMB8	Proteasome subunit beta type-8	2,10
P52907	CAPZA1	F-actin-capping protein subunit alpha-1	2,10
Q99614	TTC1	Tetratricopeptide repeat protein 1	2,09
U3KQV3	4 SV	PPM-type phosphatase domain-containing protein (Fragment)	2,08
P15529	CD46	Membrane cofactor protein	2,07
A0A8V8TMY4	PFKP	ATP-dependent 6-phosphofructokinase	2,06
Q96QK1	VPS35	Vacuolar protein sorting-associated protein 35	2,06
Q86WR7	PROSER2	Proline and serine-rich protein 2	2,06
P09543	CNP	2',3'-cyclic-nucleotide 3'-phosphodiesterase	2,05
Q7Z2K6	ERMP1	Endoplasmic reticulum metallopeptidase 1	2,05
P04264	KRT1	Keratin, type II cytoskeletal 1	2,05
P10809	HSPD1	60 kDa heat shock protein, mitochondrial	2,01
A0A0A6YYJ8	LUC7L2	Putative RNA-binding protein Luc7-like 2	2,01
O60884	DNAJA2	DnaJ homolog subfamily A member 2	2,01
P09622	DLD	Dihydrolipoyl dehydrogenase, mitochondrial	2,00
Q07021	C1QBP	Complement component 1 Q subcomponent-binding protein, mitochondrial	2,00
Q9Y4W6	AFG3L2	AFG3-like protein 2	2,00
Q14247	CTTN	Src substrate cortactin	2,00

Supplemental table 4 : Summary table of proteins present in NIH-3T3-Src-Tks5-GFP cells seeded on plastic identified by mass spectrometry analysis of Tks5 interactome

Accession	Gene Name	Description	Abundance Ratio: (TKS5) / (Control
O89032	SH3PXD2A	SH3 and PX domain-containing protein 2A	253,58
P67871	CSNK2B	Casein kinase II subunit beta	55,49
O35682	MYADM	Myeloid-associated differentiation marker	30,47
O35674	ADAM19	Disintegrin and metalloproteinase domain-containing protein 19	28,37
A0A0G2JGS4	CAMK2D	calcium/calmodulin-dependent protein kinase	28,11
O88839	ADAM15	Disintegrin and metalloproteinase domain-containing protein 15	21,39
P51125	CAST	Calpastatin	18,81
E9QAS8	FMR1	Fragile X messenger ribonucleoprotein 1	18,07
Q80SY4	MIB1	E3 ubiquitin-protein ligase MIB1	16,10
O88477	IGF2BP1	Insulin-like growth factor 2 mRNA-binding protein 1	16,10
Q9JM51	PTGES	Prostaglandin E synthase	15,95
P61514	RPL37A	Large ribosomal subunit protein eL43	15,70
P84091	AP2M1	AP-2 complex subunit mu	15,01
O70251	EEF1B	Elongation factor 1-beta	14,48
P84104	SRSF3	Serine/arginine-rich splicing factor 3	14,13
Q9WVE8	PACSIN2	Protein kinase C and casein kinase substrate in neurons protein 2	13,43
Q5SF07	IGF2BP2	Insulin-like growth factor 2 mRNA-binding protein 2	13,30
Q91Z31	PTBP2	Polypyrimidine tract-binding protein 2	12,57
Q3TLP8	RAC1	Rac family small GTPase 1	12,35
A0A2R8VK70	CD47	Leukocyte surface antigen CD47	12,32
Q9D1R9	RPL34	Large ribosomal subunit protein eL34	12,06
Q9QXT0	CNPY2	Protein canopy homolog 2	11,68
P22907	HMBS	Porphobilinogen deaminase	11,55
D3YW42	CCDC9	Coiled-coil domain containing 9	11,34
A0A0R4J008	HDAC2	Histone deacetylase 2	11,16
Q9CQI7	SNRPB2	U2 small nuclear ribonucleoprotein B''	11,14
A0A991EKZ2	FXR2	FMR1 autosomal homolog 2	10,88
Q9EPU0	UPF1	Regulator of nonsense transcripts 1	10,75
Q6PAC3	DCAF13	DDB1- and CUL4-associated factor 13	10,52
Q64337	SQSTM1	Sequestosome-1	10,51
Q8VE70	PDCD10	Programmed cell death protein 10	10,22
P70372	ELAVL1	ELAV-like protein 1	10,17
A0A0N4SUH8	NFU1	NFU1 iron-sulfur cluster scaffold homolog, mitochondrial	10,12
Q8JZX4	RBM17	Splicing factor 45	9,95
Q5EG47	PRKAA1	5'-AMP-activated protein kinase catalytic subunit alpha-1	9,95
Q9CXI5	MANF	Mesencephalic astrocyte-derived neurotrophic factor	9,82
Q9JJ89	CCDC86	Coiled-coil domain-containing protein 86	9,54
Q9R078	PRKAB1	5'-AMP-activated protein kinase subunit beta-1	9,50
Q8BK67	RCC2	Protein RCC2	9,30
E9PUK3	FNBP1L	Formin-binding protein 1-like	9,18
Q99PA7	MAGEA13	MAGE family member A13	8,99
Q64433	HSPE1	10 kDa heat shock protein, mitochondrial	8,96
Q9JHI7	EXOSC9	Exosome complex component RRP45	8,86
Q8C1D8	IWS1	Protein IWS1 homolog	8,85
Q9JI13	UTP3	Something about silencing protein 10	8,83
O08547	SEC22B	Vesicle-trafficking protein SEC22b	8,63
P52734	FGD1	FYVE, RhoGEF and PH domain-containing protein 1	8,38
Q9QUJ7	ACSL4	Long-chain-fatty-acid--CoA ligase 4	8,22
O54950	PRKAG1	5'-AMP-activated protein kinase subunit gamma-1	8,20
P97461	RPS5	Small ribosomal subunit protein uS7	8,06
P24788	CDK11B	Cyclin-dependent kinase 11B	8,00
Q8VCH8	UBXN4	UBX domain-containing protein 4	7,86
Q9CPN8	IGF2BP3	Insulin-like growth factor 2 mRNA-binding protein 3	7,84
Q80UM7	MOGS	Mannosyl-oligosaccharide glucosidase	7,84
P00493	HPRT1	Hypoxanthine-guanine phosphoribosyltransferase	7,68
Q9WTX2	PRKRA	Interferon-inducible double-stranded RNA-dependent protein kinase activator A	7,60
P67984	RPL22	Large ribosomal subunit protein eL22	7,55
Q6ZWU9	RPS27	Small ribosomal subunit protein eS27	7,34
Q8BJ71	NUP93	Nuclear pore complex protein Nup93	7,34
D3Z722	RPS19	40S ribosomal protein S19	7,27
P35279	RAB6A	Ras-related protein Rab-6A	7,02
Q9R190	MTA2	Metastasis-associated protein MTA2	6,97
P12265	GUSB	Beta-glucuronidase	6,81
P16045	LGALS1	Galectin-1	6,66
Q4FZC9	SYNE3	Nesprin-3	6,57
Q91YS8	CAMK1	Calcium/calmodulin-dependent protein kinase type 1	6,57
Q9D3E6	STAG1	Cohesin subunit SA-1	6,49
P17426	AP2A1	AP-2 complex subunit alpha-1	6,49
Q8BX10	PGAM5	Serine/threonine-protein phosphatase PGAM5, mitochondrial	6,46
A0A5F8MPP7	SH3PXD2B	SH3 and PX domains 2B	6,43
P68254	YWHAQ	14-3-3 protein theta	6,38
Q8K4Q8	COLEC12	Collectin-12	6,19
Q8WTY4	CIAPIN1	Anamorsin	6,17
P84099	RPL19	Large ribosomal subunit protein eL19	6,06
O55126	NIPSNAP2	Protein NipSnap homolog 2	6,05
Q9ES97	RTN3	Reticulon-3	6,04
Q9ERB0	SNAP29	Synaptosomal-associated protein 29	6,04
P47964	RPL36	Large ribosomal subunit protein eL36	5,96
Q8BL97	SRSF7	Serine/arginine-rich splicing factor 7	5,93
Q3UEB3	PUF60	Poly(U)-binding-splicing factor PUF60	5,89
Q8CCK0	MACROH2A2	Core histone macro-H2A.2	5,87
Q9D8E6	RPL4	Large ribosomal subunit protein uL4	5,76
Q569Z5	DDX46	Probable ATP-dependent RNA helicase DDX46	5,60
Q8BL66	EEA1	Early endosome antigen 1	5,57
Q7TMK9	SYNCRIP	Heterogeneous nuclear ribonucleoprotein Q	5,56
P15116	CDH2	Cadherin-2	5,52
Q91VK1	BZW2	eIF5-mimic protein 1	5,52
Q6PE01	SNRNP40	U5 small nuclear ribonucleoprotein 40 kDa protein	5,51
D3YYT1	GLYR1	Cytokine-like nuclear factor N-PAC	5,45
P86048	RPL10L	Large ribosomal subunit protein uL16-like	5,34
P29341	PABPC1	Polyadenylate-binding protein 1	5,34
Q8BYK6	YTHDF3	YTH domain-containing family protein 3	5,28
P68510	YWHAH	14-3-3 protein eta	5,27
P61982	YWHAG	14-3-3 protein gamma	5,25
Q61205	PAFAH1B3	Platelet-activating factor acetylhydrolase IB subunit alpha1	5,23
Q501J7	PHACTR4	Phosphatase and actin regulator 4	5,20
P35762	CD81	CD81 antigen	5,20
Q8R1B4	EIF3C	Eukaryotic translation initiation factor 3 subunit C	5,19
P27659	RPL3	Large ribosomal subunit protein uL3	5,19
P40240	CD9	CD9 antigen	5,19
P61027	RAB10	Ras-related protein Rab-10	5,17
Q8BSY0	ASPH	Aspartyl/asparaginyl beta-hydroxylase	5,16
P62855	RPS26	Small ribosomal subunit protein eS26	5,12
B2RRE7	OTUD4	OTU domain-containing protein 4	5,11
Q5F2E7	NUFIP2	FMR1-interacting protein NUFIP2	5,11
Q61335	BCAP31	B-cell receptor-associated protein 31	5,10
P47915	RPL29	Large ribosomal subunit protein eL29	5,09
P03975	IAP	IgE-binding protein	5,05
E9QAQ7	ARID1A	AT rich interactive domain 1A (SWI-like)	5,04
Q8BMJ2	LARS1	Leucine--tRNA ligase, cytoplasmic	5,03
Q9Z315	SART1	U4/U6.U5 tri-snRNP-associated protein 1	4,97
Q6PHQ9	PABPC4	Polyadenylate-binding protein	4,97
Q6PGL7	WASHC2	WASH complex subunit 2	4,95
P50543	S100A11	Protein S100-A11	4,94
P21995	EMB	Embigin	4,92
D3YZC9	SF1	Splicing factor 1	4,92
O35295	PURB	Transcriptional activator protein Pur-beta	4,91
Q3UL36	ARGLU1	Arginine and glutamate-rich protein 1	4,90
Q60631	GRB2	Growth factor receptor-bound protein 2	4,88
P63276	RPS17	Small ribosomal subunit protein eS17	4,87
Q9D0I8	MRTO4	mRNA turnover protein 4 homolog	4,80
P62889	RPL30	Large ribosomal subunit protein eL30	4,77
Q569Z6	THRAP3	Thyroid hormone receptor-associated protein 3	4,76
P50446	KRT6A	Keratin, type II cytoskeletal 6A	4,73
P10639	TXN	Thioredoxin	4,73
F6ZDS4	TPR	Nucleoprotein TPR	4,72
Q9R1J0	NSDHL	Sterol-4-alpha-carboxylate 3-dehydrogenase, decarboxylating	4,69
A0A991ENV6	SFPQ	Splicing factor proline/glutamine rich (polypyrimidine tract binding protein associated)	4,65
O54941	SMARCE1	SWI/SNF-related matrix-associated actin-dependent regulator of chromatin subfamily E member 1	4,59
A0A1D5RM85	RPL18A	Ribosomal protein L18A (Fragment)	4,54
A0A1B0GS68	GM45713	60S ribosomal protein L13a	4,51
Q8BXV2	BRI3BP	BRI3-binding protein	4,51
Q8VHE0	SEC63	Translocation protein SEC63 homolog	4,49
P54227	STMN1	Stathmin	4,49
P46638	RAB11B	Ras-related protein Rab-11B	4,44
Q9D3L3	SNAP23	Synaptosomal-associated protein	4,41
E9QA15	CALD1	Caldesmon 1	4,38
Q61490	ALCAM	CD166 antigen	4,37
Q91YN9	BAG2	BAG family molecular chaperone regulator 2	4,36
Q3THK3	GTF2F1	General transcription factor IIF subunit 1	4,35
Q8C2Q7	HNRNPH1	Heterogeneous nuclear ribonucleoprotein H1	4,35
Q8K1N4	SPATS2	Spermatogenesis-associated serine-rich protein 2	4,34
Q9DCE5	PAK1IP1	p21-activated protein kinase-interacting protein 1	4,34
Q91VX2	UBAP2	Ubiquitin-associated protein 2	4,30
Q5SWN2	RPA1	Replication protein A subunit	4,29
Q61576	FKBP10	Peptidyl-prolyl cis-trans isomerase FKBP10	4,29
Q9DAW6	PRPF4	U4/U6 small nuclear ribonucleoprotein Prp4	4,27
Q61584	FXR1	RNA-binding protein FXR1	4,27
P62071	RRAS2	Ras-related protein R-Ras2	4,26
P17427	AP2A2	AP-2 complex subunit alpha-2	4,25
P62259	YWHAE	14-3-3 protein epsilon	4,25
P97372	PSME2	Proteasome activator complex subunit 2	4,25
Q9CX86	HNRNPA0	Heterogeneous nuclear ribonucleoprotein A0	4,24
E9QA16	CALD1	Caldesmon 1	4,23
P62918	RPL8	Large ribosomal subunit protein uL2	4,22
Q9R020	ZRANB2	Zinc finger Ran-binding domain-containing protein 2	4,21
Q02013	AQP1	Aquaporin-1	4,21
Q7TNV0	DEK	Protein DEK	4,20
O08539	BIN1	Myc box-dependent-interacting protein 1	4,18
P14869	RPLP0	Large ribosomal subunit protein uL10	4,17
Q9QZD8	SLC25A10	Mitochondrial dicarboxylate carrier	4,17
Q8CG47	SMC4	Structural maintenance of chromosomes protein 4	4,16
Q5XJF6	RPL10A	Ribosomal protein	4,16
Q62189	SNRPA	U1 small nuclear ribonucleoprotein A	4,14
P62960	YBX1	Y-box-binding protein 1	4,13
Q9D0R4	DDX56	Probable ATP-dependent RNA helicase DDX56	4,12
A0A0R4J0Q5	LMNB2	Lamin B2	4,11
Q9QXX4	SLC25A13	Electrogenic aspartate/glutamate antiporter SLC25A13, mitochondrial	4,11
Q99K48	NONO	Non-POU domain-containing octamer-binding protein	4,06
P63242	EIF5A	Eukaryotic translation initiation factor 5A-1	4,06
P62869	ELOB	Elongin-B	4,04
Q9Z0L0	TPBG	Trophoblast glycoprotein	4,01
P56183	RRP1	Ribosomal RNA processing protein 1 homolog A	3,99
P26369	U2AF2	Splicing factor U2AF 65 kDa subunit	3,98
A2A9Q2	NRD1	Nardilysin, N-arginine dibasic convertase, NRD convertase 1	3,97
P17809	SLC2A1	Solute carrier family 2, facilitated glucose transporter member 1	3,96
Q8VBT6	APOBR	Apolipoprotein B receptor	3,96
P97855	G3BP1	Ras GTPase-activating protein-binding protein 1	3,96
A0A6Q6PUT5	PRPF40A	Pre-mRNA processing factor 40A (Fragment)	3,95
O35345	KPNA6	Importin subunit alpha-7	3,94
A2A5S3	STAU1	Staufen double-stranded RNA binding protein 1	3,90
Q62159	RHOC	Rho-related GTP-binding protein RhoC	3,88
P09671	SOD2	Superoxide dismutase [Mn], mitochondrial	3,86
A0A5F8MPS7	ACP2	Acid phosphatase 2, lysosomal	3,85
Q8VEK3	HNRNPU	Heterogeneous nuclear ribonucleoprotein U	3,85
Q9R0L6	PCM1	Pericentriolar material 1 protein	3,84
Q6ZQ58	LARP1	La-related protein 1	3,81
A0A286YDA2	NOLC1	Nucleolar and coiled-body phosphoprotein 1	3,80
Q9R0Y5	AK1	Adenylate kinase isoenzyme 1	3,80
P97379	G3BP2	Ras GTPase-activating protein-binding protein 2	3,80
Q922Q8	LRRC59	Leucine-rich repeat-containing protein 59	3,79
Q6P5H2	NES	Nestin	3,77
P62082	RPS7	Small ribosomal subunit protein eS7	3,74
A0A494B945	RAB1B	RAB1B, member RAS oncogene family (Fragment)	3,73
E9QB02	36951	Methionine--tRNA ligase, cytoplasmic	3,73
Q9D0E1	HNRNPM	Heterogeneous nuclear ribonucleoprotein M	3,72
Q9CWK3	CD2BP2	CD2 antigen cytoplasmic tail-binding protein 2	3,69
O35516	NOTCH2	Neurogenic locus notch homolog protein 2	3,68
P61750	ARF4	ADP-ribosylation factor 4	3,66
Q9DBR7	PPP1R12A	Protein phosphatase 1 regulatory subunit 12A	3,61
P31324	PRKAR2B	cAMP-dependent protein kinase type II-beta regulatory subunit	3,60
Q9CQV8	YWHAB	14-3-3 protein beta/alpha	3,60
Q62418	DBNL	Drebrin-like protein	3,59
Q9D2G2	DLST	Dihydrolipoyllysine-residue succinyltransferase component of 2-oxoglutarate dehydrogenase complex, mitochondrial	3,57
A0A1L1SSH9	SPARC	SPARC	3,57
Q9CZH7	MXRA7	Matrix-remodeling-associated protein 7	3,52
Q920A7	AFG3L1	AFG3-like protein 1	3,52
A0A0R4J0U2	PPP4R2	Protein phosphatase 4, regulatory subunit 2	3,52
P43274	H1-4	Histone H1.4	3,51
Q9WUK2	EIF4H	Eukaryotic translation initiation factor 4H	3,51
E9QAS5	CHD4	DNA helicase	3,51
P70349	HINT1	Adenosine 5'-monophosphoramidase HINT1	3,50
Q8BML9	QARS1	Glutamine--tRNA ligase	3,50
Q3UYV9	NCBP1	Nuclear cap-binding protein subunit 1	3,49
P62858	RPS28	Small ribosomal subunit protein eS28	3,49
Q9R002	IFI202	Interferon-activable protein 202	3,47
P12970	RPL7A	Large ribosomal subunit protein eL8	3,43
Q80X90	FLNB	Filamin-B	3,40
P42669	PURA	Transcriptional activator protein Pur-alpha	3,39
Q80UU9	PGRMC2	Membrane-associated progesterone receptor component 2	3,39
Q8BG15	CTDSPL2	CTD small phosphatase-like protein 2	3,38
Q6PFA2	CLTA	Clathrin light chain	3,38
P23116	EIF3A	Eukaryotic translation initiation factor 3 subunit A	3,33
Q3TLH4	PRRC2C	Protein PRRC2C	3,33
Q61937	NPM1	Nucleophosmin	3,33
P09103	P4HB	Protein disulfide-isomerase	3,32
P49722	PSMA2	Proteasome subunit alpha type-2	3,31
Q9Z0X1	AIFM1	Apoptosis-inducing factor 1, mitochondrial	3,31
Q9CQW9	IFITM3	Interferon-induced transmembrane protein 3	3,30
P63073	EIF4E	Eukaryotic translation initiation factor 4E	3,29
Q921F2	TARDBP	TAR DNA-binding protein 43	3,29
O35286	DHX15	ATP-dependent RNA helicase DHX15	3,29
Q9CQX2	CYB5B	Cytochrome b5 type B	3,28
Q8VDM6	HNRNPUL1	Heterogeneous nuclear ribonucleoprotein U-like protein 1	3,27
P08228	SOD1	Superoxide dismutase [Cu-Zn]	3,25
Q640M1	UTP14A	U3 small nucleolar RNA-associated protein 14 homolog A	3,25
O08583	ALYREF	THO complex subunit 4	3,25
Q91XV3	BASP1	Brain acid soluble protein 1	3,24
Q9CQD1	RAB5A	Ras-related protein Rab-5A	3,24
O09106	HDAC1	Histone deacetylase 1	3,24
P70399	TP53BP1	TP53-binding protein 1	3,23
E9PZC3	BLVRB	Biliverdin reductase B (flavin reductase (NADPH))	3,22
P68037	UBE2L3	Ubiquitin-conjugating enzyme E2 L3	3,21
Q9WVC3	CAV2	Caveolin-2	3,20
P35282	RAB21	Ras-related protein Rab-21	3,20
P35979	RPL12	Large ribosomal subunit protein uL11	3,19
O35841	API5	Apoptosis inhibitor 5	3,18
Q6P5D8	SMCHD1	Structural maintenance of chromosomes flexible hinge domain-containing protein 1	3,15
Q9Z2K1	KRT16	Keratin, type I cytoskeletal 16	3,15
G3UW86	PRC1	Protein regulator of cytokinesis 1	3,13
Q8BH97	RCN3	Reticulocalbin-3	3,13
Q6P8I4	PCNP	PEST proteolytic signal-containing nuclear protein	3,12
Q8CH25	SLTM	SAFB-like transcription modulator	3,11
Q6PDG5	SMARCC2	SWI/SNF complex subunit SMARCC2	3,08
Q99MR6	SRRT	Serrate RNA effector molecule homolog	3,06
Q8BFR4	GNS	N-acetylglucosamine-6-sulfatase	3,06
Q7TPV4	MYBBP1A	Myb-binding protein 1A	3,05
Q8CJF9	AGO3	Protein argonaute-3	3,05
P11438	LAMP1	Lysosome-associated membrane glycoprotein 1	3,05
Q8C266	RAB5C	RAB5C, member RAS oncogene family	3,04
Q91V41	RAB14	Ras-related protein Rab-14	3,03
Q920Q6	MSI2	RNA-binding protein Musashi homolog 2	3,02
Q9JIW9	RALB	Ras-related protein Ral-B	3,02
Q3TJD7	PDLIM7	PDZ and LIM domain protein 7	3,01
Q9D024	CCDC47	PAT complex subunit CCDC47	3,01
Q91YT0	NDUFV1	NADH dehydrogenase [ubiquinone] flavoprotein 1, mitochondrial	3,01
Q9QWH1	PHC2	Polyhomeotic-like protein 2	3,00
Q9Z0N1	EIF2S3X	Eukaryotic translation initiation factor 2 subunit 3, X-linked	3,00
Q6IRU5	CLTB	Clathrin light chain B	2,99
A0A2R8VK76	CPSF1	Cleavage and polyadenylation specific factor 1	2,99
A0A1W2P7A1	RPS12	40S ribosomal protein S12	2,99
Q3UMU9	HDGFL2	Hepatoma-derived growth factor-related protein 2	2,98
Q8BGT7	SMNDC1	Survival of motor neuron-related-splicing factor 30	2,97
P59999	ARPC4	Actin-related protein 2/3 complex subunit 4	2,96
Q20BD0	HNRNPAB	Heterogeneous nuclear ribonucleoprotein A/B	2,95
P62141	PPP1CB	Serine/threonine-protein phosphatase PP1-beta catalytic subunit	2,95
Q9R112	SQOR	Sulfide:quinone oxidoreductase, mitochondrial	2,93
H7BX95	SRSF1	Serine/arginine-rich splicing factor 1	2,93
Q8CEE7	RDH13	Retinol dehydrogenase 13	2,92
P63101	YWHAZ	14-3-3 protein zeta/delta	2,92
P55302	LRPAP1	Alpha-2-macroglobulin receptor-associated protein	2,91
A0A494BB95	EIF1A	Eukaryotic translation initiation factor 4C	2,91
Q9DC48	CDC40	Pre-mRNA-processing factor 17	2,90
Q8BLH7	HIRIP3	HIRA-interacting protein 3	2,90
Q64152	BTF3	Transcription factor BTF3	2,90
Q9JIH2	NUP50	Nuclear pore complex protein Nup50	2,89
H3BL37	TCOF1	Treacle ribosome biogenesis factor 1	2,89
Q9QXS6	DBN1	Drebrin	2,89
Q9CR57	RPL14	Large ribosomal subunit protein eL14	2,88
Q9CPV5	PMF1	Polyamine-modulated factor 1	2,88
Q8K019	BCLAF1	Bcl-2-associated transcription factor 1	2,86
Q9D8X2	CCDC124	Coiled-coil domain-containing protein 124	2,86
A0A1W2P7Q9	MYL6	Myosin, light polypeptide 6, alkali, smooth muscle and non-muscle	2,86
P62137	PPP1CA	Serine/threonine-protein phosphatase PP1-alpha catalytic subunit	2,86
P15306	THBD	Thrombomodulin	2,85
E0CXN1	FXYD5	FXYD domain-containing ion transport regulator	2,84
Q9CQB5	CISD2	CDGSH iron-sulfur domain-containing protein 2	2,82
Q9CQF3	NUDT21	Cleavage and polyadenylation specificity factor subunit 5	2,82
P23198	CBX3	Chromobox protein homolog 3	2,81
Q9CQQ7	ATP5PB	ATP synthase F(0) complex subunit B1, mitochondrial	2,80
Q9Z2B9	RPS6KA4	Ribosomal protein S6 kinase alpha-4	2,80
Q9DBG3	AP2B1	AP-2 complex subunit beta	2,79
Q8CFX3	PCDH1	Protocadherin 1	2,78
E9PZ21	RBMS1	RNA binding motif, single stranded interacting protein 1	2,78
Q62470	ITGA3	Integrin alpha-3	2,76
A2APM2	CD44	CD44 antigen	2,76
P62702	RPS4X	Small ribosomal subunit protein eS4	2,75
P52293	KPNA2	Importin subunit alpha-1	2,74
Q3TEA8	HP1BP3	Heterochromatin protein 1-binding protein 3	2,73
P11688	ITGA5	Integrin alpha-5	2,71
P62242	RPS8	Small ribosomal subunit protein eS8	2,70
Q9D8B3	CHMP4B	Charged multivesicular body protein 4b	2,70
Q61550	RAD21	Double-strand-break repair protein rad21 homolog	2,69
Q9CPS7	PNO1	RNA-binding protein PNO1	2,69
Q91VC3	EIF4A3	Eukaryotic initiation factor 4A-III	2,68
Q9DB15	MRPL12	Large ribosomal subunit protein bL12m	2,68
Q9CR80	FAM32A	Protein FAM32A	2,67
P26645	MARCKS	Myristoylated alanine-rich C-kinase substrate	2,67
Q8VCY6	UTP6	U3 small nucleolar RNA-associated protein 6 homolog	2,66
Q6NSP9	HMGA2	High mobility group AT-hook 2	2,66
Q9D5T0	ATAD1	Outer mitochondrial transmembrane helix translocase	2,65
Q8CCF0	PRPF31	U4/U6 small nuclear ribonucleoprotein Prp31	2,64
B1AT82	PRPSAP1	Phosphoribosyl pyrophosphate synthetase-associated protein 1	2,63
Q62093	SRSF2	Serine/arginine-rich splicing factor 2	2,63
B1AVB1	SCML2	Scm polycomb group protein like 2	2,62
Q9CQE8	RTRAF	RNA transcription, translation and transport factor protein	2,61
Q8BGD9	EIF4B	Eukaryotic translation initiation factor 4B	2,61
P47911	RPL6	Large ribosomal subunit protein eL6	2,61
P07091	S100A4	Protein S100-A4	2,60
Q9DB96	NGDN	Neuroguidin	2,60
O09131	GSTO1	Glutathione S-transferase omega-1	2,60
P62751	RPL23A	Large ribosomal subunit protein uL23	2,60
Q9ESX5	DKC1	H/ACA ribonucleoprotein complex subunit DKC1	2,60
O70318	EPB41L2	Band 4.1-like protein 2	2,59
Q8CI59	STEAP3	Metalloreductase STEAP3	2,59
G3X9Q6	LARP4	La ribonucleoprotein 4	2,59
O08709	PRDX6	Peroxiredoxin-6	2,59
Q8BHY2	NOC4L	Nucleolar complex protein 4 homolog	2,59
P53690	MMP14	Matrix metalloproteinase-14	2,57
Q8K094	PVR	Poliovirus receptor	2,56
Q99PL5	RRBP1	Ribosome-binding protein 1	2,56
Q99ME9	GTPBP4	GTP-binding protein 4	2,56
Q9JLV1	BAG3	BAG family molecular chaperone regulator 3	2,54
Q8R5L1	C1QBP	Complement component 1 Q subcomponent-binding protein, mitochondrial	2,53
Q91VR5	DDX1	ATP-dependent RNA helicase DDX1	2,53
Q9JKB3	YBX3	Y-box-binding protein 3	2,53
F8WGL3	CFL1	Cofilin 1, non-muscle	2,52
Q60598	CTTN	Src substrate cortactin	2,52
P47791	GSR	Glutathione reductase, mitochondrial	2,52
P60904	DNAJC5	DnaJ homolog subfamily C member 5	2,52
Q9DBG5	PLIN3	Perilipin-3	2,50
Q8BTS0	DDX5	Probable ATP-dependent RNA helicase DDX5	2,50
Q9DC63	FBXO3	F-box only protein 3	2,50
P62281	RPS11	Small ribosomal subunit protein uS17	2,49
P47962	RPL5	Large ribosomal subunit protein uL18	2,49
F7AA26	PAKAP	Paralemmin A kinase anchor protein (Fragment)	2,48
D3Z5I1	ZC3HAV1	Zinc finger CCCH type, antiviral 1	2,48
Q8BSL7	ARF2	ADP-ribosylation factor 2	2,48
Q8BMK4	CKAP4	Cytoskeleton-associated protein 4	2,47
Q9JIX8	ACIN1	Apoptotic chromatin condensation inducer in the nucleus	2,47
Q60749	KHDRBS1	KH domain-containing, RNA-binding, signal transduction-associated protein 1	2,47
Q8K224	NAT10	RNA cytidine acetyltransferase	2,47
Q9CPR4	RPL17	Large ribosomal subunit protein uL22	2,45
Q8VCI5	PEX19	Peroxisomal biogenesis factor 19	2,45
Q9D8S5	SRSF5	Serine and arginine-rich splicing factor 5	2,45
O70439	STX7	Syntaxin-7	2,44
P17225	PTBP1	Polypyrimidine tract-binding protein 1	2,43
P27546	MAP4	Microtubule-associated protein 4	2,43
P97351	RPS3A	Small ribosomal subunit protein eS1	2,43
P63321	RALA	Ras-related protein Ral-A	2,42
A0A0N4SW73	RAB11FIP5	RAB11 family interacting protein 5 (class I)	2,41
Q8R3H7	HS2ST1	Heparan sulfate 2-O-sulfotransferase 1	2,41
Q8BH74	NUP107	Nuclear pore complex protein Nup107	2,41
Q8BQ30	PPP1R18	Phostensin	2,40
Q99LI7	CSTF3	Cleavage stimulation factor subunit 3	2,40
Q9D903	EBNA1BP2	Probable rRNA-processing protein EBP2	2,39
Q80XU3	NUCKS1	Nuclear ubiquitous casein and cyclin-dependent kinase substrate 1	2,39
Q9D1D4	TMED10	Transmembrane emp24 domain-containing protein 10	2,39
P61211	ARL1	ADP-ribosylation factor-like protein 1	2,38
P41105	RPL28	Large ribosomal subunit protein eL28	2,38
S4R189	ARHGEF40	Rho guanine nucleotide exchange factor (GEF) 40	2,37
Q9JHJ0	TMOD3	Tropomodulin-3	2,36
Q91YR7	PRPF6	Pre-mRNA-processing factor 6	2,35
P62264	RPS14	Small ribosomal subunit protein uS11	2,35
P47963	RPL13	Large ribosomal subunit protein eL13	2,34
Q62446	FKBP3	Peptidyl-prolyl cis-trans isomerase FKBP3	2,34
P32067	SSB	Lupus La protein homolog	2,34
Q9D1J3	SARNP	SAP domain-containing ribonucleoprotein	2,34
Q8VDD5	MYH9	Myosin-9	2,34
Q60865	CAPRIN1	Caprin-1	2,33
P62754	RPS6	Small ribosomal subunit protein eS6	2,33
P53995	ANAPC1	Anaphase-promoting complex subunit 1	2,33
O35381	ANP32A	Acidic leucine-rich nuclear phosphoprotein 32 family member A	2,31
Q6DVA0	LEMD2	LEM domain-containing protein 2	2,31
P62245	RPS15A	Small ribosomal subunit protein uS8	2,31
P17047	LAMP2	Lysosome-associated membrane glycoprotein 2	2,31
Q91WK2	EIF3H	Eukaryotic translation initiation factor 3 subunit H	2,30
Q6NZB0	DNAJC8	DnaJ homolog subfamily C member 8	2,29
F8VQJ3	LAMC1	Laminin, gamma 1	2,28
P15864	H1-2	Histone H1.2	2,28
A0A286YDF5	MYOF	Myoferlin	2,28
E9Q634	MYO1E	Unconventional myosin-Ie	2,28
O08749	DLD	Dihydrolipoyl dehydrogenase, mitochondrial	2,28
Q99LP6	GRPEL1	GrpE protein homolog 1, mitochondrial	2,28
A0A1L1SVG6	SLC44A2	Choline transporter-like protein	2,27
Q922B2	DARS1	Aspartate--tRNA ligase, cytoplasmic	2,26
P13864	DNMT1	DNA (cytosine-5)-methyltransferase 1	2,26
Q9CXY6	ILF2	Interleukin enhancer-binding factor 2	2,25
A0A0R4J0T5	CELF1	CUGBP, Elav-like family member 1	2,25
Q5EBP8	HNRNPA1	Heterogeneous nuclear ribonucleoprotein A1	2,24
Q99P72	RTN4	Reticulon-4	2,24
P62911	RPL32	Large ribosomal subunit protein eL32	2,23
Q61792	LASP1	LIM and SH3 domain protein 1	2,23
P10922	H1-0	Histone H1.0	2,23
A0A1L1SRJ4	TSPAN3	Tetraspanin 3	2,22
P60867	RPS20	Small ribosomal subunit protein uS10	2,22
Q99KF1	TMED9	Transmembrane emp24 domain-containing protein 9	2,22
P62862	FAU	Ubiquitin-like FUBI-ribosomal protein eS30 fusion protein	2,22
O88532	ZFR	Zinc finger RNA-binding protein	2,22
P27046	MAN2A1	Alpha-mannosidase 2	2,22
Q05186	RCN1	Reticulocalbin-1	2,21
P51859	HDGF	Hepatoma-derived growth factor	2,20
Q9Z0J0	NPC2	NPC intracellular cholesterol transporter 2	2,20
Q8C3W1	1 SV	Uncharacterized protein C1orf198 homolog	2,20
Q3UI84	RFC4	Replication factor C (activator 1) 4	2,19
Q8K4P0	WDR33	pre-mRNA 3' end processing protein WDR33	2,19
B1APX2	5031439G07RIK	RIKEN cDNA 5031439G07 gene	2,18
Q61074	PPM1G	Protein phosphatase 1G	2,18
P70441	NHERF1	Na(+)/H(+) exchange regulatory cofactor NHE-RF1	2,17
Q9D7H3	RTCA	RNA 3'-terminal phosphate cyclase	2,17
P34022	RANBP1	Ran-specific GTPase-activating protein	2,17
Q91W50	CSDE1	Cold shock domain-containing protein E1	2,16
Q3UUU2	FUBP1	Far upstream element (FUSE) binding protein 1	2,15
Q8BJU0	SGTA	Small glutamine-rich tetratricopeptide repeat-containing protein alpha	2,15
Q9CRD0	OCIAD1	OCIA domain-containing protein 1	2,15
Q91XA2	GOLM1	Golgi membrane protein 1	2,14
O70126	AURKB	Aurora kinase B	2,14
P70670	NACA	Nascent polypeptide-associated complex subunit alpha, muscle-specific form	2,14
Q80UW8	POLR2E	DNA-directed RNA polymerases I, II, and III subunit RPABC1	2,13
Q6ZWX6	EIF2S1	Eukaryotic translation initiation factor 2 subunit 1	2,12
Q8VBT0	TMX1	Thioredoxin-related transmembrane protein 1	2,12
P14148	RPL7	Large ribosomal subunit protein uL30	2,12
Q8C854	MYEF2	Myelin expression factor 2	2,12
O55022	PGRMC1	Membrane-associated progesterone receptor component 1	2,11
P26516	PSMD7	26S proteasome non-ATPase regulatory subunit 7	2,11
A2ARP8	MAP1A	Microtubule-associated protein 1 A	2,11
P41731	CD63	CD63 antigen	2,11
G3X8R0	REEP5	Receptor expression-enhancing protein	2,10
Q80X50	UBAP2L	Ubiquitin-associated protein 2-like	2,09
Q3U741	DDX17	RNA helicase	2,09
P06795	ABCB1B	ATP-dependent translocase ABCB1	2,09
Q99NB8	UBQLN4	Ubiquilin-4	2,08
D3Z5F7	GM20521	Predicted gene 20521	2,08
Q9DCH6	ZFAND6	AN1-type zinc finger protein 6	2,08
Q99L47	ST13	Hsc70-interacting protein	2,07
O88384	VTI1B	Vesicle transport through interaction with t-SNAREs homolog 1B	2,07
Q3TZZ7	ESYT2	Extended synaptotagmin-2	2,06
Q9CZM2	RPL15	Large ribosomal subunit protein eL15	2,06
P62821	RAB1A	Ras-related protein Rab-1A	2,06
Q9JIM1	SLC29A1	Equilibrative nucleoside transporter 1	2,06
A0A1B0GRT6	PTPRE	Receptor-type tyrosine-protein phosphatase	2,06
P05202	GOT2	Aspartate aminotransferase, mitochondrial	2,06
Q8BIQ5	CSTF2	Cleavage stimulation factor subunit 2	2,05
Q9R1P3	PSMB2	Proteasome subunit beta type-2	2,05
P20108	PRDX3	Thioredoxin-dependent peroxide reductase, mitochondrial	2,05
Q02819	NUCB1	Nucleobindin-1	2,04
O08579	EMD	Emerin	2,04
P48962	SLC25A4	ADP/ATP translocase 1	2,04
Q64261	CDK6	Cyclin-dependent kinase 6	2,04
O08795	PRKCSH	Glucosidase 2 subunit beta	2,03
Q9Z0W3	NUP160	Nuclear pore complex protein Nup160	2,03
P97797	SIRPA	Tyrosine-protein phosphatase non-receptor type substrate 1	2,03
P57784	SNRPA1	U2 small nuclear ribonucleoprotein A'	2,03
O55142	RPL35A	Large ribosomal subunit protein eL33	2,02
Q7TSC1	PRRC2A	Protein PRRC2A	2,02
P99027	RPLP2	Large ribosomal subunit protein P2	2,02
P56959	FUS	RNA-binding protein FUS	2,00
Q8R3C6	RBM19	Probable RNA-binding protein 19	2,00
P51150	RAB7A	Ras-related protein Rab-7a	2,00

Supplemental table 5 : Summary table of proteins present in NIH-3T3-Src-Tks5-GFP cells seeded on collagen identified by mass spectrometry analysis of Tks5 interactome

Accession	Gene Name	Description	Abundance Ratio: (TKS5) / (Control)
O89032	SH3PXD2A	SH3 and PX domain-containing protein 2A	69,75
O55201	SUPT5H	Transcription elongation factor SPT5	34,69
Q9JM51	PTGES	Prostaglandin E synthase	31,66
Q61081	CDC37	Hsp90 co-chaperone Cdc37	22,03
D3YX43	VSIG10	V-set and immunoglobulin domain-containing protein 10	15,34
E9PUK3	FNBP1L	Formin-binding protein 1-like	15,07
O88839	ADAM15	Disintegrin and metalloproteinase domain-containing protein 15	14,15
Q9CXI5	MANF	Mesencephalic astrocyte-derived neurotrophic factor	14,12
Q9JKB3	YBX3	Y-box-binding protein 3	14,01
F8WIP8	SPP1	Secreted phosphoprotein 1	13,11
P47915	RPL29	Large ribosomal subunit protein eL29	11,74
E9QA15	CALD1	Caldesmon 1	11,71
A0A1B0GR85	2900026A02RIK	RIKEN cDNA 2900026A02 gene	11,64
O35674	ADAM19	Disintegrin and metalloproteinase domain-containing protein 19	11,11
E9Q9C3	AFDN	Afadin, adherens junction formation factor	9,65
E9Q450	TPM1	Tropomyosin 1, alpha	8,94
Q3TIV5	ZC3H15	Zinc finger CCCH domain-containing protein 15	8,88
D3YW42	CCDC9	Coiled-coil domain containing 9	8,78
Q8HWA3	ULBP1	UL16 binding protein 1	8,51
E9QK89	MDC1	Mediator of DNA damage checkpoint protein 1	8,49
O08539	BIN1	Myc box-dependent-interacting protein 1	8,36
Q61584	FXR1	RNA-binding protein FXR1	8,36
Q9QZM4	TNFRSF10B	Tumor necrosis factor receptor superfamily member 10B	8,20
Q80Y55	BSDC1	BSD domain-containing protein 1	8,06
Q61792	LASP1	LIM and SH3 domain protein 1	7,82
O70251	EEF1B	Elongation factor 1-beta	7,79
Q5F2E7	NUFIP2	FMR1-interacting protein NUFIP2	7,69
Q9JLB9	NECTIN3	Nectin-3	7,68
Q91XV3	BASP1	Brain acid soluble protein 1	7,68
Q99PA7	MAGEA13	MAGE family member A13	7,67
Q7TSJ2	MAP6	Microtubule-associated protein 6	7,45
Q91VX2	UBAP2	Ubiquitin-associated protein 2	7,39
P51125	CAST	Calpastatin	7,37
Q9JIX8	ACIN1	Apoptotic chromatin condensation inducer in the nucleus	7,31
Q6PGL7	WASHC2	WASH complex subunit 2	7,19
Q61490	ALCAM	CD166 antigen	6,85
D3YVW2	GOLIM4	Golgi integral membrane protein 4	6,77
P62960	YBX1	Y-box-binding protein 1	6,74
Q9WVC3	CAV2	Caveolin-2	6,61
P70452	STX4	Syntaxin-4	6,49
Q9D8S5	SRSF5	Serine and arginine-rich splicing factor 5	6,43
F6ZDS4	TPR	Nucleoprotein TPR	6,41
O89116	VTI1A	Vesicle transport through interaction with t-SNAREs homolog 1A	6,41
A0A286YDV7	NOLC1	Nucleolar and coiled-body phosphoprotein 1 (Fragment)	6,37
E9QA16	CALD1	Caldesmon 1	6,33
P26645	MARCKS	Myristoylated alanine-rich C-kinase substrate	6,30
Q9DBE9	FTSJ3	pre-rRNA 2'-O-ribose RNA methyltransferase FTSJ3	6,20
O54901	CD200	OX-2 membrane glycoprotein	6,18
Q8CI51	PDLIM5	PDZ and LIM domain protein 5	6,18
Q9ERB0	SNAP29	Synaptosomal-associated protein 29	6,15
Q8VBT6	APOBR	Apolipoprotein B receptor	6,04
P62141	PPP1CB	Serine/threonine-protein phosphatase PP1-beta catalytic subunit	5,98
O35226	PSMD4	26S proteasome non-ATPase regulatory subunit 4	5,90
P21995	EMB	Embigin	5,88
P63024	VAMP3	Vesicle-associated membrane protein 3	5,80
P40240	CD9	CD9 antigen	5,80
P47964	RPL36	Large ribosomal subunit protein eL36	5,68
Q3UDR8	YIPF3	Protein YIPF3	5,66
Q62093	SRSF2	Serine/arginine-rich splicing factor 2	5,65
P35762	CD81	CD81 antigen	5,53
P97298	SERPINF1	Pigment epithelium-derived factor	5,48
A0A0N4SW73	RAB11FIP5	RAB11 family interacting protein 5 (class I)	5,46
Q3THK3	GTF2F1	General transcription factor IIF subunit 1	5,42
P68254	YWHAQ	14-3-3 protein theta	5,39
Q8BQ30	PPP1R18	Phostensin	5,36
Q8K019	BCLAF1	Bcl-2-associated transcription factor 1	5,34
A0A1L1SSH9	SPARC	SPARC	5,32
Q4VAA2	CDV3	Protein CDV3	5,30
P22907	HMBS	Porphobilinogen deaminase	5,28
P62869	ELOB	Elongin-B	5,24
P27659	RPL3	Large ribosomal subunit protein uL3	5,19
Q3UM45	PPP1R7	Protein phosphatase 1 regulatory subunit 7	5,19
Q9WUK2	EIF4H	Eukaryotic translation initiation factor 4H	5,17
Q8BGD9	EIF4B	Eukaryotic translation initiation factor 4B	5,11
Q99NB8	UBQLN4	Ubiquilin-4	5,09
Q8BL66	EEA1	Early endosome antigen 1	5,08
A2AJI0	MAP7D1	MAP7 domain-containing protein 1	5,06
Q6A065	CEP170	Centrosomal protein of 170 kDa	5,05
Q8R1B4	EIF3C	Eukaryotic translation initiation factor 3 subunit C	5,04
P81117	NUCB2	Nucleobindin-2	5,00
Q501J7	PHACTR4	Phosphatase and actin regulator 4	5,00
P47968	RPIA	Ribose-5-phosphate isomerase	4,98
O35435	DHODH	Dihydroorotate dehydrogenase (quinone), mitochondrial	4,98
Q05D44	EIF5B	Eukaryotic translation initiation factor 5B	4,96
O88477	IGF2BP1	Insulin-like growth factor 2 mRNA-binding protein 1	4,87
Q9Z1Z0	USO1	General vesicular transport factor p115	4,86
E9Q6J5	BOD1L	Biorientation of chromosomes in cell division protein 1-like 1	4,86
Q3TLH4	PRRC2C	Protein PRRC2C	4,86
A2APM2	CD44	CD44 antigen	4,81
Q04887	SOX9	Transcription factor SOX-9	4,72
P00493	HPRT1	Hypoxanthine-guanine phosphoribosyltransferase	4,69
A0A668KLE1	LRRFIP2	Leucine rich repeat (in FLII) interacting protein 2	4,68
Q9Z315	SART1	U4/U6.U5 tri-snRNP-associated protein 1	4,62
O70318	EPB41L2	Band 4.1-like protein 2	4,61
Q9Z0J0	NPC2	NPC intracellular cholesterol transporter 2	4,61
Q9R1Q7	PLP2	Proteolipid protein 2	4,59
O54950	PRKAG1	5'-AMP-activated protein kinase subunit gamma-1	4,52
O08749	DLD	Dihydrolipoyl dehydrogenase, mitochondrial	4,51
Q9JLV1	BAG3	BAG family molecular chaperone regulator 3	4,47
P60904	DNAJC5	DnaJ homolog subfamily C member 5	4,44
Q6XLQ8	CALU	Calumenin	4,40
Q99PL5	RRBP1	Ribosome-binding protein 1	4,40
Q9R0A0	PEX14	Peroxisomal membrane protein PEX14	4,39
Q569Z6	THRAP3	Thyroid hormone receptor-associated protein 3	4,37
Q9D024	CCDC47	PAT complex subunit CCDC47	4,37
P15116	CDH2	Cadherin-2	4,36
Q9DBG5	PLIN3	Perilipin-3	4,36
Q5SF07	IGF2BP2	Insulin-like growth factor 2 mRNA-binding protein 2	4,34
P68510	YWHAH	14-3-3 protein eta	4,34
P62849	RPS24	Small ribosomal subunit protein eS24	4,32
A7YY80	EPB41L3	130kDa Protein 4.1B MEF cell isoform	4,31
F8WHT3	PRRC2B	Proline-rich coiled-coil 2B	4,31
Q9WUR9	AK4	Adenylate kinase 4, mitochondrial	4,30
Q5EG47	PRKAA1	5'-AMP-activated protein kinase catalytic subunit alpha-1	4,30
O70400	PDLIM1	PDZ and LIM domain protein 1	4,30
Q8K2F8	LSM14A	Protein LSM14 homolog A	4,26
O54998	FKBP7	Peptidyl-prolyl cis-trans isomerase FKBP7	4,26
Q9CY58	SERBP1	SERPINE1 mRNA-binding protein 1	4,26
Q3UMF0	COBLL1	Cordon-bleu protein-like 1	4,22
P61804	DAD1	Dolichyl-diphosphooligosaccharide--protein glycosyltransferase subunit DAD1	4,21
Q8R317	UBQLN1	Ubiquilin-1	4,21
Q62470	ITGA3	Integrin alpha-3	4,20
P54726	RAD23A	UV excision repair protein RAD23 homolog A	4,19
E9QM77	ATXN2	Ataxin 2	4,19
Q925B0	PAWR	PRKC apoptosis WT1 regulator protein	4,19
E9Q7Q3	TPM3	Tropomyosin 3, gamma	4,18
Q6IRU2	TPM4	Tropomyosin alpha-4 chain	4,18
E9Q8L9	RAB11FIP1	RAB11 family interacting protein 1 (class I)	4,17
Q80X50	UBAP2L	Ubiquitin-associated protein 2-like	4,12
A0A5K1VVQ9	TTN	Titin	4,11
P10639	TXN	Thioredoxin	4,08
O08602	RAET1A	Retinoic acid early-inducible protein 1-alpha	4,07
Q6IRU5	CLTB	Clathrin light chain B	4,06
Q3TGG2	ATXN2L	Ataxin 2-like	4,06
Q9R020	ZRANB2	Zinc finger Ran-binding domain-containing protein 2	4,06
Q8K094	PVR	Poliovirus receptor	4,01
Q3UL36	ARGLU1	Arginine and glutamate-rich protein 1	3,98
Q9DBR7	PPP1R12A	Protein phosphatase 1 regulatory subunit 12A	3,96
Q9QZM0	UBQLN2	Ubiquilin-2	3,93
Q7TSC1	PRRC2A	Protein PRRC2A	3,93
Q8BH97	RCN3	Reticulocalbin-3	3,92
Q62418	DBNL	Drebrin-like protein	3,91
O88569	HNRNPA2B1	Heterogeneous nuclear ribonucleoproteins A2/B1	3,86
F7AA26	PAKAP	Paralemmin A kinase anchor protein (Fragment)	3,85
Q6PGH2	JPT2	Jupiter microtubule associated homolog 2	3,85
Q80WW9	DDRGK1	DDRGK domain-containing protein 1	3,82
Q6TEK5	VKORC1L1	Vitamin K epoxide reductase complex subunit 1-like protein 1	3,80
Q05186	RCN1	Reticulocalbin-1	3,79
Q9EQ08	SGSH	Heparan N-sulfatase	3,76
P35492	HAL	Histidine ammonia-lyase	3,73
Q8CJ26	NRADD	Death domain-containing membrane protein NRADD	3,73
Q61335	BCAP31	B-cell receptor-associated protein 31	3,73
Q6P6J9	TXNDC15	Thioredoxin domain-containing protein 15	3,71
Q62446	FKBP3	Peptidyl-prolyl cis-trans isomerase FKBP3	3,70
P11087	COL1A1	Collagen alpha-1(I) chain	3,70
Q62011	PDPN	Podoplanin	3,66
A0A0R4J0U2	PPP4R2	Protein phosphatase 4, regulatory subunit 2	3,66
P49817	CAV1	Caveolin-1	3,65
Q6PFA2	CLTA	Clathrin light chain	3,65
P97287	MCL1	Induced myeloid leukemia cell differentiation protein Mcl-1 homolog	3,63
Q9CQR2	RPS21	Small ribosomal subunit protein eS21	3,63
Q3UJB0	SF3B2	Splicing factor 3b, subunit 2	3,60
Q0VAW6	8030462N17RIK	RIKEN cDNA 8030462N17 gene	3,59
Q8BIQ5	CSTF2	Cleavage stimulation factor subunit 2	3,58
Q922Q8	LRRC59	Leucine-rich repeat-containing protein 59	3,57
Q9CQE8	RTRAF	RNA transcription, translation and transport factor protein	3,57
Q9JKF6	NECTIN1	Nectin-1	3,56
Q80YR5	SAFB2	Scaffold attachment factor B2	3,56
O70439	STX7	Syntaxin-7	3,53
Q8C3W1	1 SV	Uncharacterized protein C1orf198 homolog	3,53
Q91VK1	BZW2	eIF5-mimic protein 1	3,52
Q9Z1A1	TFG	TFG protein	3,52
P08228	SOD1	Superoxide dismutase [Cu-Zn]	3,51
A0A0J9YUR2	SPECC1	Sperm antigen with calponin homology and coiled-coil domains 1	3,50
A0A0B4J1E2	SNW1	SNW domain-containing protein 1	3,49
Q8C9B9	DIDO1	Death-inducer obliterator 1	3,48
Q8K1N4	SPATS2	Spermatogenesis-associated serine-rich protein 2	3,48
Q61576	FKBP10	Peptidyl-prolyl cis-trans isomerase FKBP10	3,47
Q7TT18	ATF7IP	Activating transcription factor 7-interacting protein 1	3,47
Q02819	NUCB1	Nucleobindin-1	3,45
Q9CYA0	CRELD2	Protein disulfide isomerase Creld2	3,45
H3BL37	TCOF1	Treacle ribosome biogenesis factor 1	3,43
P47911	RPL6	Large ribosomal subunit protein eL6	3,42
Q64152	BTF3	Transcription factor BTF3	3,41
Q62314	TGOLN2	Trans-Golgi network integral membrane protein 2	3,39
P28667	MARCKSL1	MARCKS-related protein	3,38
Q8R3Q6	MIX23	Protein MIX23	3,38
Q8K1I7	WIPF1	WAS/WASL-interacting protein family member 1	3,37
O08583	ALYREF	THO complex subunit 4	3,36
P11438	LAMP1	Lysosome-associated membrane glycoprotein 1	3,35
Q5SUH6	CLINT1	Clathrin interactor 1	3,33
P14873	MAP1B	Microtubule-associated protein 1B	3,32
P10518	ALAD	Delta-aminolevulinic acid dehydratase	3,32
Q99JF8	PSIP1	PC4 and SFRS1-interacting protein	3,31
Q60598	CTTN	Src substrate cortactin	3,31
Q3UZ39	LRRFIP1	Leucine-rich repeat flightless-interacting protein 1	3,31
A0A3B2WCL5	RANBP3	RAN binding protein 3	3,29
P97797	SIRPA	Tyrosine-protein phosphatase non-receptor type substrate 1	3,29
P14148	RPL7	Large ribosomal subunit protein uL30	3,27
P55302	LRPAP1	Alpha-2-macroglobulin receptor-associated protein	3,25
Q8K298	ANLN	Anillin	3,25
P51859	HDGF	Hepatoma-derived growth factor	3,25
Q9ES97	RTN3	Reticulon-3	3,24
O55022	PGRMC1	Membrane-associated progesterone receptor component 1	3,22
J3QPG5	PSAP	Prosaposin	3,21
P56959	FUS	RNA-binding protein FUS	3,21
Q20BD0	HNRNPAB	Heterogeneous nuclear ribonucleoprotein A/B	3,20
Q7TMK9	SYNCRIP	Heterogeneous nuclear ribonucleoprotein Q	3,20
Q62165	DAG1	Dystroglycan 1	3,19
Q61937	NPM1	Nucleophosmin	3,19
P27546	MAP4	Microtubule-associated protein 4	3,19
Q8R143	PTTG1IP	Pituitary tumor-transforming gene 1 protein-interacting protein	3,18
Q3U9N4	GRN	Granulin	3,17
Q9QYE6	GOLGA5	Golgin subfamily A member 5	3,17
O35887	CALU	Calumenin	3,15
Q6PCQ6	EFCAB14	EF-hand calcium binding domain 14	3,14
P48036	ANXA5	Annexin A5	3,14
P54728	RAD23B	UV excision repair protein RAD23 homolog B	3,14
Q9JL35	HMGN5	High mobility group nucleosome-binding domain-containing protein 5	3,12
Q61810	LTBP3	Latent-transforming growth factor beta-binding protein 3	3,11
Q01149	COL1A2	Collagen alpha-2(I) chain	3,10
Q8VDD5	MYH9	Myosin-9	3,08
P09671	SOD2	Superoxide dismutase [Mn], mitochondrial	3,08
P70441	NHERF1	Na(+)/H(+) exchange regulatory cofactor NHE-RF1	3,07
Q3UH59	MYH10	Myosin, heavy polypeptide 10, non-muscle	3,06
A2AMH4	SLC44A1	Choline transporter-like protein	3,06
Q91XA2	GOLM1	Golgi membrane protein 1	3,04
Q8K201	KCT2	Keratinocyte-associated transmembrane protein 2	3,04
Q9WVE8	PACSIN2	Protein kinase C and casein kinase substrate in neurons protein 2	3,03
P84099	RPL19	Large ribosomal subunit protein eL19	3,01
Q9CZM2	RPL15	Large ribosomal subunit protein eL15	3,01
Q9DAU1	CNPY3	Protein canopy homolog 3	3,00
P61982	YWHAG	14-3-3 protein gamma	2,98
O88271	CFDP1	Craniofacial development protein 1	2,98
Q61074	PPM1G	Protein phosphatase 1G	2,97
P17809	SLC2A1	Solute carrier family 2, facilitated glucose transporter member 1	2,97
P97300	NPTN	Neuroplastin	2,97
Q6NZB0	DNAJC8	DnaJ homolog subfamily C member 8	2,96
A0A1D5RM85	RPL18A	Ribosomal protein L18A (Fragment)	2,96
P62137	PPP1CA	Serine/threonine-protein phosphatase PP1-alpha catalytic subunit	2,95
Q8BLN5	LSS	Lanosterol synthase	2,94
Q8K4Q8	COLEC12	Collectin-12	2,93
Q9CQF9	PCYOX1	Prenylcysteine oxidase 1	2,92
Q8C0E2	VPS26B	Vacuolar protein sorting-associated protein 26B	2,92
P97808	FXYD5	FXYD domain-containing ion transport regulator 5	2,92
P14869	RPLP0	Large ribosomal subunit protein uL10	2,92
P41731	CD63	CD63 antigen	2,92
Q8R4U7	LUZP1	Leucine zipper protein 1	2,91
P70399	TP53BP1	TP53-binding protein 1	2,90
Q9CR60	GOLT1B	Vesicle transport protein GOT1B	2,89
Q9WV80	SNX1	Sorting nexin-1	2,89
P43276	H1-5	Histone H1.5	2,89
P62962	PFN1	Profilin-1	2,89
Q61425	HADH	Hydroxyacyl-coenzyme A dehydrogenase, mitochondrial	2,89
Q8R550	SH3KBP1	SH3 domain-containing kinase-binding protein 1	2,88
Q9CQK7	RWDD1	RWD domain-containing protein 1	2,88
P62918	RPL8	Large ribosomal subunit protein uL2	2,88
Q62159	RHOC	Rho-related GTP-binding protein RhoC	2,88
O35381	ANP32A	Acidic leucine-rich nuclear phosphoprotein 32 family member A	2,88
Q00623	APOA1	Apolipoprotein A-I	2,87
A0A668KL36	CCDC50	Coiled-coil domain containing 50	2,87
Q3UHX2	PDAP1	28 kDa heat- and acid-stable phosphoprotein	2,87
P47962	RPL5	Large ribosomal subunit protein uL18	2,86
Q66JS6	EIF3J2	Eukaryotic translation initiation factor 3 subunit J-B	2,85
Q5U3K5	RABL6	Rab-like protein 6	2,85
Q8BP27	SFR1	Swi5-dependent recombination DNA repair protein 1 homolog	2,85
Q569Z5	DDX46	Probable ATP-dependent RNA helicase DDX46	2,85
Q9CZ44	NSFL1C	NSFL1 cofactor p47	2,85
Q80WJ7	MTDH	Protein LYRIC	2,84
Q8K1L5	PPP1R11	E3 ubiquitin-protein ligase PPP1R11	2,83
Q9CZH7	MXRA7	Matrix-remodeling-associated protein 7	2,83
E9Q1P8	IRF2BP2	Interferon regulatory factor 2-binding protein 2	2,82
P32067	SSB	Lupus La protein homolog	2,82
Q9Z0P4	PALM	Paralemmin-1	2,81
P14211	CALR	Calreticulin	2,81
Q8CHP5	PYM1	Partner of Y14 and mago	2,80
P12970	RPL7A	Large ribosomal subunit protein eL8	2,79
P09103	P4HB	Protein disulfide-isomerase	2,79
Q9CPV5	PMF1	Polyamine-modulated factor 1	2,78
Q5SUS9	EWSR1	Ewing sarcoma breakpoint region 1	2,78
Q6P8I4	PCNP	PEST proteolytic signal-containing nuclear protein	2,78
P17047	LAMP2	Lysosome-associated membrane glycoprotein 2	2,77
Q80XU3	NUCKS1	Nuclear ubiquitous casein and cyclin-dependent kinase substrate 1	2,76
P11440	CDK1	Cyclin-dependent kinase 1	2,76
Q9D2G2	DLST	Dihydrolipoyllysine-residue succinyltransferase component of 2-oxoglutarate dehydrogenase complex, mitochondrial	2,75
G3X8R0	REEP5	Receptor expression-enhancing protein	2,74
Q99P72	RTN4	Reticulon-4	2,74
P47963	RPL13	Large ribosomal subunit protein eL13	2,74
O70503	HSD17B12	Very-long-chain 3-oxoacyl-CoA reductase	2,73
O88384	VTI1B	Vesicle transport through interaction with t-SNAREs homolog 1B	2,72
Q9WVK4	EHD1	EH domain-containing protein 1	2,71
Q9CQV8	YWHAB	14-3-3 protein beta/alpha	2,70
P46061	RANGAP1	Ran GTPase-activating protein 1	2,69
Q9JMI7	TEX101	Testis-expressed protein 101	2,68
O70401	TSPAN6	Tetraspanin-6	2,68
P12265	GUSB	Beta-glucuronidase	2,66
A0A1B0GS68	GM45713	60S ribosomal protein L13a	2,66
O08579	EMD	Emerin	2,66
A2AUD5	TPD52L2	Tumor protein D52-like 2	2,64
P18572	BSG	Basigin	2,64
P62259	YWHAE	14-3-3 protein epsilon	2,64
Q8BXV2	BRI3BP	BRI3-binding protein	2,64
Q80UU9	PGRMC2	Membrane-associated progesterone receptor component 2	2,64
Q63918	CAVIN2	Caveolae-associated protein 2	2,63
P70670	NACA	Nascent polypeptide-associated complex subunit alpha, muscle-specific form	2,62
Q9D8B3	CHMP4B	Charged multivesicular body protein 4b	2,61
Q8BGT7	SMNDC1	Survival of motor neuron-related-splicing factor 30	2,61
P17225	PTBP1	Polypyrimidine tract-binding protein 1	2,61
P26041	MSN	Moesin	2,61
P16045	LGALS1	Galectin-1	2,61
Q8BG05	HNRNPA3	Heterogeneous nuclear ribonucleoprotein A3	2,61
Q921T2	TOR1AIP1	Torsin-1A-interacting protein 1	2,61
P59999	ARPC4	Actin-related protein 2/3 complex subunit 4	2,61
P63101	YWHAZ	14-3-3 protein zeta/delta	2,61
Q4PZA2	ECE1	Endothelin-converting enzyme 1	2,60
P32020	SCP2	Sterol carrier protein 2	2,57
Q8VCI5	PEX19	Peroxisomal biogenesis factor 19	2,56
Q8R409	HEXIM1	Protein HEXIM1	2,56
P97371	PSME1	Proteasome activator complex subunit 1	2,56
Q9R0X4	ACOT9	Acyl-coenzyme A thioesterase 9, mitochondrial	2,56
Q9D8E6	RPL4	Large ribosomal subunit protein uL4	2,55
P50543	S100A11	Protein S100-A11	2,55
P63276	RPS17	Small ribosomal subunit protein eS17	2,54
Q99LX0	PARK7	Parkinson disease protein 7 homolog	2,54
Q64314	CD34	Hematopoietic progenitor cell antigen CD34	2,54
Q91Z38	TTC1	Tetratricopeptide repeat protein 1	2,53
Q8C129	LNPEP	Leucyl-cystinyl aminopeptidase	2,52
Q6NZJ6	EIF4G1	Eukaryotic translation initiation factor 4 gamma 1	2,52
A0A286YCG8	SSR1	Translocon-associated protein subunit alpha	2,51
Q6P5H2	NES	Nestin	2,49
Q9Z0L0	TPBG	Trophoblast glycoprotein	2,48
Q8BM72	HSPA13	Heat shock 70 kDa protein 13	2,47
F8WIV2	SERPINB6A	Serine (or cysteine) peptidase inhibitor, clade B, member 6a	2,47
A0A087WPK3	LRRFIP1	Leucine rich repeat (in FLII) interacting protein 1	2,46
A0A571BF54	KCNAB2	Voltage-gated potassium channel subunit beta-1	2,46
Q8BQ47	CNPY4	Protein canopy homolog 4	2,46
Q61166	MAPRE1	Microtubule-associated protein RP/EB family member 1	2,45
Q5EBP8	HNRNPA1	Heterogeneous nuclear ribonucleoprotein A1	2,44
P29341	PABPC1	Polyadenylate-binding protein 1	2,42
P20108	PRDX3	Thioredoxin-dependent peroxide reductase, mitochondrial	2,42
P62897	CYCS	Cytochrome c, somatic	2,42
O35685	NUDC	Nuclear migration protein nudC	2,42
Q9JKR6	HYOU1	Hypoxia up-regulated protein 1	2,42
P62754	RPS6	Small ribosomal subunit protein eS6	2,42
P97855	G3BP1	Ras GTPase-activating protein-binding protein 1	2,42
Q9QXS6	DBN1	Drebrin	2,42
A2ARP8	MAP1A	Microtubule-associated protein 1 A	2,40
H7BX95	SRSF1	Serine/arginine-rich splicing factor 1	2,39
P19324	SERPINH1	Serpin H1	2,39
Q9Z2B9	RPS6KA4	Ribosomal protein S6 kinase alpha-4	2,38
P35980	RPL18	Large ribosomal subunit protein eL18	2,38
P07141	CSF1	Macrophage colony-stimulating factor 1	2,38
A0A7N9VR94	AHNAK2	AHNAK nucleoprotein 2	2,38
E9Q0F0	KRT78	Keratin 78	2,38
Q62356	FSTL1	Follistatin-related protein 1	2,38
P61290	PSME3	Proteasome activator complex subunit 3	2,37
Q9D8X2	CCDC124	Coiled-coil domain-containing protein 124	2,36
Q9D1R9	RPL34	Large ribosomal subunit protein eL34	2,36
Q9D3L3	SNAP23	Synaptosomal-associated protein	2,36
Q8R180	ERO1A	ERO1-like protein alpha	2,35
G3XA48	IDI1	isopentenyl-diphosphate Delta-isomerase	2,35
P62911	RPL32	Large ribosomal subunit protein eL32	2,35
O08795	PRKCSH	Glucosidase 2 subunit beta	2,34
P26516	PSMD7	26S proteasome non-ATPase regulatory subunit 7	2,34
P62242	RPS8	Small ribosomal subunit protein eS8	2,34
Q64433	HSPE1	10 kDa heat shock protein, mitochondrial	2,33
P25444	RPS2	Small ribosomal subunit protein uS5	2,32
E9QAQ7	ARID1A	AT rich interactive domain 1A (SWI-like)	2,31
Q8VEK3	HNRNPU	Heterogeneous nuclear ribonucleoprotein U	2,31
P53690	MMP14	Matrix metalloproteinase-14	2,30
A0A3Q4EGK3	LTBP1	Latent transforming growth factor beta binding protein 1	2,30
Q8BSY0	ASPH	Aspartyl/asparaginyl beta-hydroxylase	2,30
Q9ER00	STX12	Syntaxin-12	2,29
Q9CR57	RPL14	Large ribosomal subunit protein eL14	2,29
P29391	FTL1	Ferritin light chain 1	2,29
F8VQC7	KTN1	Kinectin 1	2,28
E9QM38	SLC12A2	Solute carrier family 12, member 2	2,26
A2A5S3	STAU1	Staufen double-stranded RNA binding protein 1	2,26
Q3UEB3	PUF60	Poly(U)-binding-splicing factor PUF60	2,24
P32261	SERPINC1	Antithrombin-III	2,24
O88983	STX8	Syntaxin-8	2,24
Q9DBJ1	PGAM1	Phosphoglycerate mutase 1	2,23
Q9D1J3	SARNP	SAP domain-containing ribonucleoprotein	2,23
Q8BJU0	SGTA	Small glutamine-rich tetratricopeptide repeat-containing protein alpha	2,23
P42208	SEPTIN2	Septin-2	2,22
Q9JIH2	NUP50	Nuclear pore complex protein Nup50	2,22
Q8BL97	SRSF7	Serine/arginine-rich splicing factor 7	2,22
P35456	PLAUR	Urokinase plasminogen activator surface receptor	2,21
P86048	RPL10L	Large ribosomal subunit protein uL16-like	2,21
F2Z456	CYB5R3	NADH-cytochrome b5 reductase	2,20
P09055	ITGB1	Integrin beta-1	2,20
B2RXS4	PLXNB2	Plexin-B2	2,20
P97822	ANP32E	Acidic leucine-rich nuclear phosphoprotein 32 family member E	2,20
Q99K48	NONO	Non-POU domain-containing octamer-binding protein	2,20
Q91YR1	TWF1	Twinfilin-1	2,20
Q80UG5	SEPTIN9	Septin-9	2,19
Q9Z1R4	D17H6S53E	Uncharacterized protein C6orf47 homolog	2,18
P23116	EIF3A	Eukaryotic translation initiation factor 3 subunit A	2,18
Q8BR63	FAM177A1	Protein FAM177A1	2,18
Q9Z247	FKBP9	Peptidyl-prolyl cis-trans isomerase FKBP9	2,18
Q3UUU2	FUBP1	Far upstream element (FUSE) binding protein 1	2,17
Q9CZU6	CS	Citrate synthase, mitochondrial	2,16
G3X9Q6	LARP4	La ribonucleoprotein 4	2,16
Q8K021	SCAMP1	Secretory carrier-associated membrane protein 1	2,15
P16110	LGALS3	Galectin-3	2,15
Q5XJF6	RPL10A	Ribosomal protein	2,13
Q01730	RSU1	Ras suppressor protein 1	2,13
P05201	GOT1	Aspartate aminotransferase, cytoplasmic	2,12
V9GXM1	ARFGAP1	ADP-ribosylation factor GTPase activating protein 1	2,12
Q3UV17	KRT76	Keratin, type II cytoskeletal 2 oral	2,11
P11688	ITGA5	Integrin alpha-5	2,11
A2CEK3	PGM1	Phosphoglucomutase 1	2,11
Q8C1D8	IWS1	Protein IWS1 homolog	2,10
Q8C845	EFHD2	EF hand domain containing 2	2,10
Q8R5L1	C1QBP	Complement component 1 Q subcomponent-binding protein, mitochondrial	2,10
Q3UMU9	HDGFL2	Hepatoma-derived growth factor-related protein 2	2,09
P47791	GSR	Glutathione reductase, mitochondrial	2,09
P80314	CCT2	T-complex protein 1 subunit beta	2,08
A0A0R4J083	ACADL	Long-chain specific acyl-CoA dehydrogenase, mitochondrial	2,08
P15864	H1-2	Histone H1.2	2,07
Q99N15	HSD17B10	17beta-hydroxysteroid dehydrogenase type 10/short chain L-3-hydroxyacyl-CoA dehydrogenase	2,07
A0A1L1SRJ4	TSPAN3	Tetraspanin 3	2,06
Q9JLQ0	CD2AP	CD2-associated protein	2,06
A0A0R4J1E2	EEF1D	Eukaryotic translation elongation factor 1 delta (guanine nucleotide exchange protein)	2,05
A0A0J9YUL3	SEPTIN11	Septin	2,05
P62702	RPS4X	Small ribosomal subunit protein eS4	2,05
P04104	KRT1	Keratin, type II cytoskeletal 1	2,05
Q9JJG0	TACC2	Transforming acidic coiled-coil-containing protein 2	2,04
G5E866	SF3B1	Splicing factor 3b, subunit 1	2,04
Q9CPR4	RPL17	Large ribosomal subunit protein uL22	2,04
O35566	CD151	CD151 antigen	2,03
Q7TNV0	DEK	Protein DEK	2,02
P37913	LIG1	DNA ligase 1	2,02
P15306	THBD	Thrombomodulin	2,02
A0A5F8MPS7	ACP2	Acid phosphatase 2, lysosomal	2,02
Q920A5	SCPEP1	Retinoid-inducible serine carboxypeptidase	2,01
A0A1B0GR11	TALDO1	Transaldolase	2,01
P47757	CAPZB	F-actin-capping protein subunit beta	2,00

Supplemental table 6 : Summary table of the references used to classify the list of 88 common proteins as validated or not in invadosomes.

Proteins	References
ADAM15	The podosomal-adaptor protein SH3PXD2B is essential for normal postnatal development - Mao et *al.*, 2009
ADAM19	• Aspartate β-hydroxylase promotes pancreatic ductal adenocarcinoma metastasis through activation of SRC signaling pathway - Ogawa et *al.*, 2019<br>• The Adaptor Protein Fish Associates with Members of the ADAMs Family and Localizes to Podosomes of Src-transformed Cells - Abram et *al.*, 2003
ASPH	Aspartate β-hydroxylase promotes pancreatic ductal adenocarcinoma metastasis through activation of SRC signaling pathway - Ogawa et *al.*, 2019
BAG3	Combining laser capture microdissection and proteomics reveals an active translation machinery controlling invadosome formation - Ezzoukhry et *al.*, 2018
CALD1	• Caldesmon is an integral component of podosomes in smooth muscle cells - Eves et *al.*, 2006<br>• Caldesmon is an integral component of podosomes in smooth muscle cells - Gu et *al.*, 2007<br>• Changes in the balance between caldesmon regulated by p21-activated kinases and the Arp2/3 complex govern podosome formation - Morita et *al.*, 2007
CD44	• The CD44s splice isoform is a central mediator for invadopodia activity - Zhao et *al.*, 2016<br>• CD147, CD44, and the Epidermal Growth Factor Receptor (EGFR) Signaling Pathway Cooperate to Regulate Breast Epithelial Cell Invasiveness - Grass et *al.*, 2013<br>• CD44 and beta3 integrin organize two functionally distinct actin-based domains in osteoclasts - Chabadel et *al.*, 2007<br>• Macrophages podosomes go 3 - Goethem et *al.*, 2011
CTTN	• An invasion-related complex of cortactin, paxillin and PKCmu associates with invadopodia at sites of extracellular matrix degradation - Bowden et *al.*, 1999<br>• ERβ promoted invadopodia formation-mediated non-small cell lung cancer metastasis via the ICAM1/p-Src/p-Cortactin signaling pathway - Wang et *al.*, 2023
EIF4B	Combining laser capture microdissection and proteomics reveals an active translation machinery controlling invadosome formation - Ezzoukhry et *al.*, 2018
FNBP1L	Transducer of Cdc42-dependent actin assembly promotes breast cancer invasion and metastasis - Chander et *al.*, 2013
FXR1	Combining laser capture microdissection and proteomics reveals an active translation machinery controlling invadosome formation - Ezzoukhry et *al.*, 2018
G3BP1	Combining laser capture microdissection and proteomics reveals an active translation machinery controlling invadosome formation - Ezzoukhry et *al.*, 2018
HNRNPA1	Combining laser capture microdissection and proteomics reveals an active translation machinery controlling invadosome formation - Ezzoukhry et *al.*, 2018
IGF2BP2	IMP2 and IMP3 cooperate to promote the metastasis of triple-negative breast cancer through destabilization of progesterone receptor - Kim et *al.*, 2018
ITGA5	Membrane Proteome Analysis of Glioblastoma Cell Invasion - Mallawaaratchy et *al.*, 2015
LAMP1	Lysosomal cathepsin B participates in the podosome-mediated extracellular matrix degradation and invasion via secreted lysosomes in v-Src fibroblasts - Chun Tu et *al.*, 2008
MAP4	A proximity-labeling proteomic approach to investigate invadopodia molecular landscape in breast cancer cells - Thuault et *al.*, 2020
MMP14	Identification of the membrane-type matrix metalloproteinase MT1-MMP in osteoclasts - Sato et al., 1997<br>Dynamic interactions of cortactin and membrane type 1 matrix metalloproteinase at invadopodia: defining the stages of invadopodia formation and function - Artym et *al.*, 2008
MYH9	• TRPM7, a novel regulator of actomyosin contractility and cell adhesion 6 - Clarck et *al.*, 2006<br>• Bradykinin promotes migration and invasion of hepatocellular carcinoma cells through TRPM7 and MMP2 - Chen et *al.*, 2016
NONO	Combining laser capture microdissection and proteomics reveals an active translation machinery controlling invadosome formation - Ezzoukhry et *al.*, 2018
NPM1	Combining laser capture microdissection and proteomics reveals an active translation machinery controlling invadosome formation - Ezzoukhry et *al.*, 2018
PABPC1	Combining laser capture microdissection and proteomics reveals an active translation machinery controlling invadosome formation - Ezzoukhry et *al.*, 2018
PPP1CA	Combining laser capture microdissection and proteomics reveals an active translation machinery controlling invadosome formation - Ezzoukhry et *al.*, 2018
PRKAA1	A proximity-labeling proteomic approach to investigate invadopodia molecular landscape in breast cancer cells - Thuault et *al.*, 2020
PTBP1	The lncRNA MIR99AHG directs alternative splicing of SMARCA1 by PTBP1 to enable invadopodia formation in colorectal cancer cells - Li et *al.*, 2023
RPL10A	Combining laser capture microdissection and proteomics reveals an active translation machinery controlling invadosome formation, Ezzoukhry et *al.*, 2018
RPL34	Combining laser capture microdissection and proteomics reveals an active translation machinery controlling invadosome formation - Ezzoukhry et *al.*, 2018
RPS4X	Combining laser capture microdissection and proteomics reveals an active translation machinery controlling invadosome formation - Ezzoukhry et *al.*, 2018
RRBP1	Combining laser capture microdissection and proteomics reveals an active translation machinery controlling invadosome formation - Ezzoukhry et *al.*, 2018
RTN4	A proximity-labeling proteomic approach to investigate invadopodia molecular landscape in breast cancer cells - Thuault et *al.*, 2020
SSB	The PDGFRα-laminin B1-keratin 19 cascade drives tumor progression at the invasive front of human hepatocellular carcinoma - Govaere et *al.*, 2017
STX7	Syntaxin 7 contributes to breast cancer cell invasion by promoting invadopodia formation - Parveen et *al.*, 2022
SYNCRIP	Combining laser capture microdissection and proteomics reveals an active translation machinery controlling invadosome formation - Ezzoukhry et *al.*, 2018
THBD	VEGF-Induced Endothelial Podosomes via ROCK2-Dependent Thrombomodulin Expression Initiate Sprouting Angiogenesis - Cheng-Hsiang Kuo et *al.*, 2021
YBX3	Combining laser capture microdissection and proteomics reveals an active translation machinery controlling invadosome formation - Ezzoukhry et *al.*, 2018
