# Supplementary material for: Tks5 interactome reveals endoplasmic‐reticulum‐associated translation machinery in invadosomes
Source: FEBS J. 2025 Jul 24;292(23):6400–19. doi: 10.1111/febs.70196 (PMC12699180; doi:10.1111/febs.70196)
Supplement: Supplementary file 1 — Fig. S1. Specific molecular proteins of each invadosomes organization. Fig. S2. Characterization and validation of proteins identified by mass spectrometry as partner of Tks5. Fig. S3. Translation is not involved on invadosome formation. Table S1. Summary table of proteins present in invadosomes identified by mass spectrometry analysis of Tks 5 interactome. Table S2. Summary table of proteins present in A 431 Tks 5 GFP cells seeded on plastic identified by mass spectrometry analysis of Tks 5 interactome. Table S3. Summary table of proteins present in A 431 Tks 5 GFP cells seeded on collagen identified by mass spectrometry analysis of Tks 5 interactome. Table S4. Summary table of proteins present in NIH 3 T 3 Src Tks 5 GFP cells seeded on plastic identified by mass spectrometry analysis of Tks 5 interactome. Table S5. Summary table of proteins present in NIH 3 T 3 Src Tks 5 GFP cells seeded on collagen identified by mass spectrometry analysis of Tks 5 interactome. Table S6. Summary table of the references used to classify the list of 88 common proteins as validated or not in invadosomes. [file FEBS-292-6400-s006.zip › Supplementary Figure Legends.pdf]

Supp Figure 1: Specific molecular proteins of each invadosomes organization.
a) Western Blot analysis of the co-immunoprecipitation performed with anti-GFP antibodies in NIH3T3-Src-Tks5-GFP cells. The input, the Δ and the co-IP (IP-Tks5-GFP) fractions are shown. **b)** Bubble plot of the proteins related pathway in rosettes. **c)** Classification table of the specific proteins identified in rosette. Proteins are grouped into five main categories corresponding to translation proteins, mitochondrial proteins, ER proteins, proteins of the Golgi apparatus and microtubules proteins. **d)** Bubble plot of the proteins related pathway in dots. **e)** Classification table of the specific proteins identified in dots. Proteins are grouped into five main categories corresponding to translation proteins, mitochondrial proteins, ER proteins, proteins of the Golgi apparatus and microtubules proteins. **f)** Bubble plot of the proteins related pathway in linear invadosomes and classification table of the adhesion proteins specific to linear invadosomes organizations. **g)** Classification table of the specific proteins identified in linear invadosomes. Proteins are grouped into five main categories corresponding to translation proteins, mitochondrial proteins, ER proteins, proteins of the Golgi apparatus and microtubules proteins.
ER : endoplasmic reticulum

Supp Figure 2: Characterization and validation of proteins identified by mass spectrometry as partner of Tks5.
a) Table of proteins identified by mass spectrometry in at least one type of invadosomes and already identified in the literature as partner of Tks5 in 293T cells (Stylli and *al.*) and MDA-MB-231 cells (Thuault and *al.*). **b)** Confocal microscopy images of A431-Tks5-GFP cells. The cells were seeded on gelatin or type I collagen and stained for Tks5 in green, actin in red, nuclei in blue and MAP4 in grey. Scale bar: 40µm, zoom: 5µm. **c)** Classification table of the 88 common proteins identified. Proteins are grouped into three main categories corresponding to translation, adhesion and actin cytoskeleton. **d)** Table of translation proteins already identified in invadosomes in the literature **e)** Bubble plot of proteins related pathway identified by mass spectrometry thanks to Tks5 and present in data already published by Ezzoukhry and *al.* in rosettes. **f)** Summary table of crossed data obtained by mass spectrometry for the rosettes with the data from Ezzoukhry and *al.* paper.

Supp Figure 3: Translation is not involved on invadosome formation.
a) Quantification of the effect of cycloheximide treatment on A431 and NIH-3T3-Src cells by using puromycin quantification. Values represent the mean +/- SEM of n=4 independent experiments and were analyzed using student t-test. **b)** Relative western blot analysis of puromycin expression for cells treated (CHX) or not (DMSO) with cycloheximide in A431 and NIH-3T3-Src cells seeded on gelatin or collagen. **c)** Quantification of the numbers of invadosomes per cell on gelatin and collagen treated (CHX) or not (DMSO) with cycloheximide in A431-Tks5-GFP and NIH3T3-Src-Tks5-GFP cells. Values represent the mean +/- SEM of n=3 independent experiments (10 images per condition and per replicate) and were analyzed using student t-test. **d)** Representative images of invadosome formation in A431-Tks5-GFP1 and NIH3T3-Src-Tks5-GFP cells seeded on gelatin or collagen. Tks5 is stained in green and nuclei in blue. Scale bar: 40µm, zoom: 10µm. **e)** Western Blot analysis of endogenous EIF4B expression in A431 and NIH-3T3-Src cells transfected using control or EIF4B-targeting siRNA and associated quantification. Stain free was used as the loading control. Values represent the mean of n=4 independent experiments. **f)** Quantification of the numbers of invadosomes per cell on gelatin and collagen silencing (siEIF4B) or not (DMSO) for EIF4B in A431-Tks5-GFP and NIH3T3-Src-Tks5-GFP cells. Values represent the mean +/- SEM of n=4 independent experiments (10 images per condition and per replicate) and were analyzed using student t-test.
CHX : cycloheximide ; DMSO : dimethyl sulfoxide , EIF4B : eukaryotic translation factor 4B.
